# Interpersonal touch interventions for patients in intensive care: A design‐oriented realist review

**DOI:** 10.1002/nop2.200

**Published:** 2018-10-24

**Authors:** Sansha J. Harris, Elizabeth D. E. Papathanassoglou, Melanie Gee, Susan M. Hampshaw, Lenita Lindgren, Annette Haywood

**Affiliations:** ^1^ School of Health and Related Research (ScHARR) University of Sheffield Sheffield UK; ^2^ Faculty of Nursing University of Alberta Edmonton Alberta Canada; ^3^ Faculty of Health and Wellbeing Sheffield Hallam University Sheffield UK; ^4^ Department of Nursing Umeå University Umeå Sweden

**Keywords:** design propositions, hypnotics and sedatives, ICU, nursing, pain, realist review, stress, touch

## Abstract

**Aim:**

To develop a theoretical framework to inform the design of interpersonal touch interventions intended to reduce stress in adult intensive care unit patients.

**Design:**

Realist review with an intervention design‐oriented approach.

**Methods:**

We searched CINAHL, MEDLINE, EMBASE, CENTRAL, Web of Science and grey literature sources without date restrictions. Subject experts suggested additional articles. Evidence synthesis drew on diverse sources of literature and was conducted iteratively with theory testing. We consulted stakeholders to focus the review. We performed systematic searches to corroborate our developing theoretical framework.

**Results:**

We present a theoretical framework based around six intervention construction principles. Theory testing provided some evidence in favour of treatment repetition, dynamic over static touch and lightening sedation. A lack of empirical evidence was identified for construction principles relating to intensity and positive/negative evaluation of emotional experience, moderate pressure touch for sedated patients and intervention delivery by relatives versus healthcare practitioners.

## 1 INTRODUCTION

Patients in intensive care units (ICUs) experience high levels of psychological and physiological stress (Boonen et al., 2013; Papathanassoglou, Giannakopoulou, Mpouzika, Bozas, & Karabinis, 2010). Causes of stress include pain (Abuatiq, Burkard, & Jo Clark, 2013), mechanical ventilation (Tate, Devito Dabbs, Hoffman, Milbrandt, & Happ, 2012), powerlessness (Yang, 2016) and experiences of social and physical disconnection (Stayt, Seers, & Tutton, 2015; Whitehorne, Gaudine, Meadus, & Solberg, 2015). Pain and distress have been linked to the development of agitation and delirium (Reade & Finfer, 2014), posttraumatic stress disorder (Morrissey & Collier, 2016) and chronic pain after ICU discharge (Papathanassoglou, 2014). High stress levels may also contribute directly towards pathophysiological sequelae through the release of neuropeptides (Papathanassoglou et al., 2010). Analgesics and sedatives are important in managing patient distress (Grounds et al., 2014). However, pharmacological side effects, such as reduced consciousness and brain dysfunction (Reade & Finfer, 2014), can prolong mechanical ventilation, thereby increasing risks of mortality and morbidity (Jackson, Proudfoot, Cann, & Walsh, 2010). In contrast, integrative therapies such as interpersonal touch may promote calm alertness and are relatively safe (Field et al., 1996; Papathanassoglou & Park, 2016).

Routine nursing and medical interventions often involve procedural touch, which patients may find unpleasant (Samuelson, 2011). In contrast, interpersonal touch interventions, such as affective touch and massage, are aimed at improving a patient's psychological state. Further, in the current context of light sedation targets for ICU patients, the role of human presence in reducing fear and anxiety is increasingly recognized as important (Baumgarten & Poulsen, 2015).

### 1.1 Background

Interpersonal touch interventions are complex because they contain multiple interacting components (Clark, 2013), including physical, physiological, psychological and interpersonal factors (Olausson, Wessberg, Morrison, & McGlone, 2016). Reviews of interpersonal touch interventions in neonatal ICU demonstrate statistically significant reductions for length of hospital stay, risk of sepsis and mortality (Álvarez et al., 2017; Conde‐Agudelo & Díaz‐Rossello, 2016). In contrast, while reviews of interpersonal touch interventions for critically ill and acutely ill adults report benefits for physiological stress indicators, pain, anxiety and sleep (Boitor, Gélinas, Richard‐Lalonde, & Thombs, 2017; Miozzo, Stein, Bozzetto, & Planz, 2016; Papathanassoglou & Mpouzika, 2012), the long‐term clinical benefits remain uncertain. Further, as is typically found for complex interventions (Parry, Carson‐Stevens, Luff, McPherson, & Goldmann, 2013), reported effects vary considerably between studies (Papathanassoglou & Mpouzika, 2012). Such variations may result from differences in intervention characteristics or study methodology. Importantly, outcomes may also vary depending on context because contextual factors can either activate or block the underlying mechanisms (Ellingsen, Leknes, Løseth, Wessberg, & Olausson, 2016). Thus, while meta‐analyses are useful for estimating aggregate effectiveness, the more important task is to understand how interventions work (Chen & Rossi, 1983).

Recent reviews (Boitor et al., 2017; Hu et al., 2015; Miozzo et al., 2016; Papathanassoglou & Mpouzika, 2012) have described studies of interpersonal touch interventions in adult ICU as being generally small‐scale randomized trials (group sizes <50), of variable methodological quality. To date, attention has focused more on massage interventions compared with acupressure, reflexology or handholding interventions. Study comparability remains problematic because of inadequate reporting for intervention characteristics such as pressure and velocity (Lindgren et al., 2013). In terms of outcome parameters, few ICU studies have investigated neuroendocrine effects and none have employed neuroimaging techniques.

The aim of this review was to understand how interpersonal touch interventions modulate stress and related outcomes in ICU patients and to develop a theoretical framework to inform the design, implementation and evaluation of interpersonal touch interventions.

## 2 DESIGN

We used realist review methodology (Pawson, 2002; Saul, Willis, Bitz, & Best, 2013; Wong, Westhorp, Pawson, & Greenhalgh, 2013) and followed RAMESES reporting standards (Wong, Greenhalgh, Westhorp, Buckingham, & Pawson, 2013). Realist review is an approach to building and testing conceptual frameworks that can inform intervention design (Fletcher et al., 2016; Pawson, Greenhalgh, Harvey, & Walshe, 2005). In contrast to outcome‐driven approaches, which conceptualize interventions as black boxes (Astbury & Leeuw, 2010), realism recognizes the complexity of interventions (Pawson, 2013). Thus, realists seek to answer all or part of the question “What works, how, why, for whom, to what extent and in what circumstances, in what respect and over what duration?” (Wong, Greenhalgh, et al., 2013, p. 1011). Unlike conventional systematic reviews, realist reviews follow a more iterative and idiosyncratic route. Thus, while the theory testing process should be systematic and transparent, creativity and judgement are largely prioritized over reproducibility and uniformity (Pawson, Greenhalgh, Harvey, & Walshe, 2004).

Additionally, we took a design‐oriented approach, using “context–intervention–mechanism–outcome logic” (CIMO‐logic) (Denyer, Tranfield, & van Aken, 2008; Pawson & Tilley, 1997) to gain an understanding of how different types of interventions might work best in different contexts. We considered that an intervention design approach was appropriate to the subject area because the paucity of empirical evidence from ICU studies suggested a need to transcend existing systems to create new “design propositions” (Romme, 2003), that is CIMO configurations (CIMOCs). Design propositions are depicted as follows: in context *C*, use intervention type *I*, to activate mechanism *M*, to achieve outcome *O*. Further, we created “construction principles” comprising interlinked sets of CIMOCs (Romme & Endenburg, 2006). Construction principles were framed as broad solutions to the problem of reducing patient stress.

In line with the sensory and social dimensions of interpersonal touch, our definition of “mechanism” was broader than definitions employed for exclusively social interventions (cf. Lacouture, Breton, Guichard, & Ridde, 2015); we defined mechanism as a generally hidden, context‐sensitive, physiological or psychological response of an individual to the intervention that leads directly or indirectly to an outcome of interest. We defined “context” as any feature distinct from the intervention per se that acts on a mechanism to influence outcomes.

### 2.1 Focusing the review

We focused the review based on: data limitations suggested by a preliminary scoping review; issues relating to intervention feasibility, acceptability and safety; and insights gained during the theory testing process. We consulted local stakeholders (four ICU nurses (colleagues of SJH) and two patient representative groups) to ensure that the focus of the review took into account the concerns of potential knowledge users. Stakeholder consultations took place by email and/or in person. Further, to ensure that relevant theories were identified and given appropriate consideration and that data interpretation was congruent with current knowledge in the field, we established correspondence with a panel of four external experts. Experts were selected on the basis of recent publications (since 2012) on interpersonal touch in critical care or complex critical care interventions (one existing contact of SJH).

While the benefits of touch extend to touch interventionists such as family members (Prichard & Newcomb, 2015), who may be well placed to deliver touch interventions (Hill, 1993), we restricted the review to patient outcomes following stakeholder opinion that relatives would be more motivated to use the intervention on the basis of evidence supporting patient benefits. We chose not to focus on intervention duration because we considered that short interventions (e.g., 10 min) would be preferable to minimize clinical interruptions (Martorella, Boitor, Michaud, & Gélinas, 2014). Additionally, while recognizing that light and moderate pressure touch activate different neurophysiological pathways (Olausson et al., 2016), because of stakeholder concerns about safety, we chose not focus on pressure because forceful massage techniques are associated more frequently with serious, although rare, adverse events (Ernst, 2003; Posadzki & Ernst, 2013). To a large extent however, the review's focus was restricted by the data limitations suggested by the scoping review. For example, we identified limited reporting of interventionists’ use of eye contact and facial expression, which may constitute influential contextual cues (Ellingsen et al., 2016; Kerr, Wiechula, Feo, Schultz, & Kitson, 2016).

## 3 METHOD

### 3.1 Search methods

We employed a two‐stage search process consisting of a broad, scoping search and a systematic search to corroborate our developing theoretical framework (Wong, Westhorp, et al., 2013). Searches were completed by SJH.

#### 3.1.1 Scoping the literature

The aims of the scoping search were to identify and critically evaluate theories explaining how the intervention might work, to assess the extent of ICU primary research and to create intervention construction principles and associated CIMOCs. Searches were performed from March 2016 to March 2018. To identify theories that may be transferable between different domains (Astbury & Leeuw, 2010), we placed no restrictions on evidence sources. Thus, we included mechanical touch interventions, animal research, theoretical papers and opinion pieces. Databases searched included CINAHL, MEDLINE, PsycINFO and Google Scholar. Searches included textwords and terms representing CIMO components. Subject experts suggested search terms and articles. Examples of search terms are provided in Supporting information Appendix S1. Additionally, we combined search terms with elements of the BeHEMoTh framework (Booth & Carroll, 2015) and employed lateral search techniques.

#### 3.1.2 Systematic search

We conducted one main search and one supplementary systematic search. An Information Specialist informed the search processes. In recognition that a range of articles may refer to studies of interest, we omitted search limiters. Examples of database search strategies are provided in Supporting information Appendix S1. We supplemented search results with results from our scoping search.

##### Main systematic search

We searched CINAHL, MEDLINE, EMBASE, CENTRAL, Web of Science, Open Grey, The Clinical Trials Register, ProQuest (Dissertations and Theses), EthOs, Google and Google Scholar in August 2016. Search strategies included terms and textwords representing the population of interest (ICU) and the intervention. Hand searches were undertaken for the previous 12 months of journal issues for Complementary Therapies in Clinical Practice, Intensive and Critical Care Nursing, Journal of Advanced Nursing and Journal of Clinical Nursing.

##### Supplementary systematic search

Due to the absence of ICU studies of sufficient data quality to address a preliminary construction principle, we conducted a supplementary systematic search in March 2017 to identify any inpatient studies of a similar nature. We searched CINAHL and MEDLINE using textwords representing the intervention and the interventionist of interest (family members).

### 3.2 Document selection

The first author (SJH) screened titles and where indicated, abstracts or full texts of documents against eligibility criteria (Supporting information Appendix S2). We included a broad range of outcomes, including pain, in recognition of the close interactions that may exist between stress and other distinct outcomes (McCracken, Zayfert, & Gross, 1992). Records for the same study were identified as “sibling papers” to create “study clusters” (Booth et al., 2013). Resources were not available for translation; therefore, we included translations only where provided. Additionally, we included English language abstracts of non‐English language sibling papers as sources of contextual information.

### 3.3 Quality appraisal

All eligible studies were appraised for quality and relevance by SJH. The Mixed Methods Appraisal Tool (MMAT; Pluye et al., 2011; Souto et al., 2015) was used to assess study quality. In accordance with realist methodology, studies were not excluded on the basis of MMAT scores; rather, case‐by‐case decisions were taken as to whether data were of sufficient quality and relevance to warrant some contribution towards theory development (Pawson, 2006). Extracted data were judged of insufficient quality if they were considered to be seriously untrustworthy.

### 3.4 Data abstraction

Data abstraction was undertaken by SJH using a data extraction form (template provided in Supporting information Appendix S3). Data extraction form design was informed by Cochrane Skin Group (2009) and Higgins and Deeks (2008) and adapted for the review following a trial using five studies. Outcome data were extracted only if reported in full‐text English language papers.

### 3.5 Analysis

Through a process of scoping and focusing with stakeholder consultation, authors (SJH, EDEP and LL) iteratively developed four preliminary testable construction principles and associated CIMOCs. To test the construction principles, four matrices were created that summarized studies in terms of: (a) study design; (b) clinical context; (c) intervention characteristics; and (d) outcome measures. The matrices were employed to select data to test our construction principles, using within‐study and between‐study comparisons. Due to high study heterogeneity, study selection criteria were developed iteratively, rather than being set in advance. Where studies compared identical interventions with and without essential oils, essential oil comparators were excluded. The selected data were interpreted by SJH, EDEP and LL. Insights gained during theory testing directed our continued search for explanatory theory and led to two further construction principles that we considered untestable within the limitations of our matrices.

### 3.6 Ethics

Patient consent and ethical approval were not required.

## 4 RESULTS

### 4.1 Search outcome

**Figure 1:**
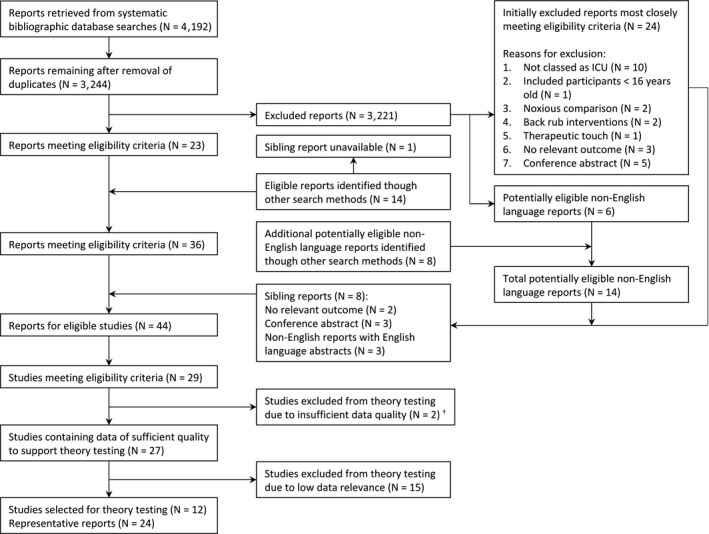
Document flow diagram for main systematic search. ^†^Excluded because of statistically improbable similarities in outcome data for non‐identical control groups

Document flow processes for our main and supplementary systematic searches are presented in Figure 1 and Supporting information Appendix S4, respectively. Additionally, we provide a summary of all studies meeting the eligibility criteria (Supporting information Appendix S5) and list excluded articles most closely meeting the eligibility criteria (Supporting information Appendix S6). Of the 13 studies we employed to test our construction principles, 12 were ICU studies, and one took place in coronary care. Eleven studies used a quantitative design (10 RCTs, one quantitative descriptive) and two used a combination of RCT and qualitative designs. Interventions comprised massage (four studies), reflexology (two), acupressure (two), massage and acupressure (two), massage and reflexology (one), wrist holding (one) and social/affective touch (one). Seven studies employed touch intervention comparators. The methodological quality of the studies was assessed as variable (Supporting information Appendix S7). No study met all MMAT criteria.

### 4.2 Theoretical Framework

**Figure 2:**
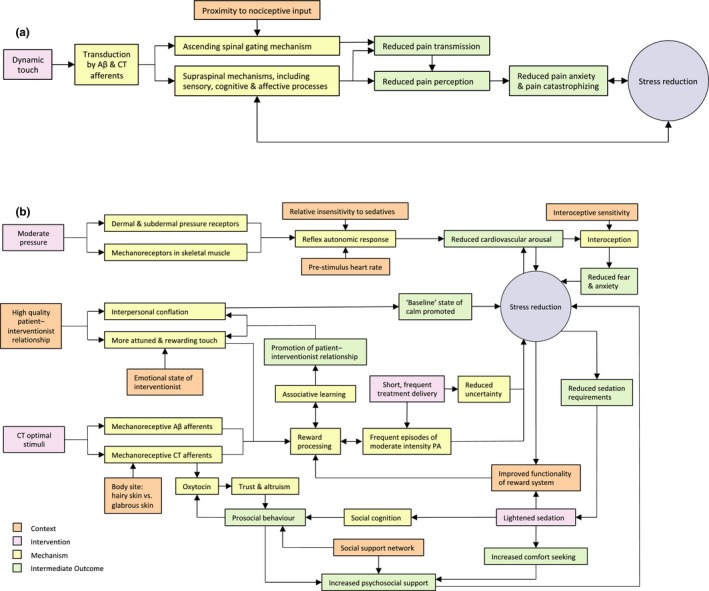
Logic models for interpersonal touch interventions in ICU. The models are based on our interpretation and synthesis of the evidence sources informing our theoretical framework. For simplicity, we consider only positive effects and do not present all context–intervention–mechanism–outcome configurations. The exclusion of mechanisms relating to negative effects does not imply any hypotheses regarding the importance of negative effects. (a) Dynamic touch. (b) The figure illustrates the direct effects of moderate pressure on the autonomic nervous system versus the more indirect effects of light pressure CT optimal touch that are more reliant on cortical processes. CT: C‐tactile afferents (present in hairy skin only); PA: positive affect. CT optimal stimuli: indentation force 0.3–2.5 mN, velocity 1–10 cm/s, warm (typical skin) temperature (Vallbo, Löken, & Wessberg, 2016).

Our theoretical framework (Table 1, Figure 2 and Supporting information Appendix S8) was based on six construction principles, each representing a set of interlinked CIMOCs (we provide examples of CIMOCs in Supporting information Appendix S9). Construction principles related to: two intervention factors (dynamic vs. static touch, i.e. touch involving motion (e.g., massage) or no motion; and moderate vs. light pressure); three contextual factors (sedation level, the patient–interventionist relationship and the patient's previous experience of the intervention); and one mechanism (subjective intensity and positive/negative evaluation of emotional experience or affect). Intervention mechanisms and emergent processes encompassed the neurophysiology of touch and pain, psychological factors and interpersonal factors.

**Table 1:** Construction principles and theoretical framework for interpersonal touch interventions

Modifying contexts	Mechanisms and key papers	Outcomes	Emergent processes
*Construction Principle 1. Dynamic touch may reduce stress and pain more effectively than static touch*			
Proximity to nociceptive input	Ascending inhibition of pain signals at the neural gate in the spinal cord. Inhibition of nociceptive transmission mediated by endogenous opioids. Habig et al. (2017), Ladak et al. (2014), Mancini et al. (2014), Mancini, Beaumont, Hu, Haggard, and Iannetti (2015), Melzack and Wall (1965), Watanabe, Piché, and Hotta (2015)	Reduced pain signalling to the brain, inhibition of somatocardiac reflexes	Positive interactions between pain perception, health‐promoting behaviours and stress regulation
Eye contact	Supraspinal mechanisms, including sensory, cognitive & affective processes, modulate pain transmission (Melzack & Wall, 1965) & subjective pain experience (Bushnell et al., 2013; Melzack, 2001; Melzack & Katz, 2013)	Reduced pain signalling, reduced perceptions of physical and psychological pain. Improved stress regulation and action programmes	
Neural activity and connectivity in the reward system	The reward system, which comprises cortical & subcortical brain regions, is activated more strongly by gentle stroking movements versus static touch. Lindgren et al.. (2012)	Increased reward processing	
Calmness of environment	Reward reduces stress reactivity via endogenous opioid release (Creswell, Pacillio, Denson, & Satyshur, 2013; Kaada & Torsteinbø, 1989). Pleasure‐related analgesia (Kut et al., 2011)	Reduced stress reactivity, improved cognitive performance, reduced pain perception	
Glabrous skin versus hairy skin Skin temperature of interventionist	CT afferents present in hairy skin respond optimally to warm, medium‐velocity, gentle stroking. The CT pathway ascends to the insula & cortical networks via the dorsal horn. Liljencrantz et al. (2017), von Mohr, Kirsch, and Fotopoulou (2017), Morrison (2016), Vallbo et al. (2016)	Increased pleasure, reduced perception of physical pain, reduced feelings of social exclusion	
	OT is released in response to CT afferent stimulation. Walker, Trotter, Swaney, Marshall, and Mcglone (2017)	Increased pleasure, reduced stress reactivity, reduced anxiety	Positive interactions between OT release and prosocial behaviours (including touch)
	OT modulates HPA‐axis activity, increases reward processing, & reduces stress reactivity, fear & anxiety. Cardoso, Kingdon, and Ellenbogen (2014), Sippel et al. (2017), Walker et al. (2017)		
Social support network	OT may promote prosocial effects including trust, emotional recognition and altruism via action on multiple stages of social decision‐making (Piva & Chang, 2018)	Prosocial behaviours, increased psychosocial support	
*Construction Principle 2. Lightening sedation may promote touch‐mediated reductions in stress*			
Sedation state	Increased cortical activity & connectivity promotes reward processing & social cognition. MacDonald et al. (2015)	Increased reward responding to social contact, increased prosocial behaviours and psychosocial support. Reduced perceptions of missing out on what could be a more pleasurable experience	Positive interactions between reduced sedation, increased reward responding, reduced stress reactivity and sedation requirements
Opioid administration, chronic pain, separation distress	Optimizing opioid administration (avoiding oversedation) may promote social comfort seeking (Loseth, Ellingsen, & Leknes, 2014) & increase pleasantness of social touch (Case et al., 2016)		
*Construction Principle 3. Touch provided by a familiar conspecific may promote stress reduction*			
Patient's perceived quality of relationship with touch interventionist	Psychosocial resources are construed as bioenergetics resources. Conflation of self & others results in a diminished perception of threat. Proximal mediators may include OT & endogenous opioids. Beckes and Coan (2011), Coan and Sbarra (2015), Coan et al. (2006)	Attenuated physiological threat response, promotion of baseline state of relative calm, reduced metabolic demands	The association of the beneficial effects of the intervention with the interventionist may promotion the dyadic relationship
Emotional state of interventionist	The communication of positive emotions, such as love and sympathy, via touch. Hertenstein, Keltner, et al. (2006), Hertenstein, Verkamp, Kerestes, and Holmes (2006)	Increased positive emotions	
Empathy of interventionist towards patient	Partners express empathy by providing more attuned & rewarding touch (Goldstein et al., 2016). Feeling understood activates the reward system (Morelli, Torre, & Eisenberger, 2014)	Increased pleasure, reduced pain	
*Construction Principle 4. Treatment repetition may provide cumulative benefits*			
Patient's previous experience of the intervention	Stress reduction improves functionality of reward‐related neural circuitry. Bogdan and Pizzagalli (2006), Pizzagalli (2014)	Normalized hedonic capacity, increased positive affect, reduced risk of depression	Positive interactions between stress reduction and reward responding
Neural activity and connectivity in the reward system	Positive neural interactions between reward components “liking,” “wanting” & “learning” including cognitive & unconscious processes. [Link]Berridge and Robinson (2003)	Increased wanting, liking and expectation of the intervention	
	Increased familiarity with the intervention may reduce stress by virtue of knowing what to expect.[Link] De Berker et al. (2016), Peters et al. (2017)	Reduced stress response, reduced cerebral energy demands	
	Lower levels of anxiety & psychological stress may reduce chronic pain perception via modulation of the neurosignature pattern. Melzack (2001)	Reduced chronic pain perception	Positive interactions between reduced psychological stress and reduced pain perception. Sharp and Harvey (2001)
	Reduced perception of pain reduces pain anxiety & pain catastrophizing. McCracken et al. (1992), Sullivan, Bishop, and Pivik (1995)	Reduced anxiety	
*Construction Principle 5. Interventions that provide frequent episodes of moderate intensity PA may provide greater long‐term stress reduction compared with interventions that provide infrequent episodes of high‐intensity PA*			
Level of perceived psychological stress Morbidity status	Promotion of optimal affect variability (Diener, Colvin, et al., 1991; Human et al., 2015; Parducci, 1968, 1984 ; Pressman & Cohen, 2005; Solomon, 1980) & higher relative frequency of PA versus NA (Blevins et al., 2017; Diener, Sandvik, et al., 1991)	Reduced stress response, improved stress resilience and recovery. Positive effects on social support and health behaviours	
Cognitive functioning Chronic stress	Frequent treatment repetition facilitates estimates of predictability, which reduces cognitive demands & stress response.[Link]De Berker et al. (2016), Peters et al. (2017)	Reduced stress response, reduced cerebral energy demands	
Anhedonia, depression	Promotion of anticipatory pleasure. Gard, Gard, Kring, and John (2006), Gooding and Pflum (2014)	Increased frequency of anticipatory pleasure	
*Construction Principle 6. Moderate pressure touch may reduce stress more directly compared with light pressure touch*			
Autonomic effects may be relatively insensitive to sedation	Moderate pressure stimulates dermal & subdermal pressure receptors activating structures in the autonomic nervous system. Field (2016b), Field, Diego, and Hernandez‐Reif (2010)	Reduced heart rate and blood pressure, reduced cortisol levels	Positive feedback between reduced cardiovascular arousal and reduced negative emotions
Pre‐stimulus heart rate	Mechanical pressure stimulation of mechanoreceptors in skeletal muscle elicits a reflexive autonomic nervous system response. Watanabe and Hotta (2017)	Modulation of heart rate and blood pressure	
Interoceptive sensitivity	Arterial baroreceptors transmit information about cardiovascular arousal to brain regions implicated in affective & cognitive processing. Garfinkel and Critchley (2016)	Reduced fear and anxiety, generally enhanced perception and cognition	

Note

OT: oxytocin; HPA: hypothalamic–pituitary–adrenal; PA: positive affect; NA: negative affect. Linkages between context–intervention–mechanism–outcome components occur both within and across construction principles. For simplicity, we consider only positive effects. The exclusion of mechanisms relating to negative effects does not imply any hypotheses regarding the importance of negative effects.

Mechanisms may also be applicable to repetition of massage strokes.

### 4.3 Evidence for intervention Construction Principles

The following sections present evidence for construction principles 1–4 (Table 1) using data from studies identified in our systematic search. Additionally, where possible, we consider evidence relating to interactions between specific CIMO components. However, a consideration of all details covered by our theoretical framework was beyond the scope of this review.

#### 4.3.1 Construction Principle 1: Dynamic touch may reduce stress and pain more effectively than static touch

Two studies investigated the effects of dynamic touch versus predominantly static touch (Table 2). As can be seen from Table 2, reported results for these studies were entirely (Tsay, Wang, Lin, & Chung, 2005) or predominantly (Boitor, Martorella, Arbour, Michaud, & Gélinas, 2015; Martorella et al., 2014) in favour of dynamic touch, thus supporting our hypothesis.

**Table 2:** Comparison of quantitative and qualitative results for groups receiving dynamic touch versus predominantly static touch

Study design, mean group size, key records, country	Context	Intervention	Results	
			Results favour dynamic or static touch	Results similar
RCT & qualitative (descriptive), *N = *20 Boitor et al. (2015), Martorella et al. (2014) Canada	Thoracic pain following cardiac surgery Morphine received before each intervention	*Dynamic touch* Application of lavender hand cream plus hand massage 15 min, 3 times a day x 1	*Quantitative:* Lower pain behaviour scores after 1st intervention[Link] Reduced pain intensity after 2nd (*p* = 0.088) & 3rd intervention[Link] Lower muscle tension after 3rd intervention (*p* = 0.079) *Qualitative:* analgesic effects more strongly highlighted	*Quantitative* [Link] *:* Global pain experience after transfer from ICU, RR, SBP, SpO_2_ *Qualitative*: Pleasantness of intervention not reported to differ between groups
		*Predominantly static touch* Application of lavender hand cream plus handholding 15 min, 3 times a day x 1	*Quantitative:* Decreased DBP after 2nd intervention[Link] Lower HR for 3rd intervention[Link] *Qualitative:* Positive appraisal of human touch and presence more strongly highlighted	
RCT, N = 26 Tsay et al. (2005) Taiwan, China	100% ventilated 95% tracheostomies Diagnosed with COPD Alert, not receiving tranquilizers	*Dynamic touch* Massage (shoulder and arms, 3 min) and acupressure (hands, ears, wrists, 12 min) 15 min, once daily x 10	*Quantitative*: lower RR[Link], lower perceived dyspnoea[Link], lower perceived anxiety[Link], & lower HR (*p* = 0.05)	
		*Predominantly static touch* Massage and handholding Once daily x 10		

Note

RR: respiratory rate; HR: heart rate; DBP: diastolic blood pressure; SBP: systolic blood pressure; SpO_2_: peripheral oxygen saturation.

*p* <0.05

*p* <0.01.

*p* ≥ 0.1.

In considering the mechanisms underlying the effects of dynamic touch, we now focus on quantitative and qualitative data reported by Boitor et al. (2015) and Martorella et al. (2014) in their study of hand massage versus handholding for postoperative cardiac surgery patients. To explain the potential analgesic effects of hand massage, Boitor and colleagues refer to the mechanism of ascending inhibition of nociceptive signalling in the spinal cord via the stimulation of large‐diameter Aβ mechanoreceptive afferents (Melzack & Wall, 1965). An important condition, however, for activation of the ascending spinal gating mechanism, is that a connection must exist between the spinal nerve transmitting the nociceptive input and the spinal nerve transmitting the tactile input. Furthermore, contrary to R. Melzack, personal communication, November 20, 2012, cited in Hogan et al. (2014), recent work by Mancini, Nash, Iannetti, and Haggard (2014) suggests that within the sensory territory innervated by the relevant spinal nerve, the proximity of tactile input to the site of injury is an important factor. Thus, activation of the ascending spinal gating mechanism in Boitor et al.’s study is likely to have been limited because the massage was not directed towards the patients’ painful thoracic surgical site. Additionally, mechanism activation is likely to have been restricted further by the limited overlap between dermatomes stimulated by the hand massage (C6–C8 and T1) and dermatomes proximal to the patients’ sternal incision (e.g., C4 and T2–T8) (Ladak, Tubbs, & Spinner, 2014; Lee, McPhee, & Stringer, 2008). We are, however, unable to fully exclude this mechanism because of the possibility of interneural communication between peripheral nerve territories (Ladak et al., 2014), variation in specific nerve territories between individuals and major discrepancies between dermatome maps (Downs & Laporte, 2011). Interestingly, some study participants stated they would have preferred the massage had targeted areas they identified as painful (Martorella et al.), which would then have activated the ascending inhibitory pathway.

As an alternative to the ascending spinal gating mechanism described above, we suggest that supraspinal mechanisms (Figure 2a) offer a more likely explanation for the superior analgesic effects reported for the hand massage group. Further, the finding that pleasure was not more strongly highlighted in the hand massage group suggests that the superior analgesic effects reported for this group may not have resulted from the pleasure of the massage (Kut et al., 2011), but rather from the sensory and cognitive effects of the massage (Bushnell, Čeko, & Low, 2013; Melzack & Katz, 2013). By redirecting attention away from the pain, the visual and tactile sensory inputs of the massage may have provided more effective analgesia than the similarly pleasant but less distracting experience of handholding. Finally, it is uncertain why human touch and presence were more strongly highlighted in the handholding group. One possible explanation is that the technical demands of massage delivery may have restricted the interventionist's ability to engage in direct eye contact, which may promote feelings of trust and positive attitudinal shift (Kerr et al., 2016).

#### 4.3.2 Construction Principle 2: Lightening sedation may promote touch‐mediated reductions in stress

To investigate the effect of lightening sedation on the effects of touch, we compared outcomes from two studies specifying that patients received sedatives with six studies where sedation was specified as restricted (Table 3). Both sedation condition studies investigated non‐coma patients who predominantly received mechanical ventilation; we therefore restricted our comparison to studies investigating similar populations. The pattern of outcomes obtained from the between‐study comparison (Table 3) suggests that restricting sedation administration may promote the effectiveness of the intervention. This interpretation, however, remains tentative due to the small number of studies employing a sedation condition, differences in outcome measures employed and potentially confounding variables such as intervention design and the degree of pressure employed. Both sedation condition studies employed gentle massage techniques, whereas the majority of restricted sedation studies demonstrating stronger evidence for effects used greater pressure in the form of acupressure or reflexology, which may elicit more direct autonomic effects (Field, 2016b; Watanabe & Hotta, 2017; Section 4.4).

**Table 3:** Summary of quantitative evidence for touch interventions in sedated patients versus patients for whom sedation was restricted

Study design, mean group size, key records, country	Patient context	Touch intervention	Strength of evidence in favour of intervention group		
			Weak or absent[Link]	Intermediate[Link]	Stronger[Link]
**Sedated**					
Two‐arm RCT, N = 22 Henricson (2008), Henricson, Berglund, et al. (2008), Henricson, Ersson, Määttä, Segesten, and Berglund (2008) Sweden	84% ventilated, minimally responsive to restless No changes to sedatives during procedure	Tactile touch (slow stroking, soft/firm) to hands, feet, stomach, head, face, chest, arms, legs. Music 60 min, once daily ×5	HR, SBP, alertness, blood glucose, blood oxytocin. Insulin, noradrenaline and sedation requirements	Anxiety, DBP	
Three‐arm RCT, N = 35 Olleveant (2003) UK	80% ventilated, level of anaesthesia: non to minimal, 73%; moderate to high, 27%	Leg massage (light and gentle) with almond oil 14–20 min repeated once after three days	HR, RR, SBP, DBP, pain, analgesia & sedation requirements, sedation scores, anxiety, depression, quality of life, ICU survival time and length of ICU stay		
**Sedation restricted**					
Two‐arm RCT, *N = *30 Korhan, Khorshid, and Uyar (2014) Turkey	100% ventilated, GCS ≥9 Sedation (propofol) stopped 30 min prior to intervention	Reflexology to hands, feet & ears 30 min, twice a day ×5	Consciousness component of the AACNSAS		HR, RR, SBP, DBP Average score for agitation, anxiety, sleep and ventilator synchrony components of the AACNSAS
Two‐arm RCT, *N = *26 Tsay et al. (2005) Taiwan, China	100% ventilated, 96% tracheostomies, alert, diagnosed with COPD Patients receiving tranquilizers were excluded	Massage (3 min; shoulder and arms) and acupressure points (12 min; hands, ears, wrists) 15 min, once daily ×10		HR	RR, anxiety, dyspnoea
Two‐arm RCT, N = 35 Çınar (2008), Çinar Yücel and Eşer (2015) Turkey	100% ventilated, diagnosed with COPD, GCS 9–15 No sedatives during intervention	Hand massage (10 min) and hand acupressure (8 min) 18 min, once daily ×5	SBP, SpO_2_	HR, RR, DBP, dyspnoea	Anxiety
Two‐arm RCT, N = 32 Yousefi, Naderi, and Daryabeigi (2015), Yousefi, Naderi, Daryabeigi, and Tajmiri (2015) Iran	63% ventilated, GCS 9–12 Sedatives and narcotics (if required) taken>6 hr prior to sampling	Family interventionist, handholding, touching of head and face and positive verbal support 17 min, twice a day ×1		NA	HR, SBP, DBP, SpO_2_
Two‐arm cross‐over RCT, N = 35. Souri Lakie, Bolhasani, Nobahar, Fakhr Movahedi, and Mahmoudi (2012) Iran	100% ventilated, agitated, GCS ≥7 Considered clear of sedatives	Wrist holding without pressure 5 min ×1		NA	SpO_2_
Three‐arm RCT, N = 31 Ebadi, Kavei, Moradian, and Saeid (2015) Iran	100% ventilated (weaning), conscious, postoperative (elective cardiac surgery) No sedatives pre‐intervention	Foot reflexology massage 20 min × 1	HR, RR, SBP, DBP, mean BP, SpO_2_	NA	Mechanical ventilation weaning time
		Surface touch to heels, without pressure but involved movement[Link] 20 min × 1	HR, RR, SBP, DBP, mean BP, SpO_2_, mechanical ventilation weaning time	NA	

Note

RR: respiratory rate; HR: heart rate; DBP: diastolic blood pressure; SBP: systolic blood pressure; SpO_2_: peripheral oxygen saturation; AACNSAS: American Association of Critical‐Care Nurses Sedation Assessment Scale. NA: not applicable because single treatment employed or time series data not reported.

^b,c^Percentages are based on results reported for each treatment, with the exception of Korhan et al. (2014), Yousefi, Naderi, and Daryabeigi (2015) and Yousefi, Naderi, Daryabeigi, and Tajmiri (2015) who report mean results for twice daily treatments.

Differences between intervention and control groups either not reported or not statistically significant (*p*>0.05).

In favour of the intervention group for ≤70% of treatments (*p* <0.05).

In favour of the intervention group either overall or for >70% of treatments (*p* <0.05).

A. Ebadi, personal communication, June 8, 2017.

In terms of evidence for the proposed underlying mechanisms, qualitative findings by Henricson Segesten Berglund and Määttä (2009) that sedated patients wished to be more alert to more fully enjoy the pleasure of touch supported our proposition that sedation inhibits reward processing. Further, Henricson et al. reported that the participants’ awareness of missing out on a more pleasurable experience led to feelings of sadness. For example, one man recounted sadly:

“[…] it is a pity that I was not more alert during the touch […] it should have been a really good experience […] you experience pleasurable things the same time as you are sleepy…” (Henricson et al. 2009, p. 328)

#### 4.3.3 Construction Principle 3: Touch provided by a familiar conspecific may promote stress reduction

One study compared touch delivered by a companion vs. touch provided by a stranger (Adib‐Hajbaghery, Rajabi‐Beheshtabad, & Ardjmand, 2015). This study, a three‐arm RCT conducted on 90 male patients in a coronary care setting in Iran, compared the effects of a single 60‐min whole‐body massage delivered by a patient's companion vs. a nurse/researcher qualified in massage therapy. The study reported post‐treatment decreases in the patients’ median blood cortisol levels for both treatment groups, but no significant between‐group differences in cortisol levels. The authors also reported a greater reduction in median cortisol level for the companion group compared with the nurse group (92 vs. 85 nanomoles). However, the reduction was statistically significant only for the nurse group. Additionally, patients in the nurse group reported higher ratings for “satisfaction of massage.”

A potentially confounding variable for this study was the companions’ relative lack of massage training and likely technical ability, particularly since the intervention was long and complicated and was performed only once. Additionally, the discrepancy in the numbers of interventionists per group (one nurse vs. 30 companions) may have contributed to the higher post‐treatment variability in cortisol levels for the companion group.

In terms of potential underlying mechanisms, given that the beneficial effects of touch may depend on relationship quality (Coan, Schaefer, & Davidson, 2006), it is possible that the interpersonal context employed by Adib‐Hajbaghery et al. (2015) (sons, brothers and same‐sex friends) may have activated the mechanisms of interpersonal conflation relatively less strongly compared with that which may have been achieved in the context of a high quality spousal relationship. Additionally, as suggested by Goldstein, Shamay‐Tsoory, Yellinek, and Weissman‐Fogel (2016), empathy between partners may facilitate more attuned and rewarding touch, as may also dyadic touch familiarity. Thus, in Adib‐Hajbaghery et al.’s study, the companion group's potentially relatively limited levels of empathy and familiarity with touching the participant may have restricted their ability to provide rewarding touch. Finally, given that a range of emotions may be communicated by touch (Hertenstein, Keltner, App, Bulleit, & Jaskolka, 2006), the communication of negative emotions such as fear, resulting from the companions’ potential performance anxiety, may have impeded their ability to communicate positive emotions. In summary, the evidence did not support our construction principle. However, we speculate that the selection of companions and the nature of the intervention may have limited activation of the proposed underlying mechanisms.

#### 4.3.4 Construction Principle 4: Treatment repetition may provide cumulative benefits

To investigate the effects of treatment repetition, we present quantitative evidence from 11 study groups (Table 4). As can be seen from Table 4, groups demonstrating “stronger” evidence for time‐dependent effects received at least five treatments. Further, for some groups, intervention benefits appeared to be delayed, with statistically significant effects apparent only after multiple treatments, for example Boitor et al. (2015), Çınar Yücel and Eşer (2015) and Tsay et al. (2005). Additionally, groups demonstrating stronger evidence for time‐dependent effects received dynamic touch, restricted sedation and moderate pressure (reflexology or acupressure; Sections 4.3.1, 4.3.2 and 4.4).

**Table 4:** Summary of quantitative evidence supporting treatment repetition effects for interpersonal touch interventions

Study design, mean group size, key records, country	Patient context	Intervention	Strength of evidence for treatment repetition effects			
			Absent or negligible	Weaker[Link]	Intermediate[Link]	Stronger[Link]
RCT, N = 30 Korhan et al. (2014) Turkey	100% ventilated GCS ≥9 Restricted sedation	Reflexology to hands, feet and ears 30 min, twice a day × 5	Consciousness component of the AACNSAS	Anxiety, agitation, ventilator synchrony and sleep components of the AACNSAS		RR, HR, SBP, DBP
RCT, N = 26 Tsay et al. (2005) Taiwan, China	100% prolonged mechanical ventilation 95% tracheostomies Diagnosed with COPD Alert, not receiving tranquilizers	Massage (shoulder and arms) and acupressure points (hands, ears, wrists) 15 min, once daily × 10			HR	RR, anxiety, dyspnoea
		Massage and handholding Once daily × 10	RR, HR, anxiety, dyspnoea			
RCT, N = 35 Çınar (2008), Çınar Yücel and Eşer (2015) Turkey	100% ventilated Diagnosed with COPD GCS 9–15 No sedatives during intervention	Hand massage (10 min) and hand acupressure (8 min) 18 min, once daily × 5		SBP, SpO_2_	HR, RR, DBP, dyspnoea	Anxiety
RCT, N = 22 Henricson (2008), Henricson, Berglund, et al. (2008), Henricson, Ersson, et al. (2008) Sweden	84% ventilated Minimally responsive to restless No changes to sedatives during procedure	Tactile touch and music Hands, feet, stomach, head, face, chest, arms, legs 60 min, once daily × 5	HR, SBP, anxiety, sedation requirements, blood glucose, insulin requirements	Blood oxytocin relative stability, increased alertness, vasopressor requirements	DBP	
RCT, N = 20 Boitor et al. (2015) Canada	Postoperative cardiac surgery Morphine received before each intervention	Lavender cream hand massage 15 min, 3 times a day × 1	RR, HR, BP, SpO_2_, pain behaviours	Muscle tension	Pain intensity	
		Lavender hand cream application plus handholding 15 min, 3 times a day × 1	RR, BP, SpO_2,_ pain intensity and behaviours, muscle tension		HR	
Quantitative descriptive, N = 60 Kaur, Kaur, and Bhardwaj (2012) India	53% ventilated 52% conscious, 13% semiconscious and 35% unconscious	Foot massage and reflexology Unspecified duration Twice a day × 3	HR, SBP, DBP, SpO_2_		NA	NA
RCT, N = 31 Maa et al. (2013) Taiwan, China	100% ventilated Coma patients No sedatives or opioids	Acupressure to shoulders, wrists, hands, below knees 10 min, once daily × 2	HR, RR, BP, SpO_2_, ventilation parameters			
RCT, N = 35 Olleveant (2003) UK	80% ventilated Level of anaesthesia: non to minimal, 73%; moderate to high, 27%	Leg massage with almond oil 14–20 min, repeated once after 3 days	HR, RR, SBP, DBP			
RCT, N = 30 Bagheri‐Nesami, Gorji, Rezaie, Pouresmail, and Cherati (2015) Iran	Moderate sleep disorder patients Orientated No drugs <5–6 hr before sleeping	Acupressure or sham acupressure. Head, face, ears, wrists, feet 18 min, once daily × 3	Sleep quantity, sleep quality			

Notes

AACNSAS: American Association of Critical‐Care Nurses Sedation Assessment Scale; RR: respiratory rate; HR: heart rate; SBP: systolic blood pressure; DBP: diastolic blood pressure; NA: not applicable because no comparator group.

^b,c^Percentages are based on results for each treatment, with the exception of Korhan et al. (2014) who reported mean results for twice daily treatments.

Repetition effects suggested, with differences between intervention and comparator groups (where used) either not reported or not statistically significant (*p *> 0.05).

Repetition effects suggested, supported by statistically significant differences (*p *< 0.05) between intervention and comparator groups for ≤70% of treatments.

Repetition effects supported by statistically significant differences (*p *< 0.05) between intervention and comparator groups for>70% of treatments.

In terms of mechanisms underlying the effects of treatment repetition, support for the role of oxytocin (OT) is suggested by Henricson, Berglund, Määttä, Ekman, and Segesten (2008). Henricson et al. reported that while no between‐group differences were found for OT, over the six‐day study period, OT levels in the control group showed a statistically significant (*p *= 0.01) decline, whereas in the intervention group OT remained stable. However, baseline mean OT levels were higher for the control group than for the intervention group (39 vs. 26 pM). It is therefore unclear to what extent the declining OT in the control group may be explained by regression to the mean.

We now focus on the proposed mechanism of pleasure, using qualitative findings from two contrasting studies: Henricson et al. (2009) and Martorella et al. (2014). Henricson and colleagues used a phenomenological hermeneutic method to investigate experiences of receiving an elaborate intervention that consisted of five daily 60‐min sessions of “tactile touch” to multiple body sites, in a relatively quiet and uninterrupted clinical environment. In contrast, Martorella and colleagues used a descriptive qualitative design to investigate experiences of receiving three 15‐min hand massages delivered over 24 hr in an environment that was subject to noise and interruptions.

Differences in findings between these two studies suggest that participants in the tactile touch study experienced more intense feeling of positive affect (PA) compared with participants in the hand massage study. For example, “…it was only the touch and nothing else… everything else disappeared…” (Participant One, Henricson et al., 2009, p. 328); in contrast, the quotes presented by Martorella et al. (2014) suggest that experiences of positive affect may have been less intense. Additionally, contrasting findings were apparent for participants’ reported experiences of negative affect (NA). While Martorella et al. identified only “ambivalence,” Henricson et al. (2009) identified the themes “being left without comforting touch,” representing experiences of loneliness and abandonment when the intervention ended and “being exposed to an annoying environment” reflecting the return to unpleasant normality when the treatment was over.

While the contrasting qualitative findings between Henricson et al. (2009) and Martorella et al. (2014) may be accounted for by numerous factors, including cultural differences, which may influence emotional processing and expression (Hofstede, 2011), we tentatively suggest that the more intense levels of PA reported by Henricson et al. may, at least in part, be explained by the more pleasurable nature of the intervention, promoted by the longer, more elaborate and more numerous treatments, as well as the calmer environment (Table 1). Further, we suggest that experiences of NA reported by Henricson et al. may have resulted from the relatively infrequent intense PA elicited by the intervention.

Theoretical and empirical evidence suggests that infrequent intense PA may incur emotional costs to ICU patients for several reasons (Diener, Colvin, Pavot, & Allman, 1991; Diener, Sandvik, & Pavot, 1991). Firstly, as identified by Henricson et al. (2009), affective contrast may cause an unpleasant affective state on returning to normality; secondly, since infrequent events are likely to produce less hedonic habituation, extremes of emotion are likely to persist (Solomon, 1980); and thirdly, since the value of an event depends on comparisons with other events (Parducci, 1968, 1984 ), an ICU patient's negative situation may enhance both the pleasure and associated psychological costs of intense PA. Additionally, high PA states can trigger short‐term increases in physiological arousal that may be potentially harmful, particularly in individuals at risk of acute health events (Pressman & Cohen, 2005).

Pressman and Cohen (2005) suggest that moderate PA may protect against the pathogenic effects of stress, while NA and high‐intensity PA may be detrimental to health. Also, recent work by Blevins, Sagui, and Bennett (2017) suggests that high average frequency of PA may be particularly beneficial to individuals experiencing high perceived psychological stress. While the evidence presented by Pressman and Cohen and Belvins et al. relates to chronic disease conditions and the translatability of these findings to critical illness remains uncertain, we are tempted to speculate that ICU patients, particularly those suffering from underlying chronic conditions, may gain greater benefits from interventions eliciting shorter, more frequent episodes of moderate intensity PA, rather than longer, infrequent episodes of high‐intensity PA (Table 1; Construction Principle 5), which could promote less favourable cortisol profiles (Human et al., 2015) and may result in less positive psychological and health‐related outcomes. Additionally, given that ICU patients experience high levels of uncertainty (Egerod et al., 2015) and subjective uncertainty is a defining characteristic of stress (Peters, McEwen, & Friston, 2017), the provision of frequent positive events might reduce stress by virtue of reducing environmental uncertainty. Moreover, more frequent and predicable interventions might promote anticipatory pleasure.

Finally, it is possible that while moderate PA mitigates against the distress associated with high‐intensity PA, it may elicit a more positive, adaptive stress response (Selye, 1974) than low‐intensity PA. Thus, as suggested by Pressman and Cohen (2005), moderate PA may provide greater long‐term health benefits than low‐ or high‐intensity PA. We therefore speculate that a polynomial relationship may exist between the immediate effects of treatment on PA and the long‐term effects of multiple treatments.

### 4.4 Additional Insights

In the light of evidence that cortical processing may be a key mechanism underlying the benefits of gentle, medium‐velocity touch (Table 1, Figure 2b), gentle touch may have limited effectiveness for sedated patients due to reduced corticocortical and subcorticocortical connectivity (MacDonald, Naci, MacDonald, & Owen, 2015). In contrast, moderate pressure touch is suggested to elicit a relaxation response by increasing parasympathetic activity and/or reducing sympathetic activity (Field, 2016b; Watanabe & Hotta, 2017). The mechanisms underlying the effects of moderate pressure touch may therefore be less reliant on cortical processes (Table 1; Construction Principle 6). Further, although we are unaware of studies investigating the effect of sedation on autonomic responses to moderate pressure touch, we note with interest that Kang et al. (2017) found sedation level had no significant effect on autonomic responses to noxious cutaneous stimuli. Thus, while it appears likely that interoceptive response to changes in cardiovascular arousal (Garfinkel & Critchley, 2016) may be attenuated by sedation, we propose that the powerful, direct effects of moderate pressure touch on the autonomic nervous system may provide sedated patients with greater benefits than light pressure touch.

## 5 DISCUSSION

To our knowledge, this is the first realist inquiry into interpersonal touch interventions in ICU. Unsurprisingly, given that the current state of knowledge remains in its infancy, empirical evidence for our construction principles was weak (Principles 1, 2 and 4), unsupportive (Principle 3) or unavailable (Principles 5 and 6). Furthermore, we were unable to link outcomes to specific mechanisms. Nevertheless, we believe our review has produced insights into how interpersonal touch interventions might work in the ICU context. These insights would not have been possible within the confines of a traditional outcome‐driven systematic review.

We found some evidence that for dynamic touch, mechanisms other than pleasure, such as distraction, may be more important in achieving supraspinal pain inhibition. We also highlighted the importance of considering the proximity of tactile input to nociceptive input in activating the ascending inhibitory pathway proposed by the gate control theory of pain. We found some evidence that sedation inhibits the effects of touch. We found weak evidence supporting the role of OT in treatment repetition. By comparing qualitative findings of two contrasting studies, we gained insights into the potential emotional costs patients might incur from infrequent episodes of high‐intensity PA. Further, we speculated that a polynomial relationship might exist between the immediate treatment effects and the long‐term effects of multiple treatments. Finally, we hypothesized that sedated patients might benefit preferentially from moderate rather than light pressure touch.

In conjunction with our intervention design approach, CIMO‐logic provided a useful, albeit circuitous route to theory development; having initially chosen not to focus on touch pressure because of stakeholder concerns about safety, later insights led us to re‐appraise the potential benefits of moderate pressure touch in the context of sedation. Additionally, our broad definition of mechanism usefully enabled us to envision reality acting across multiple levels, from biophysical to social (Bhaskar, 1986).

One of the main strengths of this review is that our theoretical framework is built on relevant research evidence (Fildes, 1985), as well as transferable mid‐range theories and neurophysiological mechanisms (cf. McConnell & Porter, 2016). Consultation with stakeholders ensured that the concerns of potential knowledge users were influential in focusing the review. Additionally, we attempted to minimize publication bias by employing a wide systematic search strategy, which encompassed grey literature.

We acknowledge that this review has presented only a partial description of how interpersonal touch interventions might work in an ICU setting. For example, we did not consider structural factors (McConnell & Porter, 2016) or benefits to touch interventionists (Prichard & Newcomb, 2015; Wilson, Gettel, Walsh, & Esquenazi, 2016). Moreover, by focusing on the positive effects of interpersonal touch, we have elided potentially important negative effects. For example, touch may exacerbate the symptoms of patients who have experienced trauma or abuse (Benjamin & Sohnen‐Moe, 2014; Phelan, 2009), light touch may elicit a sympathetic nervous system (i.e. pro‐stress) response (Diego & Field, 2009) and, in certain contexts, OT can elicit antisocial rather than prosocial effects (Piva & Chang, 2018). Also, there may be alternative physiological or psychological explanations for the positive effects we investigated. For example, Moyer, Rounds, and Hannum (2004) suggest that the delayed analgesic effects of multidose massage interventions may result indirectly via the facilitation of deep sleep, which is proposed to inhibit release of the pain promoting peptide, substance P (Field, 2016a). However, given the differences between analgosedation and normal sleep (Delaney, Van Haren, & Lopez, 2015), this effect may be less important in an ICU context. In considering our systematic review, this was restricted by the small number of ICU studies, high study heterogeneity, limited reporting of contextual and intervention characteristics, uncertain validity of surrogate outcome measures (Everly & Lating, 2013) and variable study quality. Few studies used qualitative designs and none used mixed method designs. Rather, most studies were RCTs that included a standard care control condition, which may have biased results due to the control groups’ potential disappointment at their allocation status (Lindström, Sundberg‐Petersson, Adami, & Tönnesen, 2010; Stevensen, 1994). Due to resource constraints, a single individual completed the search and quality appraisal processes, which may have increased the risk of bias. Generalizability of results may be limited by the historical context where the two sedation condition studies were conducted (pre‐2007); given recent trends towards lighter sedation targets (Shehabi, Bellomo, Mehta, Riker, & Takala, 2013), study participants may have been over‐sedated relative to current sedation practices.

## 6 CONCLUSION

Interpersonal touch interventions have the potential to reduce psychological and physiological stress in ICU patients. This review provides insights into how interpersonal touch interventions may more likely achieve their aims if intervention designs are informed by an understanding of the underlying generative mechanisms and the key contextual factors that activate those mechanisms. Moreover, we have described how specific types of touch interventions may be more effective in specific contexts. We have also highlighted the potential complexities of temporal effects associated with treatment repetition by identified that polynomial relationships might exist between short‐term and long‐term outcomes. While substantial gaps in the ICU literature limited our ability to fully evaluate our theoretical framework, we have outlined novel construction principles and design propositions that can be tested and refined in future studies. In addition, our theoretical framework provides guidance for nurses and other members of the multidisciplinary team wishing to support the use of interpersonal touch in practice.

## CONFLICT OF INTEREST

No conflict of interest has been declared by the authors.

## AUTHOR CONTRIBUTIONS

SJH: made substantial contributions to conception and design, acquisition of data, and analysis and interpretation of data; drafted the manuscript and revised it critically for important intellectual content. EDEP and LL: made substantial contributions to the interpretation of data; critically revised the manuscript for important intellectual content. MG and SMH: made substantial contributions to the design of the work; critically revised the manuscript for important intellectual content. AH: made substantial contributions to the conception and design of the work; provided project supervision to SJH.
