# Supplementary material for: Interpersonal touch interventions for patients in intensive care: A design‐oriented realist review
Source: Nurs Open. 2018 Oct 24;6(2):216–35. doi: 10.1002/nop2.200 (PMC6419112; doi:10.1002/nop2.200)
Supplement: Supplementary file 1 [file NOP2-6-216-s001.docx]

**Appendix S1: Example search terms and strategies**

**Scoping search**

Databases and search engines: CINAHL, MEDLINE, PsycINFO, Google, and Google Scholar.

Search dates: March 2016 to March 2018.

Examples search terms for context-intervention-mechanism-outcome (CIMO) components employed in the scoping search:

| Context | Intervention itself | Mechanism | Outcome |
| --- | --- | --- | --- |
| Ventilat* | Touch | Oxytocin | Stress |
| ICU | Massage | Cortisol | Anxiety |
| Intensive Care | Tactile | C-tactile | Pain |
| Critical Care | Sensory | Reward system | Agitation |
| Critically ill | Non-pharmacological | Pleasure | Heart rate |
| Surgical | Deep pressure touch | Relaxation response | Heart rate variability |
| Emergency Care | Accupressure | Distraction | Response |
| Anaesthetised | Reflexology | Vagus | GCS |
| Rat | Aromatherapy | Neuropeptide | Physiological |
| Infant | Hand-holding | Endorphin* | Respiratory rate |
| Allostatic load | Mechanical | Dermatome | Stress indicator |
| Sedated | Kangaroo care | Positive affect | Sleep |
| Neonatal | Effleurage | Negative affect | Blood pressure |
| Partner* | Moderate pressure | Endogenous opioid* | Psychological |
| Famil* | Interpersonal | Attachment | fMRI |

*Note.* We employed search terms individually, in combination with each other, and in combination with elements of the BeHEMoTh framework (Booth & Carroll, 2015).

**Main systematic search**

Database: EMBASE

Platform: Ovid

Dates of coverage: 1974 to August 2016

Limiters: None

Search date: 5^th^ August 2016

| **#** | **Query** | **Results** |
| --- | --- | --- |
| S1 | (coma* OR intensive care OR mechanically ventilated OR mechanical ventilation OR critical care OR critically ill).ti,ab,kw. | 301,430 |
| S2 | (intensive care OR intensive care unit OR artificial ventilation).hw. | 292,520 |
| S3 | (tactile OR massage OR reflexology OR acupressure OR touch OR sensory stimulation).ti,ab,kw. | 52,441 |
| S4 | (touch OR massage OR whole body massage OR acupressure).hw. | 28,142 |
| S5 | S1 OR S2 | 411,918 |
| S6 | S3 OR S4 | 65,361 |
| S7 | S5 AND S6 | 2007 |

**Supplementary systematic search**

Database: MEDLINE

Platform: Ovid

Dates of coverage: 1946–2017

Limiters: Non

Search date: 18^th^ March 2017

| **#** | **Query** | **Results** |
| --- | --- | --- |
| S1 | (famil* or companion* or partner* or relative* or spous* or wife* or husband*).ti. or (famil* or companion* or partner* or relative* or spous* or wife* or husband*).ab. | 2,155,248 |
| S2 | (tactile or massage or reflexology or acupressure or touch or sensory stimulation or hand-holding).ti. or (tactile or massage or reflexology or acupressure or touch or sensory stimulation or hand-holding).ab. | 42,821 |
| S3 | S1 and S2 | 4428 |

**References**

Booth, A., & Carroll, C. (2015). Systematic searching for theory to inform systematic reviews: is it feasible? Is it desirable? *Health Information and Libraries Journal*, *32*, 220–235. doi:10.1111/hir.12108
