# Supplementary material for: Interpersonal touch interventions for patients in intensive care: A design‐oriented realist review
Source: Nurs Open. 2018 Oct 24;6(2):216–35. doi: 10.1002/nop2.200 (PMC6419112; doi:10.1002/nop2.200)
Supplement: Supplementary file 2 [file NOP2-6-216-s002.docx]

**Appendix S2: Eligibility criteria for main and**

**supplementary systematic searches**

**Eligibility criteria for main systematic search**

**Population receiving intervention.**

Include*:* patients located on an intensive care unit or a clinical unit possessing ventilation capacity.

Exclude: samples containing patients < 16 years old, patients located on a coronary care unit (unless ventilation capacity specified).

**Intervention.**

Include: massage, reflexology, acupressure and social touch. Interventions aimed at relaxation. Touch interventions including verbal and affective content.

Excluded: backrub,^[[1]](#footnote-1)^1 noxious sensory stimulation, studies aimed primarily at coma arousal,^[[2]](#footnote-2)^2 sensorimotor therapy, interventions for which interpersonal touch comprised a minor component of an extensive rehabilitation programme, physical assessment techniques, non-contact “therapeutic touch”.

**Comparator.**

Include: no comparator, no intervention, standard care, any other touch or non-touch based interventions aimed at improving psychological function.

**Outcomes.**

Include: quantitative outcomes relating to stress, including, physiological stress indicators, biochemical stress indicators, neuroendocrine indicators, sleep, pain, agitation, anxiety; qualitative findings linking to quantitative outcomes including emotional responses and patient preferences.

Exclude: gastric emptying, infection, conscious level, wound healing, nausea and vomiting.

**Study types.**

Include: studies reporting at least one relevant quantitative outcome.

Exclude: studies reported as case studies only, studies not reporting at least one relevant quantitative outcome.

**“Study cluster” composition.**

Include: studies reported in at least one full-text English language paper, readily available translations of non-English language records, English language abstracts of “sibling papers”, English language abstracts of non-English language sibling papers.

Exclude: studies reported only as abstracts.

**Eligibility criteria for supplementary systematic search**

**Population receiving intervention.**

Include: hospital inpatients.

Exclude: samples containing individuals < 16 years old, non-inpatients.

**Intervention.**

As for main systematic search.

**Interventionist.**

Include: patients’ relatives or friends.

**Comparator interventionist.**

Include: strangers, such as nurses, therapists, or researchers.

Exclude: studies without stranger interventionist.

**Outcomes.**

As for main systematic search.

**Study types.**

As for main systematic search.

**“Study cluster” composition.**

As for main systematic search.

**References**

Papathanassoglou, E. D. E., & Mpouzika, M. D. A. (2012). Interpersonal touch: physiological effects in critical care. *Biological Research for Nursing*, *14*(4), 431–443. doi:10.1177/1099800412451312

1. 1 Backrub interventions, defined as short (< 5 min), non-standardized interventions, were excluded on the basis that this intervention is associated with increased heart rate and decreased SvO_2_ (Papathanassoglou & Mpouzika, 2012). [↑](#footnote-ref-1)
2. 2 Coma arousal studies were excluded because we considered the mechanisms involved to be distinct from those involved in stress reduction. [↑](#footnote-ref-2)
