# Supplementary material for: Interpersonal touch interventions for patients in intensive care: A design‐oriented realist review
Source: Nurs Open. 2018 Oct 24;6(2):216–35. doi: 10.1002/nop2.200 (PMC6419112; doi:10.1002/nop2.200)
Supplement: Supplementary file 3 [file NOP2-6-216-s003.docx]

**Appendix S3: Data extraction form template**

**Study ID:**

**Description of touch interventions**

|  | **Intervention 1:** | **Intervention 2:** |
| --- | --- | --- |
|  |  |  |
| Delivered by: |  |  |
| Details of touch training |  |  |
| Number of times delivered: |  |  |
| Duration: |  |  |
| Frequency: |  |  |
| Timing of delivery: |  |  |
| Pre-touch events: |  |  |
| Intervention context: |  |  |
| Post touch events: |  |  |
| Description of intervention: |  |  |
| Skin sites: |  |  |
| Non-touch adjuncts: |  |  |
|  |  |  |
|  | **Intervention 3:** |  |
| Delivered by: |  |  |
| Number of times delivered: |  |  |
| Duration: |  |  |
| Frequency: |  |  |
| Timing of delivery: |  |  |
| Pre-touch events: |  |  |
| Intervention context: |  |  |
| Post touch events: |  |  |
| Description of intervention: |  |  |
| Skin sites: |  |  |
| Non-touch adjuncts: |  |  |

**Patient participants**

| **General** |  | | | |
| --- | --- | --- | --- | --- |
| Institutional setting |  | | | |
| Country |  | | | |
| **Inclusion criteria** |  | | | |
| Age |  | | | |
| Clinical status |  | | | |
| Admission duration |  | | | |
| Other |  | | | |
| **Exclusion criteria** |  | | | |
| **Eligibility assessment details** |  | | | |
|  | **Intervention 1** | **Intervention 1** | **Intervention 1** | **Total** |
| **Baseline demographic data** |  |  |  |  |
| Number of participants randomised |  |  |  |  |
| Age |  |  |  |  |
| Male |  |  |  |  |
| Female |  |  |  |  |
| Other |  |  |  |  |
| **Clinical details** |  |  |  |  |
| Analgesia use |  |  |  |  |
| Sedation use |  |  |  |  |
| Respiratory support |  |  |  |  |
| Single room |  |  |  |  |
| **Losses to follow-up**  **Reasons** |  |  |  |  |
| 1. |  |  |  |  |
| 2. |  |  |  |  |
| 3. |  |  |  |  |
| Number lost to follow-up |  |  |  |  |
| % lost to follow-up |  |  |  |  |
| Final number of participants evaluated |  |  |  |  |
| Intention to treat analysis | Yes/No/Not stated | | | |

**Outcome measures**

| **Primary outcome measures**  1.  2.  3. |
| --- |
| **Method of assessing primary outcome measures**  1.  2.  3. |
| **Timing of primary outcome measures**  1.  2.  3. |
| **Secondary outcome measures**  1.  2.  3. |
| **Method of assessing secondary outcome measures**  1.  2.  3. |
| **Timing of secondary outcome measures**  1.  2.  3. |
| **Qualitative method** |
| **Reviewer comments:** |

**Results**

|  | **Intervention 1** | **Intervention 2** | **Intervention 3** |
| --- | --- | --- | --- |
| Primary outcomes 1.  Time point: |  |  |  |
| Primary outcomes 2.  Time point: |  |  |  |
| Primary outcome 3.  Time point: |  |  |  |
|  |  |  |  |
| Secondary outcome 1.  Time point: |  |  |  |
| Scondary outcome 2.  Time point: |  |  |  |
| *Statistics used:*  **Main findings:** | | | |

| **Qualitative results reported:** |
| --- |

| **Negative outcomes**: |
| --- |

**Methods**

| Design: parallel group/cross over/other  Duration of trial:  Interval of assessment: |
| --- |

**Methodological quality**

| **Major criteria** | | | **Method** |
| --- | --- | --- | --- |
| **Sampling method** | | |  |
| **1. Generation of randomisation sequence**  Any information given Y/N/unsure | | |  |
| **2. Allocation concealment**  Adequate  Unclear  Inadequate  Not used | | ☐  ☐  ☐  ☐ |  |
| **3. Blinding**  Participant  Researcher  Outcome assessor | Y/N/unsure  Y/N/unsure  Y/N/unsure | |  |
| **4. Loss to follow-up**  Were all randomised participants included in the analysis in the groups to which they were randomised? Y/N/unsure | | |  |
| **Other criteria** | | | Notes |
| 5. Were groups comparable at baseline? Y/N/unsure | | |  |
| 6. Interventions adequately described? Y/N/unsure | | |  |

**Trial registration number & Funding body**

| Trial registration number:  Funding declared: Y/N/unsure Name: |
| --- |

**Relevant comments by the author**

| *Mechanism proposed for intervention:*  *Other:*  *References to other relevant studies:* |
| --- |

**Reviewer comments**

| *Does the study have relevance to the CIMOCs?*  *Elements of complexity impacting on mechanism operation*:    *Other comments*:  *Author correspondence required*: |
| --- |
