# Supplementary figures and images for: Interpersonal touch interventions for patients in intensive care: A design‐oriented realist review
Source: Nurs Open. 2018 Oct 24;6(2):216–35. doi: 10.1002/nop2.200 (PMC6419112; doi:10.1002/nop2.200)

#### Appendix S4: Document flow diagram for supplementary systematic search

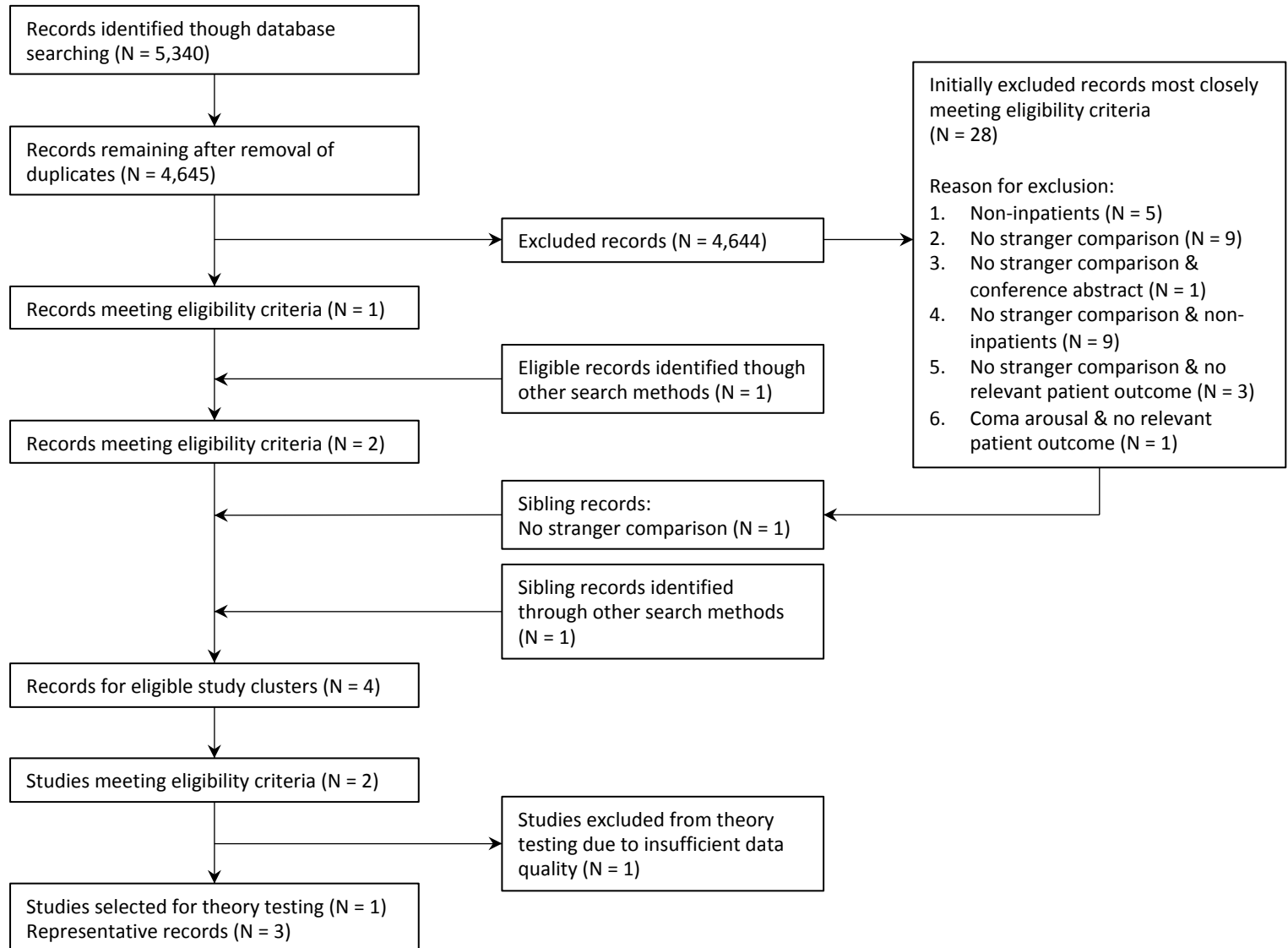

Supplement: Supplementary file 4 [file NOP2-6-216-s004.pdf]
