# Supplementary material for: Interpersonal touch interventions for patients in intensive care: A design‐oriented realist review
Source: Nurs Open. 2018 Oct 24;6(2):216–35. doi: 10.1002/nop2.200 (PMC6419112; doi:10.1002/nop2.200)
Supplement: Supplementary file 5 [file NOP2-6-216-s005.docx]

**Appendix S5: Summary of studies meeting eligibility criteria**

**Table S5.1 Summary of studies included in review**

| **Main systematic search** | | | | | |
| --- | --- | --- | --- | --- | --- |
| **Author(s), year of publication, country** | **Study design &**  **number recruited (*N*)** | **Context** | **Intervention** | **Comparator/control** | **Outcomes** |
| Bagheri-Nesami et al. (2015).  Iran | Three-arm RCT.  *N* = 90. | Coronary ICU.  Patient status: non-surgical diagnosed with moderate sleep disorder, aware of time and place.  No drugs < 5–6 h before sleeping. | Acupressure.  Head, face, ears, wrists, feet.  18 min, once daily × 3 | 1. Acupressure with valerian oil.  Head, face, ears, wrists, feet.  18 min, once daily × 3  2. Sham acupressure.  Head, face, ears, wrists, feet.  18 min, once daily × 3 | Sleep quantity.  Sleep quality. |
| Boitor et al. (2014, 2015), Martorella et al. (2014, 2016).  Canada | Two-arm RCT &  qualitative (descriptive).  *N* = 40. | Medical-surgical ICU.  Patient status: postoperative (elective cardiac surgery), able to answer questions.  Morphine received before each intervention. | Hand cream application & hand massage.  Lavender hand cream.  Gentle pressure stroking and other techniques.  15 min, 3 times a day × 1 | Hand cream application & handholding.  15 min, 3 times a day × 1 | Pain; pain intensity, pain behaviours, global pain experience.  HR, RR, BP, SpO_2_.  Muscle tension.  Qualitative: intervention acceptability & feasibility. |

**Table S5.1** *(Continued)*

| **Main systematic search** | | | | | |
| --- | --- | --- | --- | --- | --- |
| **Author(s), year of publication, country** | **Study design &**  **number recruited (*N*)** | **Context** | **Intervention** | **Comparator/control** | **Outcomes** |
| Çınar (2008), Çınar Yücel & Eşer (2015).  Turkey | Two-arm RCT.  *N* = 204. | Two respiratory ICUs.  Patient status: 100% ventilated, diagnosed with COPD, GCS 9–15, conscious and communicative.  No sedation therapy at time of intervention. | Massage (10 min) & acupressure (8 min).  Hand.  18 min, once daily × 5 | No intervention | HR, RR, BP.  Anxiety & dyspnoea. |
| Ebadi et al. (2015), Kavei et al. (2015).  Iran | Three-arm RCT.  *N* = 96. | Two open heart surgery ICUs.  Patient status: postoperative elective cardiac surgery, conscious, 100% ventilated (weaning).  No sedatives pre-intervention. | Foot reflexology massage.  20 min × 1 | 1. Surface touch to heels. Touch was without pressure and involved ^a^movement.  20 min × 1  2. Routine care (no touch). | HR, RR, BP, SpO_2_.  Ventilator weaning time.  ^b^(Anxiety and agitation). |
| Fakhr-Movahedi et al. (2014), Nobahar et al. (2014), Souri Lakie et al. (2012).  Iran | Two-arm cross over RCT.  *N* = 35. | General ICU.  Patient status: 100% ventilated, agitated,  GCS ≥ 9.  Considered clear of sedation. | Wrist holding without pressure.  5 minute × 1 | Nurse standing by patient. No touch. | SpO_2_.  ^b^(HR, RR, BP, T, agitation). |

**Table S5.1** *(Continued)*

| **Main systematic search** | | | | | |
| --- | --- | --- | --- | --- | --- |
| **Author(s), year of publication, country** | **Study design &**  **number recruited (*N*)** | **Context** | **Intervention** | **Comparator/control** | **Outcomes** |
| Henricson (2008),  Henricson, Berglund, et al. (2008), Henricson, Ersson, et al. (2008), Henricson et al. (2009).  Sweden | Two-arm RCT, *N* = 45 &  qualitative (phenomenological hermeneutics), *n* = 6. | Two general ICUs.  Patient status: 84% ventilated, minimally responsive to restless. Patients receiving oestrogen treatment excluded.  No changes in midazolam or morphine allowed during intervention administration. | Tactile touch (slow stroking, soft/firm) to  hands, feet, stomach, head, face, chest, arms, legs.  Soft music & dimmed lights.  60 min, once daily × 5 | Standard treatment (rest hour): soft music and dimmed lights.  60 min, once daily × 5 | HR, BP.  Blood glucose, blood oxytocin.  Anxiety, sedation–agitation level.  Sedation & noradrenaline requirements.  Qualitative: the meaning of receiving tactile touch. |
| Kaur et al. (2012).  India | Quantitative descriptive.  *N* = 60. | Five ICUs (main, liver, respiratory, cardio-thoracic, gastroenterology).  Patient status: 90% medical, 53% ventilated, unconscious–conscious. | Foot massage & reflexology.  Unspecified duration  Twice a day × 6 | N.A. | HR, BP, SpO_2_. |
| Korhan et al. (2014).  Turkey | Two-arm RCT.  *N* = 60. | ICU.  Patient status: 100% ventilated, GCS ≥ 9. Sedation (propofol) stopped 30 min before the start of the intervention. | Reflexology  Hands, feet, ears.  30 min, twice a day × 5 | No intervention.  30 min, twice a day × 5 | HR, RR, BP.  Sedation–agitation level. |
| Maa et al. (2013), Wang & Maa (2006).  Taiwan, China | Three-arm RCT.  *N* = 110. | Three medical ICUs & one respiratory care centre.  Patient status: 100 % ventilated, diagnosed with coma, GCS < 7.  Not receiving sedatives or opiates. | Acupressure  Shoulders, wrists, hands, below knees.  10 min, once daily × 1 | 1. Acupressure  Shoulders, wrists, hands, below knees.  10 min, once daily × 2  2. Standard treatment. | HR, RR, BP, SpO_2_. Ventilation parameters; tidal volume, rapid shallow breathing index minute ventilation, dynamic lung compliance, ^b^maximal inspiratory pressure. |

**Table S5.1** *(Continued)*

| **Main systematic search** | | | | | |
| --- | --- | --- | --- | --- | --- |
| **Author(s), year of publication, country** | **Study design &**  **number recruited (*N*)** | **Context** | **Intervention** | **Comparator/control** | **Outcomes** |
| Olleveant (2003).  UK | Three-arm RCT.  *N* = 150. | General ICU.  Patient status: 80% ventilated.  Level of anaesthesia: non to minimal, 73%; moderate to high, 27%. | Leg massage with almond oil.  14–20 min  Repeated once after three days. | 1. Leg massage with almond and bergamot oil.  14–20 min  Repeated once after three days.  2. No intervention. | HR, RR, BP.  Sedation–agitation level, pain score, behavioural activity, anxiety & depression.  Sedation & analgesia requirements.  Survival time in ICU & length of ICU stay.  Health status. |
| Tsay et al. (2005).  Taiwan, China | Two-arm RCT.  *N* = 52. | Two respiratory intermediate ICUs.  Patient status: 100% prolonged mechanical ventilation, 96% tracheostomies, diagnosed with COPD, alert and communicative.  Not receiving tranquilizers. | Massage (3 min; shoulder & arms) & acupressure points (12 min; hands, ears, wrists).  15 min, once daily × 10 | Massage (shoulder & arm) and handholding  15 min, once daily × 10 | HR, RR.  Anxiety, dyspnoea. |
| Yousefi, Naderi, & Daryabeigi (2015), Yousefi, Naderi, Daryabeigi, & Tajmiri (2015).  Iran | Two-arm RCT.  *N* = 64. | ICUs of two hospitals.  Patient status: 62% ventilated, GCS 9–15.  Receiving tranquilizers and narcotics (if required) > 6 h prior to sampling. | Patient’s relative provided; handholding, smooth touching of head and face, positive verbal support.  17 min, twice a day × 1 | Visitors not permitted. | HR, BP, SpO_2._ |

**Table S5.1** *(Continued)*

| **Supplementary systematic search** | | | | | |
| --- | --- | --- | --- | --- | --- |
| **Author(s), year of publication, country** | **Study design &**  **number recruited (*N*)** | **Context** | **Intervention** | **Comparator/control** | **Outcomes** |
| Adib-Hajbaghery et al. (2012, 2013, 2015).  Iran | Three-arm RCT.  *N* = 90. | Two coronary care units.  Patient status: male, diagnosed with acute coronary syndrome or acute myocardial infarction, without reduction in conscious level. | Whole body massage by patient’s companion.  60 min × 1 | 1. Whole body massage by nurse/massage therapist.  60 min × 1  2. Routine care. | Blood cortisol.  Satisfaction of massage.  ^c^(HR, RR, BP, T). |

*Note.* RR = respiratory rate; HR = heart rate; BP = blood pressure; SpO_2_ = peripheral oxygen saturation; T = temperature. N.A. = not applicable.

^a^A. Ebadi personal communication, 8^th^ June 2017, ^b^Results in English language reported only in abstract, full text only available in Persian. ^c^Results reported only for comparator and control groups.

**Table S5.2 Summary of studies meeting eligibility criteria excluded from review**

| **Main systematic search** | | | | | |
| --- | --- | --- | --- | --- | --- |
| **Author(s), year of publication, country** | **Study design & number recruited** | **Context** | **Intervention** | **Comparators** | **Outcomes** |
| Bauer & Dracup (1987).  USA | Quantitative descriptive.  *N* = 25. | Coronary ICU.  Patient status: diagnosis of acute myocardial infarction, testing performed > 4 h after any tranquilizers were taken. | Back massage.  6 min × 1 | N.A. | HR, BP, T.  Muscle tension, skin conductance. |
| Chen et al. (2012).  Taiwan, China | Two-arm RCT.  *N* = 85. | ICU.  Patient status: conscious and communicable, acute physiology score < 15 (stable, less critical ICU patients).  Sleeping medication not taken for > 1 month. | Acupressure  Wrist and feet.  18 min × 1 | Regular treatment. | Sleep, sleepiness.  Heart rate variability. |
| Chugh (2006).  India | Quantitative descriptive.  *N* = 30. | Cardio-thoracic ICU (days one and two); two post-operative wards (days three and four).  Patient status: postoperative cardiac surgery, extubated.  Analgesia regime. | Foot massage  10 min, twice daily × 4  (× 2 in ICU) | N.A. | HR, RR, BP.  Pain intensity.  Acceptability of intervention.  (No ICU time series). |
| Ghazal (2014a)  Syria | Three-arm quantitative non-randomised.  *N* = 45. | ICUs in two hospitals.  Patient status: able to provide written consent, reporting sleep disorder. | Effleurage back massage.  20 min × 1 | 1. Facial massage  20 minute × 1  2. Routine care with a 6-min rest period.  OD × 3 | Sleep quality |

**Table S5.2** *(Continued).*

| **Main systematic search** | | | | | |
| --- | --- | --- | --- | --- | --- |
| **Author(s), year of publication, country** | **Study design & number recruited** | **Context** | **Intervention** | **Comparators** | **Outcomes** |
| Ghazal (2014b).  Syria | Two-arm RCT.  *N* = 30 . | Cardiac ICU.  Patient status: postoperative cardiac surgery, fully conscious.  Analgesia received 4 h before first massage. | Hand & foot massage.  20 min, twice a day × 1 | Researcher sat beside patient. | Pain intensity.  HR, RR, BP.  (Time series data not reported). |
| Gunnarsdottir & Jonsdottir (2007).  Iceland | Two-arm RCT.  *N* = 11. | Pre-surgery (day one), ICU (day two), thoracic unit (days three–five).  Patient status: postoperative elective cardiac surgery, extubated, alert and mentally coherent. | Foot reflexology  30 min, once daily × 5  (× 1 in ICU) | Cream applied to feet.  1 min, once daily × 5  (× 1 in ICU) | Anxiety.  HR, RR, BP. |
| Hatefi et al*.* (2015).  Iran | Two-arm RCT.  *N* = 108. | ICU.  Patient status: 87% diagnosed with multiple trauma, GCS 7–12. | Whole body massage by patient’s relative.  45 min × 1 | Routine care | HR, RR, BP, T.  GCS.  Arterial blood gas parameters; SaO_2_, PaO_2_, PaCO_2_, HCO_3_, pH. |
| Hayes & Cox (1999).  UK | Quantitative descriptive.  *N* = 25. | Two critical care units.  Patient status: 52% ventilated. | Foot massage.  5 min × 2.7 (mean) | N.A. | HR, RR, BP, SpO_2_.  (Time series data not reported). |
| Henneman (1989).  USA | Two-arm RCT.  *N* = 26. | Medical, surgical and cardiac ICUs.  Patient status: 100% ventilated (weaning), alert enough to follow simple commands. | Handholding and verbal interaction. | Researcher present.  No verbal or tactile interaction. | HR, RR, BP. |

**Table S5.2** *(Continued).*

| **Main systematic search** | | | | | |
| --- | --- | --- | --- | --- | --- |
| **Author(s), year of publication, country** | **Study design & number recruited** | **Context** | **Intervention** | **Comparators** | **Outcomes** |
| ^a^Jamaati et al. (2015).  Iran | Three-arm RCT.  *N* = 99. | Surgical ICU.  Patient status: GCS 10–15. | Whole body massage by patient’s relative.  30 min × 1 | 1. Whole body massage by nurse.  30 min × 1  2. Routine care. | HR, RR, BP, SpO_2_.  Pain intensity. |
| Krucoff et al. (2001), Seskevich et al. (2004).  USA | Five-arm RCT.  *N* = 150. | Coronary ICU.  Patient status: scheduled for percutaneous coronary intervention, conscious. | Gentle static touch.  Hands placed in sequence of positions.  30 min × 1 | 1. Stress management.  2. Imagery.  3. Remote prayer.  4. Standard therapy. | Total ischemic burden & post-PCI ischemia.  Heart rate variability.  Mood & dyspnoea.  Adverse clinical endpoints & mortality. |
| Lindgren (2012), Lindgren et al. (2013).  Sweden | Two-arm RCT.  *N* = 20. | ICU.  Patient status; postoperative elective aortic surgery. Received continuous epidural analgesia. | Touch massage.  Hands, arms, feet & legs. Pressure and velocity of touch measured with custom-made device.  60 min × 1 | Rest in presence of health professionals. | Anxiety.  RR, BP, SpO_2_.  Heart rate variability.  Serum cortisol, insulin & glucose. |
| Richards (1993), Richards (1998).  USA | Three-arm RCT & qualitative descriptive.  *N* = 71. | Medical critical care unit with ventilation facility.  Patient status: male, diagnosed with cardiovascular illness, alert and oriented, not ventilated.  A minority of patients received sedatives and/or analgesics with sedative effects. | Back massage & dimmed lights.  6 min × 1 | 1. Initial training session and relaxation audiotape.  12.5 min × 1  2. Rest period with dimmed lights.  6 min × 1 | Sleep.  HR, RR.  Muscle tension, anxiety.  Qualitative: perceptions relating to pleasure and effectiveness of intervention. |

**Table S5.2** *(Continued).*

| **Main systematic search** | | | | | |
| --- | --- | --- | --- | --- | --- |
| **Author(s), year of publication, country** | **Study design & number recruited** | **Context** | **Intervention** | **Comparators** | **Outcomes** |
| Roy (2008).  India | Quantitative descriptive & qualitative descriptive.  *N* = 30. | Cardiac ICU.  Patient status: postoperative cardiac surgery. | Back massage  Unspecified duration.  Once daily × 3 | N.A. | Sleep (time series data not reported).  Qualitative: factors causing insomnia. |
| Shinde & Anjum (2014).  India | Two-arm quantitative non-randomised.  *N* = 60. | ICU.  Patient status: conscious and communicative, not ventilated. | Back massage.  10 min, once daily × 3 | Usual care. | Sleep.  (Time series data not reported). |
| Stevensen (1994).  UK | Four-arm RCT, *N* = 100 & quantitative descriptive,  *n* = 50. | ICU.  Patient status: postoperative cardiac surgery, conscious, extubated. | Foot massage with plain oil.  20 min × 1 | 1. Foot massage with neroli oil. 20 min × 1  2. General chat. 20 min × 1  3. Routine care. 20 min × 1 | HR, RR, BP.  Anxiety & pain.  Quantitative descriptive: perceptions of massage and its benefits. |
| ^a^Vahedian-Azimi et al. (2014).  Iran | Two-arm RCT.  *N* = 90. | General ICU.  Patient status: GCS 7–12. | Whole body massage by patient’s relative.  60 min × 1 | Routine care. | HR, RR, BP.  GCS. |
| **Supplementary systematic search** | | | | | |
| ^a^Jamaati et al. (2015).  Iran | Three-arm RCT.  *N* = 99. | Surgical ICU.  Patient status: GCS 10–15. | Whole body massage by patient’s relative.  30 min × 1 | 1. Whole body massage by nurse. 30 min × 1  2. Routine care. | HR, RR, BP, SpO_2_.  Pain intensity. |

*Note.* RR = respiratory rate; HR = heart rate; BP = blood pressure; SpO_2_ = peripheral oxygen saturation; T = temperature. N.A. = not applicable.

^a^Vahedian-Azimi et al. (2014) and Jamaati et al. (2015) were excluded due to statistically improbable between-study similarities for reported outcome data for the control groups (data pending reanalysis according to author, A. Vahedian-Azimi, personal communication, 7^th^ November 2016). All other studies were excluded due to a lack of sufficiently relevant data.

**References**

Adib-Hajbaghery, M., Abasi, A., Rajabi-Beheshtabad, R., & Azizi-Fini, I. (2012). The effects of massage therapy by the patient’s relative on vital signs of males admitted in critical care unit. *Nursing and Midwifery Studies*, *1*(1), 16–21. doi:10.5812/nms.7903

Adib-Hajbaghery, M., Rajabi-Beheshtabad, R., & Abasi, A. (2013). Effect of whole body massage by patient’s companion on the level of blood cortisol in coronary patients: a randomized controlled trial. *Nursing and Midwifery Studies*, *2*(3), 10–21. doi:10.5812/nms.13781

Adib-Hajbaghery, M., Rajabi-Beheshtabad, R., & Ardjmand, A. (2015). Comparing the effect of whole body massage by a specialist nurse and patients’ relatives on blood cortisol level in coronary patients. *ARYA Atherosclerosis*, *11*(2), 126–32. Retrieved from http://arya.mui.ac.ir/index.php/arya

Bagheri-Nesami, M., Gorji, M. A. H., Rezaie, S., Pouresmail, Z., & Cherati, J. Y. (2015). Effect of acupressure with valerian oil 2.5% on the quality and quantity of sleep in patients with acute coronary syndrome in a cardiac intensive care unit. *Journal of Traditional and Complementary Medicine*, *5*(4), 241–247. doi:10.1016/j.jtcme.2014.11.005

Bauer, W. C., & Dracup, K. A. (1987). Physiological effects of back massage in patients with acute myocardial infarction. *Focus on Critical Care*, *14*(6), 42–46.

Boitor, M., Martorella, G., Arbour, C., Michaud, C., & Gélinas, C. (2014). P20: Evaluation of the preliminary effectiveness of hand-massage therapy on postoperative pain of cardiac surgery adults in the intensive care unit. *Abstract presented at the 35th Annual Scientific Meeting of the Canadian Pain Society, Quebec City, Canada*. *Pain Research & Management, 19*(3), e64. Retrieved from https://www.ncbi.nlm.nih.gov/pmc/articles/PMC4158963/

Boitor, M., Martorella, G., Arbour, C., Michaud, C., & Gélinas, C. (2015). Evaluation of the preliminary effectiveness of hand massage therapy on postoperative pain of adults in the intensive care unit after cardiac surgery: A pilot randomized controlled trial. *Pain Management Nursing*, *16*(3), 354–366. doi:10.1016/j.pmn.2014.08.014

Chen, J.-H., Chao, Y.-H., Lu, S.-F., Shiung, T.-F., & Chao, Y.-F. (2012). The effectiveness of valerian acupressure on the sleep of ICU patients: A randomized clinical trial. *International Journal of Nursing Studies*, *49*(8), 913–920. doi:10.1016/j.ijnurstu.2012.02.012

Chugh, D. (2006). A study to determine the effect of ten minutes foot massage on two phases of postoperative coronary artery bypass (CABG) patients of selected variables. *Asian Journal of Cardiovascular Nursing*, *14*(2), 13–18.

Çınar, Ş. (2008). *The effect on anxiety and cost of hand massage and acupressure therapy in patients having mechanical ventilation support. Mekanik ventilasyon desteğinde olan hastalarda el masajı ve akupressur uygulamasının anksiyete ve maliyete etkisi.* (Doctoral dissertation, Ege University, Turkey). Retrieved from http://en.academicresearch.net/

Çınar Yücel, Ş., & Eşer, İ. (2015). Effects of hand massage and acupressure therapy for mechanically ventilated patients. *Journal of Human Sciences*, *12*(2), 881–896. doi:10.14687/ijhs.v12i2.3054

Ebadi, A., Kavei, P., Moradian, S. T., & Saeid, Y. (2015). The effect of foot reflexology on physiologic parameters and mechanical ventilation weaning time in patients undergoing open-heart surgery: A clinical trial study. *Complementary Therapies in Clinical Practice*, *21*(3), 188–92. doi:10.1016/j.ctcp.2015.07.001

Fakhr-Movahedi, A., Nobahar, M., & Bolhasani, M. (2014). The effect of touch on the vital signs of agitated patients undergoing mechanical ventilation: An interventional study. *Journal of Urmia Nursing and Midwifery Faculty*, *12*(10), 17–24. Abstract retrieved from http://unmf.umsu.ac.ir/index.php?slc_lang=en&slc_sid=1

Ghazal, S. (2014a). Effects of back massage and facial massage on sleep quality in critically ill patients. *Tishreen University Journal for Research and Scientific Studies - Health Sciences Series*, *36*(1), 171–179. Retrieved from http://journal.tishreen.edu.sy/index.php/hlthscnc/index

Ghazal, S. (2014b). Effects of foot and hand massage on pain of open heart surgery patients in intensive care units. *Tishreen University Journal for Research and Scientific Studies - Health Sciences Series*, *36*(2), 167–178. Retrieved from http://journal.tishreen.edu.sy/index.php/hlthscnc/index

Gunnarsdottir, T. J., & Jonsdottir, H. (2007). Does the experimental design capture the effects of complementary therapy? A study using reflexology for patients undergoing coronary artery bypass graft surgery. *Journal of Clinical Nursing*, *16*(4), 777–785. doi:10.1111/j.1365-2702.2006.01634.x

Hatefi, M., Jaafarpour, M., Khani, A., Khajavikhan, J., & Kokhazade, T. (2015). The effect of whole body massage on the process and physiological outcome of trauma ICU patients: A double-blind randomized clinical trial. *Journal of Clinical and Diagnostic Research*, *9*(6), UC05–UC08. doi:10.7860/JCDR/2015/12756.6096

Hayes, J., & Cox, C. (1999). Immediate effects of a five-minute foot massage on patients in critical care. *Intensive and Critical Care Nursing*, *15*(2), 77–82. doi:10.1016/S0964-3397(99)80003-2

Henneman, A. E. (1989). Effect of nursing contact on the stress response of patients in critical care. *Heart & Lung*, *18*(5), 483–489.

Henricson, M. (2008). *Tactile touch in intensive care: Nurses’ preparation, patients’ experiences and the effect on stress parameters.* (Doctoral dissertation, University of Borås, Sweden). Retrieved from http://www.diva-portal.org/smash/record.jsf?pid=diva2%3A876856&dswid=-8187

Henricson, M., Berglund, A.-L., Määttä, S., Ekman, R., & Segesten, K. (2008). The outcome of tactile touch on oxytocin in intensive care patients: a randomised controlled trial. *Journal of Clinical Nursing*, *17*(19), 2624–3633. doi:10.1111/j.1365-2702.2008.02324.x

Henricson, M., Ersson, A., Määttä, S., Segesten, K., & Berglund, A.-L. (2008). The outcome of tactile touch on stress parameters in intensive care: A randomized controlled trial. *Complementary Therapies in Clinical Practice*, *14*(4), 244–254. doi:10.1016/j.ctcp.2008.03.003

Henricson, M., Segesten, K., Berglund, A.-L., & Määttä, S. (2009). Enjoying tactile touch and gaining hope when being cared for in intensive care—A phenomenological hermeneutical study. *Intensive and Critical Care Nursing*, *25*(6), 323–331. doi:10.1016/j.iccn.2009.07.001

Jamaati, H., Vahedian-Azimi, A., Ebadi, A., Ahmadi, F., Saadat, S., Kashafi, M. B., … Hashemian, S. M. (2015). Therapeutic effect of massage on the patients in intensive care unit. *Archives of Critical Care Medicine*, *1*(1), e519. doi:10.5812/accm.519

Kaur, J., Kaur, S., & Bhardwaj, N. (2012). Effect of ‘foot massage and reflexology’ on physiological parameters of critically ill patients. *Nursing and Midwifery Research Journal*, *8*(3), 223–233. Retrieved from http://medind.nic.in

Kavei, P., Ebadi, A., Saeed, Y., Moradian, S. T., & Sedigh-Rahimabadi, M. (2015). Effect of foot reflexology on anxiety and agitation in patients under mechanical ventilation after open heart surgery: A randomized clinical trial study. *Journal of Clinical Nursing and Midwifery*, *4*(1), 16–26. Abstract retrieved from http://jcnm.skums.ac.ir/index.php?slc_lang=en&slc_sid=1

Korhan, E. A., Khorshid, L., & Uyar, M. (2014). Reflexology: its effects on physiological anxiety signs and sedation needs. *Holistic Nursing Practice*, *28*(1), 6–23. doi:10.1097/HNP.0000000000000007

Krucoff, M. W., Crater, S. W., Green, C. L., Maas, A. C., Seskevich, J. E., Lane, J. D., … Koenig, H. G. (2001). Integrative noetic therapies as adjuncts to percutaneous intervention during unstable coronary syndromes: Monitoring and Actualization of Noetic Training (MANTRA) feasibility pilot. *American Heart Journal*, *142*(5), 760–769. doi:10.1067/mhj.2001.119138

Lindgren, L. (2012). *Emotional and physiological responses to touch massage*. Umeå University. Retrieved from http://www.diva-portal.org/smash/get/diva2:568111/fulltext01.pdf

Lindgren, L., Lehtipalo, S., Winsö, O., Karlsson, M., Wiklund, U., & Brulin, C. (2013). Touch massage: a pilot study of a complex intervention. *Nursing in Critical Care*, *18*(6), 269–77. doi:10.1111/nicc.12017

Maa, S.-H., Wang, C.-H., Hsu, K.-H., Lin, H.-C., Yee, B., MacDonald, K., … Abraham, I. (2013). Acupressure improves the weaning indices of tidal volumes and rapid shallow breathing index in stable coma patients receiving mechanical ventilation: Randomized controlled trial. *Evidence-Based Complementary and Alternative Medicine*, *2013*, Article ID 723128. doi:10.1155/2013/723128

Martorella, G., Boitor, M., & Gélinas, C. (2016). (554) Hand massage therapy for pain management after cardiac surgery: acceptability and feasibility in the intensive care unit. *Abstract presented at the 35th Annual Scientific Meeting of the American Pain Society, Austin, TX*. *Journal of Pain, 17*(4), S113. doi:10.1016/j.jpain.2016.01.464

Martorella, G., Boitor, M., Michaud, C., & Gélinas, C. (2014). Feasibility and acceptability of hand massage therapy for pain management of postoperative cardiac surgery patients in the intensive care unit. *Heart & Lung*, *43*(5), 437–444. doi:10.1016/j.hrtlng.2014.06.047

Nobahar, M., Bolhasani, M., Fakhr-Movahedi, A., & Ghorbani, R. (2014). Effects of touch on agitation in patients under mechanical ventilation. *Koomesh*, *15*(3), 325–333. Abstract retrieved from http://koomeshjournal.semums.ac.ir/index.php?slc_lang=en&slc_sid=1

Olleveant, N. A. (2003). *Physiological and psychological effects of aromatherapy massage on critically ill patients*. (Doctoral dissertation, University of Liverpool, U.K.). Retrieved from http://ethos.bl.uk/OrderDetails.do?uin=uk.bl.ethos.275050

Richards, K. C. (1993). *The effect of a muscle relaxation, imagery, and relaxing music intervention and a back massage on the sleep and psychophysiological arousal of elderly males hospitalized in the critical care environment*. (Doctoral dissertation, University of Texas at Austin, U.S.A.). Retrieved from http://las.sinica.edu.tw:1085/*eng

Richards, K. C. (1998). Effect of a back massage and relaxation intervention on sleep in critically ill patients. *American Journal of Critical Care*, *7*(4), 288–299.

Roy, C. (2008). Effect of back massage in promoting sleep. *Asian Journal of Cardiovascular Nursing*, *16*(2), 10–14.

Seskevich, J. E., Crater, S. W., Lane, J. D., & Krucof, M. W. (2004). Beneficial effects of noetic therapies on mood before percutaneous intervention for unstable coronary syndromes. *Nursing Research*, *53*(2), 116–121. doi:10.1097/00006199-200403000-00007

Shinde, M. B., & Anjum, S. (2014). Effectiveness of slow back massage on quality of sleep among ICU patent’s. *International Journal of Science and Research*, *3*(3), 292–298. Retrieved from http://www.ijsr.net

Souri Lakie, A., Bolhasani, M., Nobahar, M., Fakhr Movahedi, A., & Mahmoudi, H. (2012). The effect of touch on the arterial blood oxygen saturation in agitated patients undergoing mechanical ventilation. *Iranian Journal of Critical Care Nursing*, *5*(3), 125–132. Retrieved from http://inhc.ir/index.php?sid=1&slc_lang=en

Stevensen, C. (1994). The psychophysiological effects of aromatherapy massage following cardiac surgery. *Complementary Therapies in Medicine*, *2*(1), 27–35. doi:10.1016/0965-2299(94)90156-2

Tsay, S.-L., Wang, J.-C., Lin, K.-C., & Chung, U.-L. (2005). Effects of acupressure therapy for patients having prolonged mechanical ventilation support. *Journal of Advanced Nursing*, *52*(2), 142–150. doi:10.1111/j.1365-2648.2005.03576.x

Vahedian-Azimi, A., Ebadi, A., Asghari Jafarabadi, M., Saadat, S., & Ahmadi, F. (2014). Effect of massage therapy on vital signs and GCS scores of ICU patients: A randomized controlled clinical trial. *Trauma Monthly*, *19*(3), 19–25. doi:10.5812/traumamon.17031

Wang, C.-H., & Maa, S.-H. (2006, September). *Acupressure – effects on weaning indices in coma patients with ventilation support.* Poster discussion presented at the ERS International Congress. Munich, Germany. Abstract retrieved from http://www.ers-education.org/events/international-congress/munich-2006.aspx?idParent=5819

Yousefi, H., Naderi, M., & Daryabeigi, R. (2015). The effect of sensory stimulation provided by family on arterial blood oxygen saturation in critical care patients. *Iranian Journal of Nursing and Midwifery Research*, *20*(1), 63–68. Retrieved from http://ijnmr.mui.ac.ir/index.php/ijnmr

Yousefi, H., Naderi, M., Daryabeigi, R., & Tajmiri, M. (2015). The effect of sensory stimulation provided by family on systolic and diastolic blood pressure and heart rate in critical care patients. *International Journal of Current Research*, *7*(1), 11621–11626. Retrieved from http://www.journalcra.com
