# Supplementary material for: Interpersonal touch interventions for patients in intensive care: A design‐oriented realist review
Source: Nurs Open. 2018 Oct 24;6(2):216–35. doi: 10.1002/nop2.200 (PMC6419112; doi:10.1002/nop2.200)
Supplement: Supplementary file 6 [file NOP2-6-216-s006.docx]

**Appendix S6: Unavailable and excluded records for studies most closely meeting eligibility criteria**

**S6.1 Main systematic search**

**Excluded records for studies most closely meeting eligibility criteria.**

Abbasi, M., Mohammadi, E. & Sheaykh Rezayi, A. (2009). Effect of a regular family visiting program as an affective, auditory, and tactile stimulation on the consciousness level of comatose patients with a head injury. *Japan Journal of Nursing Science, 6*(1), 21–26. doi:10.1111/j.1742-7924.2009.00117.x [Reason for exclusion: no relevant outcome.]

Albert, N. M., Gillinov, A. M., Lytle, B. W., Feng, J., Cwynar, R., & Blackstone, E. H. (2009). A randomized trial of massage therapy after heart surgery. *Heart & Lung*, *38*(6), 480–490. doi:10.1016/j.hrtlng.2009.03.001 [Reason for exclusion: not ICU.]

Aliha, Z., Mehranfar, N., Hamidi, M.N., Nezhad, A.K. & Saatchi, K. (2015). The effect of acupressure on spontaneous breathing in patients under mechanical ventilation, *Avicenna Journal of Phytomedicine,* *5*(Supplement 1) 84–85. Retreived from http://ajp.mums.ac.ir [Reason for exclusion: conference abstract.]

Bagheri-Nesami, M., Shorofi, S.A., Zargar, N., Sohrabi, M., Gholipour-Baradari, A. & Khalilian, A. (2014). The effects of foot reflexology massage on anxiety in patients following coronary artery bypass graft surgery: A randomized controlled trial. *Complementary Therapies in Clinical Practice,* *20*(1), 42–47. doi:10.1016/j.ctcp.2013.10.006 [Reason for exclusion: not ICU.]

Bauer, B. A., Cutshall, S. M., Wentworth, L. J., Engen, D., Messner, P. K., Wood, C. M., … Sundt, T. M. (2010). Effect of massage therapy on pain, anxiety, and tension after cardiac surgery: A randomized study. *Complementary Therapies in Clinical Practice*, *16*(2), 70–75. doi:10.1016/j.ctcp.2009.06.012 [Reason for exclusion: not ICU.]

Catapano, J.M. (1977). Effects of Touch on Emotional Arousal in Surgical Intensive Care Patients. *Abstracts of Hospital Management Studies,* *14*(2), 18174 SC: 18133p. [Reason for exclusion: conference abstract.]

Cox, C. & Hayes, J. (1999). Physiologic and psychodynamic responses to the administration of therapeutic touch in critical care. *Complementary Therapies in Nursing and Midwifery,* *5*(3), 87–92. doi: 10.1016/S1353-6117(99)80026-2 [Reason for exclusion: therapeutic touch.]

Cutshall, S. M., Wentworth, L. J., Engen, D., Sundt, T. M., Kelly, R. F., & Bauer, B. A. (2010). Effect of massage therapy on pain, anxiety, and tension in cardiac surgical patients: A pilot study. *Complementary Therapies in Clinical Practice*, *16*(2), 92–95. doi:10.1016/j.ctcp.2009.10.006 [Reason for exclusion: not ICU.]

Dunn, C., Sleep, J. & Collett, D. (1995). Sensing an improvement: An experimental study to evaluate the use of aromatherapy, massage and periods of rest in an intensive care unit. *Journal of Advanced Nursing,* *21*(1), 34–40. doi:10.1016/S1353-6117(99)80026-2 [Reason for exclusion: includes participants <16 years old.]

Hattan, J., King, L., & Griffiths, P. (2002). The impact of foot massage and guided relaxation following cardiac surgery: a randomized controlled trial. *Journal of Advanced Nursing*, *37*(2), 199–207. doi:10.1046/j.1365-2648.2002.02083.x [Reason for exclusion: not ICU.]

Kaur, S., Lobo, D.J. & Latha, T. (2013). Role of foot and hand massage on the anxiety for post operative open heart surgery patients: A randomized control trial. *International Journal of Nursing Education,* *5*(2), 205–208. doi:10.5958/j.0974-9357.5.2.096 [Reason for exclusion: not ICU.]

Kshettry, V.R., Carole, L.F., Henly, S.J., Sendelbach, S. & Kummer, B. (2006). Complementary alternative medical therapies for heart surgery patients: Feasibility, safety, and impact. *The Annals of Thoracic Surgery,* *81*(1), 201–205. doi:10.1016/j.athoracsur.2005.06.016 [Reason for exclusion: not ICU.]

Lewis, P., Nichols, E., Mackey, G., Fadol, A., Sloane, L., Villagomez, E & Liehr, P. (1997). The effect of turning and backrub on mixed venous oxygen saturation in critically ill patients. *American Journal of Critical Care,* *6*(2), 132–140. [Reason for exclusion: backrub.]

Li, D., Miaskowski, C., Burkhardt, D. & Puntillo, K. (2009). Evaluations of physiologic reactivity and reflexive behaviors during noxious procedures in sedated critically ill patients. *Journal of Critical Care,* *24*(3), 472.e9-472.e13. doi:10.1016/j.jcrc.2008.07.005 [Reason for exclusion: noxious comparison.]

Li, D.T-Y.L. (2007). Evaluations of physiologic and behavioral responses to noxious procedures in sedated critically ill adult patients. (Doctoral dissertation, University of California, San Francisco, U.S.A.). [Reason for exclusion: noxious comparison.]

MacCune, S. (2010). Effect of back massage on sleep among post-operative CABG and valve replacement patients. *Nursing Journal of India*, *101*(4), 86–88. [Reason for exclusion: not ICU.]

MacIntyre, B., Hamilton, J., Fricke, T., Ma, W., Mehle, S., & Michel, M. (2008). The efficacy of healing touch in coronary artery bypass surgery recovery: a randomized clinical trial. *Alternative Therapies*, *14*(4), 24–32. [Reason for exclusion: not ICU.]

Smith, K.D. (2004). The effects of nursing back rub on pain and wound cytokines and the relationship between pre-CABG mood and post-CABG wound cytokines. (Doctoral dissertation, University of Tenessee, Knoxville, U.S.A). Retrieved from http://trace.tennessee.edu/utk_graddiss/2239 [Reason for exclusion: not ICU.]

Tyler, D.O., Winslow, E.H., Clark, A.P. & White, K.M. (1990). Effects of a 1-minute back rub on mixed venous oxygen saturation and heart rate in critically ill patients. *Heart & Lung,* *19*(5 Pt 2), 562–565. [Reason for exclusion: backrub.]

**Sibling records unavailable as supplement to main systematic search results.**

Henneman, E.A. (1985). *The effect of nursing contact on the stress response of patients weaning from mechanical ventilation.* (Unpublished master’s thesis, University of Colorado, Boulder, U.S.A).

**Potentially eligible non-English language records.**

Abed Saeidi, Z., Alavi Majd, H., Moshtagh Eshgh, Z., Imani, E., Ali Hosseini, T., & H. Alavi Majd (2004). The effects of foot massage on SpO_2_ in CVA patients at ICU of Shohadayeh Tajrish Hospital. *Journal of Faculty of Nursing & Midwifery of Shaheed Beheshti University of Medical Sciences And Health Services,* *14*(45), 27–33. [Persian].

Aliha, J.M., Behroozi, N., Peyrovi, H. & Mehran, A. (2013). The effect of foot reflexology massage on incisional pain in abdominal and chest surgery patients admitted to intensive Care Unit. *Iranian Journal of Cardiovascular Nursing,* *2*(2), 6–12. [Persian]

Anaraki, H.R., Abdollahi, A.A., Nasiri, H. & Vakili, M.A. (2001). Immediate effects of a five minutes back massage on patients’ physiological parameters in critical care unit. *Journal of Gorgan University of Medical Sciences,* *3*(2), 53–58. [Persian]**.**

Eimani, E., Moshtaqeshgh, Z., Ali Hoseini, T., Alavi Majd, H. & AbedSaeidi, J. (2009). The effect of foot massage on physiological indicators of female patients with CVA admitted in the ICU. *Journal of Shahid Sadoughi University of Medical Sciences,* *17*(2), 209–215. [Persian].

Hajihosseini, F., Avazeh, A., Elahi, N., Shariati, A. & Sori, A. (2006). The effect of massage on comatose patients’ vital signs, hospitalised in intensive care units. *Arak Medical University Journal*, *9*(3) 26–35. [Persian].

Khost, N., Moshtagh, Z., Imani, E. & Alavi-Majd, H. (2006). Effect of foot massage on physiologic indicators in critically ill patients admitted in the I.C.U. *Journal of Shahid Sadoughi University of Medical Sciences*, *14*(3) 69–75. [Persian].

Kim, M.Y., Jean, S.Y., Song, Y.H., Choi, E.J, Kim, J.H., Kim, M.S., Joo, M.S. & Kim, N.S. (2006). The effects of head and neck massage on the sleep and anxiety of ICU patients. *Journal of Korean Clinical Nursing Research*, *11*(2) 49–61. [Korean].

Mehranfard, N., Aliha, J. M., Navidhamidi, M., Kazemnejad, A., & Saatchi, K. (2014). The effect of acupressure on anxiety in patients under mechanical ventilation. *Iranian Journal of Cardiovascular Nursing*, *3*(3), 48–57. [Persian].

Shaban, M., Amiry, P.H., Mehran, A. & Kahrary, S. (2004). Evaluation of immediate effect of foot massage on patient’s vital signs in a general intensive care unit. *Journal of Hayat* *10*(1), 71–79. [Persian].

Yang, Y.-H. (2008). The effects of foot reflexology on anxiety, dyspnea, and weaning parameters among prolong mechanical ventilation patients. (Master's thesis, National Taipei College of Nursing, Taipei, Taiwan). [Chinese].

Zolriasatain, F., Bahraini, S., Hariri, G.H. & Khoda Karim, S. (2013). The effect of massage therapy on the physiological index of the patients hospitalized at different intensive care units of Shahid Beheshti University of Medical Sciences. *Journal of Urmia Nursing and Midwifery Faculty,* *11*(2), 1–11. [Persian].

**S6.2 Supplementary systematic search: excluded records for studies most closely meeting eligibility criteria**

Coan, J.A., Kasle, S., Jackson, A., Schaefer, H.S. & Davidson, R.J. (2013). Mutuality and the social regulation of neural threat responding. *Attachment & Human Development,* *15*(3), 303–315. doi:10.1080/14616734.2013.782656 [Reason for exclusion: non-inpatients.]

Coan, J.A., Beckes, L. & Allen, J.P. (2013). Childhood maternal support and social capital moderate the regulatory impact of social relationships in adulthood. *International Journal of Psychophysiology,* *88*(3), 224–31. doi:10.1016/j.ijpsycho.2013.04.006 [Reason for exclusion: non-inpatients.]

Coan, J.A., Schaefer, H.S. & Davidson, R.J. (2006). Lending a hand: Social regulation of the neural response to threat. *Psychological Science,* *17*(12), 1032–1039. doi:10.1111/j.1467-9280.2006.01832.x [Reason for exclusion: non-inpatients.]

Collinge, W., Kahn, J., Walton, T., Kozak, L., Bauer-Wu, S., Fletcher, K., Yarnold, P. & Soltysik, R. (2013). Touch, caring, and cancer: Randomized controlled trial of a multimedia caregiver education program. *Supportive Care in Cancer,* *21*(5), 1405–1414. doi: 10.1007/s00520-012-1682-6 [Reasons for exclusion: no stranger comparison and non-inpatients.]

Collinge, W., Kahn, J., Yarnold, P., Bauer-Wu, S. & McCorkle, R. (2007). Couples and cancer: feasibility of brief instruction in massage and touch therapy to build caregiver efficacy. *Journal of the Society for Integrative Oncology,* *5*(4), 147–154. doi: 10.2310/7200.2007.013 [Reasons for exclusion: no stranger comparison and non-inpatients.]

Ditzen, B., Neumann, I.D., Bodenmann, G., von Dawans, B., Turner, R.A., Ehlert, U. & Heinrichs, M. (2007). Effects of different kinds of couple interaction on cortisol and heart rate responses to stress in women. *Psychoneuroendocrinology,* *32*(5), 565–574. doi: 10.1016/j.psyneuen.2007.03.011 [Reasons for exclusion: no stranger comparison and non-inpatients.]

Dunning, T. & James, K. (2001). Complementary therapies in action–education and outcomes. *Complementary Therapies in Nursing & Midwifery,* *7*(4), 188–195. doi: 10.1054/ctnm.2001.0575 [Reason for exclusion: no stranger comparison.]

Edens, J.L., Larkin, K.T. & Abel, J.L. (1992). The effect of social support and physical touch on cardiovascular reactions to mental stress. *Journal of Psychosomatic Research,* *36*(4), 371–381. doi: 10.1016/0022-3999(92)90073-B [Reason for exclusion: non-inpatients.]

Ellis, V., Hill, J. & Campbell, H. (1995). Strengthening the family unit through the healing power of massage. *The American Journal of Hospice & Palliative Care,* *12*(5), 19–21. [Reasons for exclusion: no stranger comparison and no relevant outcome.]

Faurot, K.R., Gaylord, S.A. & Mann, J.D. (2007). Training family caregivers in hand and foot massage for hospitalized patients: Feasibility, challenges, and lessons learned. *Complementary Health Practice Review,* *12*(3), 203–226. doi:10.1177/1533210107307154 [Reasons for exclusion: no stranger comparison and no relevant outcome.]

Field, T., Hemandez-Reif, M., Taylor, S., Quintino, O. & Burman, I. (1997). Labor pain Is reduced by massage therapy. *Journal of Psychosomatic Obstetrics & Gynecology,* *18*(4), 286–291. doi: 10.3109/01674829709080701 [Reason for exclusion: no stranger comparison.]

Field, T., Figueiredo, B., Hernandez-Reif, M., Diego, M., Deeds, O. & Ascencio, A. (2008). Massage therapy reduces pain in pregnant women, alleviates prenatal depression in both parents and improves their relationships. *Journal of Bodywork and Movement Therapies,* *12*(2), 146–150. doi: 10.1016/j.jbmt.2007.06.003 [Reasons for exclusion: no stranger comparison and non-inpatients.]

Forchuk, C. (2001). Post-operative arm massage: A support for women with lymph node dissection and their families, *Sigma Theta Tau International: International Nursing Research Congress, June 2001*. [Reason for exclusion: no stranger comparison and conference abstract.]

Forchuk, C., Baruth, P., Prendergast, M., Holliday, R., Bareham, R., Brimner, S., Schulz, V., Chan, Y.C.L. & Yammine, N. (2004). Postoperative arm massage: A support for women with lymph node dissection. *Cancer Nursing,* *27*(1), 25–33. doi: 10.1097/00002820-200401000-00004 [Reason for exclusion: no stranger comparison.]

Goldstein, P., Shamay-Tsoory, S.G., Yellinek, S. & Weissman-Fogel, I. (2016). Empathy predicts an experimental pain reduction during touch. *The Journal of Pain*, *17*(10), 1049–1057. doi: 10.1016/j.jpain.2016.06.007 [Reason for exclusion: non-inpatients.]

Hasuo, H., Kanbara, K., Mizuno, Y., Nishiyama, J., Fukunaga, M. & Yunoki, N, (2015). A family caregiver’s relaxation enhances the gastric motility function of the patient: A crossover study. *BioPsychoSocial Medicine,* *9*: 21. doi:10.1186/s13030-015-0048-y [Reasons for exclusion: no stranger comparison and no relevant outcome.]

Hatefi, M., Jaafarpour, M., Khani, A., Khajavikhan, J. & Kokhazade, T. (2015). The effect of whole body massage on the process and physiological outcome of trauma ICU patients: A double-blind randomized clinical trial. *Journal of Clinical and Diagnostic Research,* *9*(6), UC05–UC08. doi:10.7860/JCDR/2015/12756.6096 [Reason for exclusion: no stranger comparison.]

Holt-Lunstad, J., Birmingham, W.A. & Light, K.C. (2008). Influence of a ‘warm touch’ support enhancement intervention among married couples on ambulatory blood pressure, oxytocin, alpha amylase, and cortisol. *Psychosomatic Medicine,* *70*(9), 976–985. [Reasons for exclusion: no stranger comparison and non-inpatients.]

Holt-Lunstad, J., Birmingham, W. & Light, K.C. (2011). The influence of depressive symptomatology and perceived stress on plasma and salivary oxytocin before, during and after a support enhancement intervention. *Psychoneuroendocrinology,* *36*(8), 1249–1256. doi:10.1097/PSY.0b013e318187aef7 [Reasons for exclusion: no stranger comparison and non-inpatients.]

Kozak, L., Vig, E., Simons, C., Eugenio, E., Collinge, W. & Chapko, M. A (2013). Feasibility study of caregiver-provided massage as supportive care for veterans with cancer. *The Journal of Supportive Oncology,* *11*(3), 133–143. doi:10.12788/j.suponc.0008 [Reasons for exclusion: no stranger comparison and non-inpatients.]

Moattari, M., Shirazi, F.A., Sharifi, N. & Zareh, N. (2016). Effects of a sensory stimulation by nurses and families on level of cognitive function, and basic cognitive sensory recovery of comatose patients with severe traumatic brain injury: A randomized control trial. *Trauma Monthly,* *21*(4), e23531. doi:10.5812/traumamon.23531 [Reason for exclusion: coma arousal & no relevant outcome.]

Najafi, S.S., Rast, F., Momennasab, M., Ghazinoor, M., Dehghanrad, F. & Mousavizadeh, S.A. (2014). The effect of massage therapy by patients’ companions on severity of pain in the patients undergoing post coronary artery bypass graft surgery: A single-blind randomized clinical trial. *International Journal of Community Based Nursing and Midwifery, 2*(3), 128–135. [Reason for exclusion: no stranger comparison.]

Nummenmaa, L., Tuominen, L., Dunbar, R., Hirvonen, J., Manninen, S., Arponen, E., Machin, A., Hari, R., Jääskeläinen, I.P. & Sams, M. (2016). Social touch modulates endogenous μ-opioid system activity in humans. *NeuroImage,* *138*, 242–247. doi:10.1016/j.neuroimage.2016.05.063 [Reasons for exclusion: no stranger comparison and non-inpatients.]

Rowe, M. & Alfred, D. (1999). The effectiveness of slow-stroke massage in diffusing agitated behaviors in individuals with Alzheimer’s disease. *Journal of Gerontological Nursing,* *25*(6), 22–34. doi:10.3928/0098-9134-19990601-07 [Reasons for exclusion: no stranger comparison and non-inpatients.]

Stephenson, N.L.N., Swanson, M., Dalton, J., Keefe, F.J. & Engelke, M. (2007). Partner-delivered reflexology: Effects on cancer pain and anxiety. *Oncology Nursing Forum,* *341*(1), 127–132. doi:10.1188/07.ONF.127-132 [Reason for exclusion: no stranger comparison.]

Vahedian-Azimi, A., Ebadi, A., Asghari Jafarabadi, M., Saadat, S., & Ahmadi, F. (2014). Effect of massage therapy on vital signs and GCS scores of ICU patients: A randomized controlled clinical trial. *Trauma Monthly*, *19*(3), 19–25. doi:10.5812/traumamon.17031 [Reason for exclusion: no stranger comparison.]

Yousefi, H., Naderi, M. & Daryabeigi, R. (2015). The effect of sensory stimulation provided by family on arterial blood oxygen saturation in critical care patients. *Iranian Journal of Nursing and Midwifery Research,* *20*(1), 63–68. [Reason for exclusion: no stranger comparison.]
