# Supplementary material for: Interpersonal touch interventions for patients in intensive care: A design‐oriented realist review
Source: Nurs Open. 2018 Oct 24;6(2):216–35. doi: 10.1002/nop2.200 (PMC6419112; doi:10.1002/nop2.200)
Supplement: Supplementary file 7 [file NOP2-6-216-s007.docx]

**Appendix S7: Quality appraisal of included studies using the Mixed Methods Appraisal Tool**

**Table S7.1 Qualitative studies**

|  | **Quality assessment criteria** | | | | | |
| --- | --- | --- | --- | --- | --- | --- |
| **Reports** | **Clear research objectives** | **Collected data address the research question** | **Data sources relevant to address research objective** | **Process of analysing data relevant to address research objective** | **Appropriate consideration of how findings relate to context** | **Appropriate consideration of how findings relate to researchers’ influence** |
| ^a^Henricson (2008), Henricson et al. (2009) | Yes | Yes | Yes  Reasons for patients declining participation not reported. | Yes | Yes  Detailed description of intervention contexts, some description of interview context. | Yes |
| ^b^Martorella et al. (2014) | Yes | Yes | Yes  Most common reasons for declining participation reported. | Yes | Yes  Extremely detailed description of intervention context, limited description of interview context. | Can’t tell |

**Table S7.2 Quantitative randomised controlled (trials)**

|  | **Quality assessment criteria** | | | | | |
| --- | --- | --- | --- | --- | --- | --- |
| **Reports** | **Clear research objectives** | **Collected data address the research question** | **Clear description of randomisation** | **Clear description of allocation concealment and blinding** | **Complete outcome data (≥ 80%)** | **Low withdrawal**  **(≤ 20%)** |
| Adib-Hajbaghery et al. (2012, 2013, 2015) | Yes | Yes | No  Inconsistent reporting | Allocation: No  Blinding: No | Yes | Yes |
| ^a^Henricson (2008),  Henricson, Berglund, et al. (2008), Henricson, Ersson, et al. (2008) | Yes | Yes | Yes | Allocation: Yes  Blinding: None | Yes | No  40% |
| ^b^Boitor et al. (2015) | Yes | Yes | Yes | Allocation: Yes  Blinding: limited | No  30% for 3^rd^ intervention | Yes |
| Bagheri-Nesami et al. (2015) | Yes | Yes | No | Allocation: No  Blinding: No | Can’t tell | Can’t tell |
| Çınar (2008), Çınar Yücel & Eşer (2015) | Yes | Yes  Note delayed timing of outcome measures | No | Allocation: No  Blinding: limited | No  34% | No  66% |
| Ebadi et al. (2015) | Yes | Yes | Yes | Allocation: No  Blinding: limited | Yes | Yes |
| Korhan et al. (2014) | Yes | Yes | No | Allocation: No  Blinding: No | Yes | Yes |
| Olleveant (2003) | Yes | Yes | Yes | Allocation: Yes  Blinding: None | No: 70% for 1^st^ intervention;  21% for 2^nd^ intervention. | No  >40% at one month post ICU discharge |

**Table S7.2** *(Continued.)*

|  | **Quality assessment criteria** | | | | | |
| --- | --- | --- | --- | --- | --- | --- |
| **Reports** | **Clear research objectives** | **Collected data address the research question** | **Clear description of randomisation** | **Clear description of allocation concealment and blinding** | **Complete outcome data (≥ 80%)** | **Low withdrawal**  **(≤ 20%)** |
| Souri Lakie et al. (2012) | Yes | Yes | Yes | Allocation: Yes  Blinding: No | Can’t tell | Can’t tell |
| Tsay et al. (2005) | Yes | Yes  Note delayed timing of outcome measures | Yes | Allocation: No  Blinding: limited | Yes | Yes |
| Maa et al*.* (2013) | Yes | Yes | No | Allocation: Yes  Blinding: No | Yes | Yes |
| Yousefi, Naderi, & Daryabeigi (2015), Yousefi, Naderi, Daryabeigi, & Tajmiri (2015). | Yes | Yes | No | Allocation: No  Blinding: No | Can’t tell | Can’t tell |

**Table S7.3 Quantitative descriptive studies**

|  | **Quality assessment criteria** | | | | | |
| --- | --- | --- | --- | --- | --- | --- |
| **Reports** | **Clear research objectives** | **Collected data address the research question** | **Sampling strategy relevant to address research question** | **Sample representative of population under study** | **Appropriate measurements** | **Acceptable response rate**  **(≥ 60%)** |
| Kaur et al*.* (2012) | Yes | Yes | Can’t tell  Purposive sampling | Can’t tell | Can’t tell | Yes |

*Note.* Integration between quantitative and qualitative findings for the two combination-design studies^a&b^ was either not attempted or was extremely limited. We therefore did not categorize these studies as mixed method designs.

**References**

Adib-Hajbaghery, M., Abasi, A., Rajabi-Beheshtabad, R., & Azizi-Fini, I. (2012). The effects of massage therapy by the patient’s relative on vital signs of males admitted in critical care unit. *Nursing and Midwifery Studies*, *1*(1), 16–21. doi:10.5812/nms.7903

Adib-Hajbaghery, M., Rajabi-Beheshtabad, R., & Abasi, A. (2013). Effect of whole body massage by patient’s companion on the level of blood cortisol in coronary patients: a randomized controlled trial. *Nursing and Midwifery Studies*, *2*(3), 10–21. doi:10.5812/nms.13781

Adib-Hajbaghery, M., Rajabi-Beheshtabad, R., & Ardjmand, A. (2015). Comparing the effect of whole body massage by a specialist nurse and patients’ relatives on blood cortisol level in coronary patients. *ARYA Atherosclerosis, 11,* 126–132. Retrieved from http://arya.mui.ac.ir/index.php/arya/article/view/836/1380

Bagheri-Nesami, M., Gorji, M. A. H., Rezaie, S., Pouresmail, Z., & Cherati, J. Y. (2015). Effect of acupressure with valerian oil 2.5% on the quality and quantity of sleep in patients with acute coronary syndrome in a cardiac intensive care unit. *Journal of Traditional and Complementary Medicine, 5,* 241–247. doi:10.1016/j.jtcme.2014.11.005

Boitor, M., Martorella, G., Arbour, C., Michaud, C., & Gélinas, C. (2015). Evaluation of the preliminary effectiveness of hand massage therapy on postoperative pain of adults in the intensive care unit after cardiac surgery: A pilot randomized controlled trial. *Pain Management Nursing, 16,* 354–366. doi:10.1016/j.pmn.2014.08.014

Çınar, Ş. (2008). *The effect on anxiety and cost of hand massage and acupressure therapy in patients having mechanical ventilation support. Mekanik ventilasyon desteğinde olan hastalarda el masajı ve akupressur uygulamasının anksiyete ve maliyete etkisi.* (Doctoral dissertation, Ege University, Turkey). Retrieved from http://en.academicresearch.net/the-effect-on-anxiety-and-cost-of-hand-massage-and-acupressure-therapy-in-patients-having-mechanical-ventilation-support/

Çınar Yücel, Ş., & Eşer, İ. (2015). Effects of hand massage and acupressure therapy for mechanically ventilated patients. *Journal of Human Sciences, 12(2),* 881–896. doi:10.14687/ijhs.v12i2.3054

Ebadi, A., Kavei, P., Moradian, S. T., & Saeid, Y. (2015). The effect of foot reflexology on physiologic parameters and mechanical ventilation weaning time in patients undergoing open-heart surgery: A clinical trial study. *Complementary Therapies in Clinical Practice, 21,* 188–192. doi:10.1016/j.ctcp.2015.07.001

Henricson, M. (2008). *Tactile touch in intensive care: Nurses' preparation, patients' experiences and the effect on stress parameters.* (Doctoral dissertation, University of Borås, Sweden). Retrieved from http://hb.diva-portal.org/smash/get/diva2:876856/FULLTEXT01

Henricson, M., Berglund, A.-L., Määttä, S., Ekman, R., & Segesten, K. (2008). The outcome of tactile touch on oxytocin in intensive care patients: a randomised controlled trial. *Journal of Clinical Nursing, 17,* 2624–3633. doi:10.1111/j.1365-2702.2008.02324.x

Henricson, M., Ersson, A., Määttä, S., Segesten, K., & Berglund, A.-L. (2008). The outcome of tactile touch on stress parameters in intensive care: A randomized controlled trial. *Complementary Therapies in Clinical Practice, 14,* 244–254. doi:10.1016/j.ctcp.2008.03.003

Henricson, M., Segesten, K., Berglund, A.-L., & Määttä, S. (2009). Enjoying tactile touch and gaining hope when being cared for in intensive care—A phenomenological hermeneutical study. *Intensive and Critical Care Nursing, 25,* 323–331. doi:10.1016/j.iccn.2009.07.001

Kaur, J., Kaur, S., & Bhardwaj, N. (2012). Effect of ‘foot massage and reflexology’ on physiological parameters of critically ill patients. *Nursing and Midwifery Research Journal, 8,* 223–233. Retrieved from http://medind.nic.in/nad/t12/i3/nadt12i3c.shtml

Korhan, E. A., Khorshid, L., & Uyar, M. (2014). Reflexology: its effects on physiological anxiety signs and sedation needs*. Holistic Nursing Practice, 28,* 6–23. doi:10.1097/HNP.0000000000000007

Maa, S.-H., Wang, C.-H., Hsu, K.-H., Lin, H.-C., Yee, B., MacDonald, K., … Abraham, I. (2013). Acupressure improves the weaning indices of tidal volumes and rapid shallow breathing index in stable coma patients receiving mechanical ventilation: Randomized controlled trial. *Evidence-Based Complementary and Alternative Medicine, 2013,* 723128. doi:10.1155/2013/723128

Martorella, G., Boitor, M., Michaud, C., & Gélinas, C. (2014). Feasibility and acceptability of hand massage therapy for pain management of postoperative cardiac surgery patients in the intensive care unit. *Heart & Lung, 43,* 437–444. doi:10.1016/j.hrtlng.2014.06.047

Olleveant, N. A. (2003). *Physiological and psychological effects of aromatherapy massage on critically ill patients.* (Doctoral dissertation, University of Liverpool, U.K.). Retrieved from http://ethos.bl.uk/OrderDetails.do?uin=uk.bl.ethos.275050

Souri Lakie, A., Bolhasani, M., Nobahar, M., Fakhr Movahedi, A., & Mahmoudi, H. (2012). The effect of touch on the arterial blood oxygen saturation in agitated patients undergoing mechanical ventilation. *Iranian Journal of Critical Care Nursing, 5,* 125–132. Retrieved from http://en.journals.sid.ir/ViewPaper.aspx?ID=286124

Tsay, S.-L., Wang, J.-C., Lin, K.-C., & Chung, U.-L. (2005). Effects of acupressure therapy for patients having prolonged mechanical ventilation support. *Journal of Advanced Nursing, 52,* 142–150. doi:10.1111/j.1365-2648.2005.03576.x

Yousefi, H., Naderi, M., & Daryabeigi, R. (2015). The effect of sensory stimulation provided by family on arterial blood oxygen saturation in critical care patients. *Iranian Journal of Nursing and Midwifery Research, 20,* 63–68. Retrieved from http://ijnmr.mui.ac.ir/index.php/ijnmr/article/view/1123/832

Yousefi, H., Naderi, M., Daryabeigi, R., & Tajmiri, M. (2015). The effect of sensory stimulation provided by family on systolic and diastolic blood pressure and heart rate in critical care patients*. International Journal of Current Research, 7,* 11621–11626. Retrieved from http://www.journalcra.com/article/effect-sensory-stimulation-provided-family-systolic-and-diastolic-blood-pressure-and-heart-r
