# Supplementary material for: Interpersonal touch interventions for patients in intensive care: A design‐oriented realist review
Source: Nurs Open. 2018 Oct 24;6(2):216–35. doi: 10.1002/nop2.200 (PMC6419112; doi:10.1002/nop2.200)
Supplement: Supplementary file 8 [file NOP2-6-216-s008.docx]

**Appendix S8: Summary of papers informing theoretical framework**

| **Name and date** | **Paper type** | **Study design/intervention/theory/review topic** | **Key relevant findings/utility to review** |
| --- | --- | --- | --- |
| **Construction Principle 1. Dynamic touch may reduce stress and pain more effectively than static touch.** | | | |
| **1.1 Ascending inhibition of pain signals at the neural gate in the spinal cord.** | | | |
| Habig et al. (2017) | Empirical paper | The study aimed to explore the role of CT-afferents in pain modulation by investigating healthy volunteers and patients with small fibre neuropathy (SFN; a condition associated with damage to C-fibres) using fMRI & a pain visual analogue scale. Experiment one: heat pain & CT-targeted touch (applied with a soft brush) were applied separately and in combination to 20 SFN-patients & 20 healthy volunteers. Experiment two: investigated the effect of pain intensity on CT-induced pain modulation (10 healthy volunteers). | The study found that CT-stimulation significantly reduced heat pain in healthy volunteers but not in SFN-patients. Habig et al. concluded that the location of CT-induced pain reduction might be at the level of the dorsal horn since cortical activation for heat pain did not significantly differ with and without CT-targeted touch. |
| Ladak et al. (2014) | Review | The aim of the review was to determine the extent of inter-neural communications between peripheral nerve territories and their relationship to areas of adjacent and non-adjacent spinal or cranial nerves and axial lines (lines of discontinuity) in the upper and lower limbs, trunk and perineum, and head and neck regions. | Ladak et al.’s review found that nerve communications appeared consistently between contiguous sensory territories within areas of axial lines. Nerve communications also appeared to cross axial lines in the trunk & perineum. Further research is needed to resolve the ambiguities that remain in nerve communication and cutaneous nerve innervation. |
| Mancini et al. (2014) | Empirical paper | The study aimed to investigate the spatial organisation of touch–pain interactions within a single human dermatome. Two experiments tested how & where within a dermatome touch modulates the perception of laser-evoked pain. Experiment one (8 healthy volunteers). Experiment two (14 healthy volunteers). Tactile stimulation was applied with calibrated nylon filaments. Heat pain was measured using intensity ratings, qualitative descriptors, and signal detection measures of sensitivity and response bias. | The study found that touch produced a bias to judge laser stimuli as less painful. The bias decreased linearly when the distance between the laser and tactile stimuli increased. |
| Mancini et al. (2015) | Empirical paper | The study aimed to investigate whether touch can gate nociceptive input in conscious humans (21 healthy volunteers). Laser pulses were used to selectively activate Aδ and C-fibres in the epidermis. Von Frey hairs delivered tactile stimuli. Electroencephalogram recordings, laser blink reflex analysis (a marker of subcortical nociceptive processing at brainstem level), and three psychophysical measures (laser stimulus detection, qualitative descriptors, and numerical ratings of perceived pain) were employed. | The study found that touch suppressed the laser blink reflex and inhibited both Aδ and C-fibre laser-evoked potentials. Mancini et al. concluded that touch-induced analgesia is likely to be mediated by a subcortical gating of the ascending nociceptive input. Supraspinal mechanisms alone do not mediate touch-induced analgesia, as a number of previous studies have suggested. |
| Melzack & Wall (1965) | Theoretical paper | The gate control theory of pain. | The brain receives information about injury via a gate controlled system in the spinal cord which is influenced by a) nociceptive signals, b) other afferent signals, and c) descending (supraspinal) control. |
| **Name and date** | **Paper type** | **Study design/intervention/theory/review topic** | **Key relevant findings/utility to review** |
| **1.1 Ascending inhibition of pain signals at the neural gate in the spinal cord. (Continued).** | | | |
| Watanabe et al. (2015) | Empirical paper | The study aimed to investigate the types of cutaneous afferents and spinal opioid receptors that contribute to the antinociceptive effects of gentle microcone touch. Two experiments were conducted on 43 urethane-anaesthetised rats. Experiment one investigated differences in unitary activity of skin afferent fibres to touch using a microcone disc or a smooth disc (control), which were placed onto the skin of the inner thigh for 10 minutes. Unitary activity from cutaneous afferents was recorded from the saphenous nerve. Experiment two investigated the influence of spinal opioid receptors on the inhibitory effect of microcone touch on heart rate responses to noxious heat stimuli using intrathecal administration of naloxone, CTOP, and naltrindole (opioid receptor antagonists) and saline (control). Heat stimulation was applied to the skin of the lower back and rumps using a thermode. Heart rate responses were measured from blood pressure waveforms. | Microcone touch elicited greater discharge rates for Aδ and C afferents compared with the smooth disc. In contrast, for Aβ afferents, discharge rates were similar for both touch conditions. The magnitude of heart rate responses to heat-induced heart rate responses were significantly reduced by microcone touch in rats that received saline or naltrindole (δ-opioid receptor antagonist), whereas this effect was not observed in rats that received naloxone (non-selective opioid receptor antagonist) or CTOP (μ-opioid receptor antagonist).  Watanabe et al. concluded that excitation of Aδ and C afferents resulted in the release of μ-opioid ligands in the spinal cord, which in turn inhibited nociceptive transmission that contributes to sympathetic somatocardiac reflexes. |
| **1.2 Supraspinal mechanisms, including sensory, cognitive and affective processes, modulate pain transmission and subjective pain experience.** | | | |
| Melzack & Wall (1965) | Theoretical paper | The gate control theory of pain. | The brain receives information about injury via a gate controlled system in the spinal cord which is influenced by a) nociceptive signals, b) other afferent signals, and c) descending (supraspinal) control. |
| Bushnell et al. (2013) | Review | Review of the neural mechanisms underlying the modulation of pain by cognitive and emotional states | Different neural systems are involved in the attentional and emotional control of pain. Distraction can reduce pain perception. Negative emotional states may increase the unpleasantness of pain without altering pain intensity. |
| Melzack (2001) | Theoretical paper | The neuromatrix theory of pain. | Pain perception is produced by “neurosignature” patterns generated by a widely distributed neural network, the “neuromatrix”, the synaptic architecture of which is genetically programmed and modified by past experience. Output neurosignature patterns include pain perception, action programs, and stress regulation programs. Factors that contribute to output patterns generated by the neuromatrix include; cognitive-evaluative inputs, sensory-discriminative inputs, and motivational-affective inputs. |
| Melzack & Katz (2013) | Review | Review of past and present theories of pain including gate control theory and neuromatrix theory. | Establishes, in light of more recent evidence, the evolution and continued credibility and utility of gate control theory and neuromatrix theory. Psychological factors are considered an integral part of pain processing. |
| **Name and date** | **Paper type** | **Study design/intervention/theory/review topic** | **Key relevant findings/utility to review** |
| **1.3 The reward system, which comprises cortical and subcortical brain regions, is activated more strongly by gentle stroking movements vs. static touch.** | | | |
| Lindgren et al. (2012) | Empirical paper | An experimental study using fMRI to test the hypothesis that human touch combined with movement activates specific brain areas associated with pleasant sensations. Four touch conditions were employed on the forearm of healthy volunteers (N = 16): human touch (palm) with or without movement; and a human hand (palm) in a rubber glove, with or without movement. Each condition was repeated six times for each subject. Force (2.5 N) and velocity (1.5 cm/s) were measured indirectly and held constant across touch conditions. The pressure applied was considered likely to have activated Aβ afferents and CT afferents. Subjective pleasantness was measured using a visual analogue scale. | The study reported that subjective pleasantness ratings (N = 16) were significantly higher for the moving hand condition compared with the two rubber glove conditions, and the stationary human touch condition. fMRI findings indicated stronger activation of the pregenual anterior cingulate cortex (pgACC; a brain region previously associated with pleasant sensory stimuli) for the moving hand condition compared with the other three touch conditions. Stroking movements also activated the insula; this finding was consistent with the posterior insula functioning as a cortical target for CT afferents. Differences in temperature between touch applied directly through skin-to-skin contact (warmer) and touch applied with the rubber glove may at least in part, have contributed to the higher ratings of pleasantness and pgACC activation observed for the human hand condition. |
| **1.4 Reward reduces stress reactivity via endogenous opioid release.** | | | |
| Creswell et al. (2013) | Empirical paper | An experimental study to investigate a) whether a brief sexual reward activity (viewing erotic images of mixed sex couples) prior to an acute social stress challenge (the Trier Social Stress Test, TSST) would reduce cortisol reactivity to the stress challenge, and b) whether experiencing reward before the stress challenge would improve maths performance in the TSST. Fifty-four heterosexual men were randomly assigned to view reward images or neutral images. | The study reported that participants in the reward condition had significantly lower stress reactivity to the acute stress challenge, and showed non-significantly improved cognitive performance in the maths test.  Creswell et al. also summarise several lines of evidence from other studies support a reward-cortisol stress-buffering effect: a) reward increases endogenous opioid release, which may suppress HPA-axis activity; b) reciprocal links exist between stress and reward pathways; c) primary reward provides analgesic effects; d) rewarding environments can buffer HPA-axis activity to restraint stress in rodents. |
| Kaada & Torsteinbø (1989) | Empirical paper | A pre-post intervention study to investigate the effect of a thirty-minute connective tissue massage treatment on plasma β-endorphin level in 12 volunteers, most of who suffered from various types of pain. Blood was samples were taken for β-endorphin measurements before the massage, and at 5, 30 and 90 min after the massage. | The study reported that following the connective tissue massage, mean plasma β-endorphin levels showed a significant increase in concentration of 16%. Concentrations remained high at 30 min after the massage, and returned to baseline at 90 min. |
| **Name and date** | **Paper type** | **Study design/intervention/theory/review topic** | **Key relevant findings/utility to review** |
| **1.5 Pleasure-related analgesia.** | | | |
| Kut et al. (2011) | Empirical paper | A double-blind randomised controlled trial was used to investigate the role of endogenous opioid neurotransmission in pleasure-related analgesia. Naloxone, a non-selective opioid receptor antagonist, or saline was administered to 22 healthy volunteers to determine whether the reversal of opioidergic activity can attenuate the hedonic response to emotional pictures and reduce pleasure-induced analgesia. Analgesic effects were determined by measuring pain tolerance, pain ratings, autonomic reactivity (skin conductance level and startle blink electromyographic response). | The study reported that, contrary to expectations, hedonic responses to pleasurable stimuli, and altered pain tolerances were insensitive to naloxone, whereas subjective pain intensity and unpleasantness ratings increased following naloxone administration. Kut et al. concluded that, in addition to activating opioid-sensitive circuits, pleasure-related analgesia activates mainly opioid-insensitive pain-modulating circuits. |
| **1.6 CT afferents present in hairy skin respond optimally to warm, medium-velocity, gentle stroking.** | | | |
| Liljencrantz et al. (2017) | Empirical paper | A series of three experiments with a total of 44 healthy volunteers examined the contribution of CT afferents to pain modulation. Pain intensity was measured using a visual analogue scale. Experiment one compared the effect of concurrent application of slow brushing (CT optimal) or vibration on heat pain intensity applied to the thigh. Experiment two investigated the effect of slow brushing and heat pain stimuli applied separately to the forearm, and at varying intervals, with slow brushing applied over varying durations. The third experiment employed both slow and fast brushing immediately prior to heat pain, in addition, measures of state anxiety, state calmness, depression and alexithymia were obtained | The study reported that slow brushing was effective in reducing pain, whereas fast brushing or vibration were ineffective. Significant reductions in pain were observed for slow brushing (CT-optimal touch) when the brushing was applied both simultaneously and sequentially with heat pain stimuli. For sequential stimulation, effects were more pronounced when the interval between brushing and pain was shorter, and when pain was preceded by a longer duration of brushing. CT-related pain reduction was significantly associated with low state anxiety.  Liljencrantz et al. concluded that the mechanisms involved in CT-afferent activation induced analgesia may include spinal mechanisms and cortical mechanisms. It was considered unclear why Aβ preferential stimuli (i.e. fast brushing) were not observed to produce a gating effect on pain signalling. |
| von Mohr et al. (2017) | Empirical paper | An experimental study employing 84 female participants investigated the effects of slow-affective touch versus fast-neutral touch on feelings of ostracism induced by a social exclusion paradigm. Participants were randomly assigned to receive slow touch or fast touch. Tactile stimulation consisted of soft brush stroking on two regions of the participants forearm at either CT-optimal speed (3cm/s) or non CT-optimal speed (18cm/s). Participants were blindfolded during the tactile stimulation. Outcome measures included general affect (the Positive Affect and Negative Affect Schedule) and distress (the Need-Threat scale). After each trial, participants’ perceived pleasantness for CT-optimal and non CT-optimal touch was determined using a scale ranging from 0 to 100. | The investigators reported that induced distress was significantly lower in the slow-affective touch group compared with the fast-neutral touch group. In contrast, affective touch was not found to have a more general effect on post-exclusion affect.  Additionally, slow-affective touch was rated as significantly more pleasant than fast touch. |
| **Name and date** | **Paper type** | **Study design/intervention/theory/review topic** | **Key relevant findings/utility to review** |
| **1.6 CT afferents present in hairy skin respond optimally to warm, medium-velocity, gentle stroking. (Continued).** | | | |
| Vallbo et al. (2016) | Review | Review of the properties of CT afferents | Key properties of CT afferents determined by microneurography include optimal responsiveness to light indentation force (0.3–2.5 mN), intermediate velocity (1–10 cm/s), and warm (typical skin) temperature. Pleasantness ratings for tactile stimuli significantly correlate with mean impulse rate. |
| **1.7 Cortical networks associated with CT afferents include brain regions associated with positive affect.** | | | |
| Morrison (2016) | Review | Review summarising the major known cortical targets and wider networks associated with CT mechanoreceptive afferents | Through the use of neuroimaging techniques, the posterior insula has been identified as a major cortical target for CT afferents. The posterior insula is likely to play an important role in integrating affectively relevant somatic information, however, the hedonic experience of affective touch is likely to involve brain-wide networks that include reward-related networks. |
| **1.8 OT is released in response to CT afferent stimulation.** | | | |
| Walker et al. (2017) | Review | Review of a) the physiological effects of CT-targeted touch, and b) the reported effects of oxytocin, which are considered to mirror the effects of CT-targeted touch. | Walker et al. suggest that effects of CT-targeted touch include reduced physiological arousal, positive affect, and the inhibition of response to painful stimuli. These effects are considered to mirror effects reported for exogenous administration of oxytocin, and endogenous release of oxytocin. Thus, Walker et al. conclude that activation of CTs may trigger oxytocin release, although direct evidence to support this assertion is still required. |
| **1.9 OT modulates HPA-axis activity, increases reward processing, and reduces stress reactivity, fear and anxiety.** | | | |
| Cardoso et al. (2014) | Review | Meta-analytical review of the impact of intranasal oxytocin administration on cortisol levels during laboratory tasks. | After controlling for baseline differences in cortisol concentrations, moderation analyses indicated greater attenuation of cortisol response to laboratory tasks that strongly activated the HPA-axis, and in clinical populations (diagnosed with major depression, substance dependence, fragile X syndrome, borderline personality disorder) relative to healthy controls. |
| Sippel et al. (2017) | Review | An overview of evidence supporting the utility of oxytocin for treating major depressive disorders and posttraumatic stress disorder. Additionally, Sippel et al. review evidence for the behavioural effects of oxytocin and the potential neurobiological mechanisms and moderating contexts that may explain its behavioural effects. | Stimulated release of endogenous oxytocin in animals has been found to be associated with reductions in fear-related behaviour mediated by the amygdala.  Evidence from studies of intranasal oxytocin in humans suggest oxytocin may increase reward processing, reduce psychosocial stress reactivity, reduce fear and anxiety, and promote social cognition and behaviour. However, overall |
| **Name and date** | **Paper type** | **Study design/intervention/theory/review topic** | **Key relevant findings/utility to review** |
| **1.9 OT modulates HPA-axis activity, increases reward processing, and reduces stress reactivity, fear and anxiety. (Continued).** | | | |
| Sippel et al. (2017) (continued) |  |  | study findings provided mixed support for the positive effects of oxytocin. Prosocial behavioural effects may be facilitated by cues and contexts that are perceived as safe/positive.  Potential neurobiological mechanisms underlying the effects of oxytocin include mechanisms relating to the salience network, effects on neurotransmitter activity, attenuation of HPA-axis activity, and anti-inflammatory effects. |
| Walker et al. (2017) | Review | Review of a) the physiological effects of CT-targeted touch, and b) the reported effects of oxytocin, which are considered to mirror the effects of CT-targeted touch. | Evidence is presented that oxytocin facilitates social behaviour, enhances the salience of socially relevant sensory input, and reduces response to painful stimuli. Potential mechanisms underlying the effects of oxytocin include inhibition of the HPA-axis response to stressors, and modulation of parasympathetic activity via stimulation of the dorsal motor nucleus of the vagus nerve. |
| **1.10 OT promotes prosocial effects.** | | | |
| De Dreu et al. (2011) | Empirical paper | A series of five experiments, which employed a double-blind, randomized controlled trial design, to investigate the effect of intranasal oxytocin administration on intergroup bias in indigenous Dutch male volunteers (*N* = 280). Experiments one and two used the Implicit Association Test, which assesses social valuation, to determine in-group favouritism and out-group derogation. Experiment two used to the infrahumanization task to assess infrahumanization (the tendency to preferentially associate in-group members with secondary emotions that are frequently perceived as uniquely human). Experiments four and five investigated behavioural intergroup biases using the Moral Choice Dilemma Task. | Results suggested that oxytocin creates intergroup bias by motivating in-group favouritism and, in some cases, out-group derogation.  The effects of oxytocin in promoting trust, empathy, and prosocial behaviours may be limited to individuals belonging to one’s in-group. |
| Piva & Chang (2018) | Review/theory paper | Piva & Chang review three divergent prominent hypotheses that seek to explain the impact of oxytocin on social cognition, namely the prosocial hypothesis, the social salience hypothesis, and the approach/withdrawal hypothesis. Further, the authors propose a unified framework encompassing the similarities and meaningful differences between existing theories. The plausibility of the proposed multistage framework is evaluated with reference to mechanistic research on the effects of oxytocin within the CNS across multiple species including rodents, non-human primates and humans. | Early studies on the effects of oxytocin indicated nearly universal prosocial effects on multiple forms of behaviour. However, it has become clear that effects of oxytocin are context-sensitive; in certain contexts prosocial effects may be abolished, and accumulating evidence indicates that oxytocin often elicits antisocial effects, including envy, aggression, and decreased trust. In order to account for context-specific effects of oxytocin, researchers have proposed that oxytocin may facilitate recognition and attention to external social cues (the social salience hypothesis), and distinctly, in the approach/withdrawal hypothesis, that oxytocin enhances approach behaviours and decreases withdrawal behaviours. |
| **Name and date** | **Paper type** | **Study design/intervention/theory/review topic** | **Key relevant findings/utility to review** |
| **1.10 OT promotes prosocial effects. (Continued).** | | | |
| Piva & Chang (in press) (continued) |  |  | Still these more recent hypotheses are unable to accommodate the results of all past studies. These remaining inconsistencies lead Piva & Chang to propose a new unified hypothesis in which the effects of oxytocin on social decision-making are conceptualised via a five-stage model. Sequential stages in this model comprise: sensory input, sensory perception, valuation, decision formulation, and behavioural output. Potential feedback effects may occur between individual stages. Oxytocin is proposed to act on or gate any of the sequential processes, with modulation of individual stages depending on external context and individual differences. Mechanistic findings suggest oxytocin may act on sensory and reward areas within the CNS, potentially implicating oxytocin at multiple stages of social decision-making. |
| **Construction Principle 2. Lightening sedation may promote touch-mediated reductions in stress.** | | | |
| **2.1 Increased cortical activity and connectivity promotes reward processing and social cognition.** | | | |
| Macdonald et al. (2015) | Review | Review of findings from functional neuroimaging studies that have used anaesthetic drugs to study cognition at different levels of consciousness. | Anaesthetic drugs have been reported to reduce cortical activity and connectivity (including subcorticocortical connectivity) in higher order brain networks, including the salience network and the default-mode network, which couples with reward processing regions (Gerlach et al., 2014). Cortical activity may vary as a function of the nature or intensity of the tactile stimulus. |
| **2.2 Optimizing opioid administration (avoiding oversedation) may promote social comfort seeking.** | | | |
| Loseth et al. (2014) | Review/theory paper | Review of evidence for µ-opiod mediation of reward processing, emotional regulation, and affiliation in humans, non-human primates, rodents and other species.  Based on evidence from psychopharmacological studies in rats and non-human primates, Loseth et al. introduce the State dependent µ-Opiod Modulation of SOcial Motivation (SOMSOM) model. | Loseth et al. propose that for mammalian species in a distress state ‘social contact is sought out for comfort and relief. Social comfort reduces distress at least in part via µ-opioid receptor (MOR) activation.’ (p. 11). Activation of MOR by exogenous MOR agonists (e.g. morphine) provides relief and diminishes the need for social approach behaviours. In contrast, for mammalian species in comfort states, MOR agonists promote increased appetitive motivation and social exploration. |
| **Name and date** | **Paper type** | **Study design/intervention/theory/review topic** | **Key relevant findings/utility to review** |
| **2.3 Optimizing opioid administration (avoiding oversedation) may promote reward responding.** | | | |
| Case et al. (2016) | Empirical paper | Experimental study to investigate the effect of opioid blockade on perceived pleasantness and intensity of touch. Healthy individuals (n = 28) and chronic pain patients diagnosed with fibromyalgia (n = 24) received slow and fast brushing stimuli on the forearm both before and after intravenous administration of naloxone or saline. Touch pleasantness was measured using a visual analogue scale.  As part of an unrelated fMRI study, participants were received painful heat stimuli before and during the drug infusion and were isolated in an MRI scanner. Participants were therefore likely to have experienced a state of stress. | In healthy individuals, opioid blockade resulted in a marginally significant increase in ratings of touch pleasantness, whereas for chronic pain patients opioid blockade showed no effect on touch pleasantness.  Case et al. suggest that ‘Baseline stress may have caused opioid blockade to increase distress and heighten the social reward of affective touch. This interpretation suggests that opioids influence the motivational state that determines the reward and pleasantness of social touch’ (p. 8). Findings correspond with the SOMSOM model (Loseth et al., 2014; see above).  A corollary to Case et al.’s findings is that higher occupancy of opioid receptor sites by exogenous opioid receptor agonists may reduce the perceived pleasantness of interpersonal touch. Interestingly, Case et al.’s conclusions appear to contradict suggestions by Bogdan and Pizzagalli (2006) that reward responding may be inhibited by acute stress. Possibly, these differences may be explained by differences in baseline stress, differences in the nature and percieved severity of the stressor, differences in the nature of the reward, and/or differences in measurement techniques used to assess reward responsiveness. |
| Nummenmaa et al. (2016) | Empirical paper | Experimental study to investigate whether social touch modulates opioidergic activation in humans. Participants were 18 young healthy male volunteers and their female romantic partners. Each male participant was scanned twice using positron emission tomography (PET). MOR (µ-opiod receptor) availability was quantified with the MOR-specific ligand [11C]carfentanil. Two experimental conditions were employed: 1) the social touch challenge, in which male participants were touched by their romantic partners before and during the scan; and 2) a control condition, in which participants lay alone in the scanner. Participants’ perceptions of pleasure, pain, arousal, tension, and sleepiness were measured using a visual analogue scale at the beginning, midpoint and end of each PET scan. Cortisol levels were measured using venous blood samples taken at the beginning and midpoint of each scan. Relationship dependent effects of social touching were determined after the scan; blindfolded participants were touched on the leg by their partner, however, the | The study reported that participants experienced significantly more pleasure during the social touch condition compared with the no-touch condition. Participants also reported relatively lower pain, less tension, and increased sleepiness during the touch condition. Cortisol levels showed no difference between the two conditions. In the post scan test, participants rated touch by their partner as significantly more pleasurable than touch by a stranger.  Whole-brain analysis of PET data showed that MOR availability was significantly higher during the touch condition versus the no-touch condition, suggesting that social touch lowered endogenous µ-opioid activity. Effects were observed most strongly in reward circuitry, but also in regions involved in emotional and social functions. |
| **Name and date** | **Paper type** | **Study design/intervention/theory/review topic** | **Key relevant findings/utility to review** |
| **2.3 Optimizing opioid administration (avoiding oversedation) may promote reward responding. (Continued)** | | | |
| Nummenmaa et al. (2016) (Continued). |  | participants were led to believe that on half of the occasions, the touch was provided by a male stranger. | The results of the study are interpreted by Nummenmaa et al. as suggesting that by reducing stress social touch may “downregulate the opioidergic component of physical pain and social distress circuitries, thus leading to decreased tonic opioid neurotransmitter release […]. Theoretically, this reduction in the basal state of the MOR system might promote more effective coding of the pleasurable sensations associated with […] social touching”. (p. 245).  The results of this study appear to contradict suggestions by Loseth et al. (above) that social comfort is in part mediated by MOR activation. |
| **Construction Principle 3. Touch provided by a familiar conspecific may promote stress reduction** | | | |
| **3.1 Psychosocial resources are construed as bioenergetics resources. Conflation of self and others results in a diminished perception of threat. Proximal mediators may include OT and endogenous opioids.** | | | |
| Beckes & Coan (2011) | Theory paper | Social baseline theory | Beckes and Coan propose that social proximity decreases important and often metabolically costly neural (e.g. prefrontal cortex) and somatic resources through the processes of risk distribution and load sharing. Load sharing is a function of trust and interdependence. Neural circuits associated with the regulation of emotions are less active when social support is provided. Other authors have suggested that the down-regulation of negative affect may be mediated by oxytocin, endogenous opioids, tonic changes to neurotransmitter fibre systems, or dopaminergic systems. |
| Coan & Sbarra (2015) | Theory paper | Social baseline theory | Coan and Sbarra propose that the brain expects that the proximity of relational partners mitigates risk and reduces the level of effort required to meet a variety of goals. A key mechanism by which this is achieved is by the incorporation of relational partners into neural representations of the self. Effects are potentiated in the context of higher relationship quality, intimacy, and higher perceived mutuality. |
| Coan et al. (2006) | Empirical paper | Experimental study to investigate the neural response of married women subjected to the threat of electric shock while holding their husband’s hand, the hand of a stranger, or no hand. fMRI and questionnaire. Healthy married couples (n = 16) | Neural activation to threat and subjective reports of unpleasantness were significantly lower in the spousal-touch condition than in the stranger-touch condition. The effect of spousal hand-holding on neural activation to threat in the right anterior insula, superior frontal gyrus and hypothalamus correlated negatively with the wives’ perceptions of marital quality. |
| **Name and date** | **Paper type** | **Study design/intervention/theory/review topic** | **Key relevant findings/utility to review** |
| **3.2 The communication of positive emotions, such as love, via touch.** | | | |
| Hertenstein, Keltner, et al. (2006) | Empirical paper | Experimental study to investigate whether people can identify emotions from the experience of being touched on the arm (without seeing the touch). Healthy individuals (n = 212 and n= 58). | Participants in the United States and Spain could decode anger, fear, love, gratitude, and sympathy at much-better-than-chance levels. |
| Hertenstein, Verkamp, et al. (2006). | Review | Review of the communication function served by touch in humans, non-human primates, and rats. | Touch can communicate and generate positive emotions and modulate negative emotions. |
| **3.3 Familiar conspecifics express empathy by providing more attuned and rewarding touch. Feeling understood activates the reward system.** | | | |
| Goldstein et al. (2016) | Empirical paper | Experimental study to investigate the analgesic effects of social touch (hand holding) for heat pain. Questionnaires included the Interpersonal Reactivity Index, which measures empathic capacity. Dyads of healthy individuals (n = 23 and n = 20); women and their romantic partners or strangers. | Greater analgesic effects were reported for the partner-touch condition compared with partner-no touch, stranger-touch, and pain-alone conditions. The level of the analgesic effect was found to be associated with the toucher’s empathy only during the touch condition. Goldstein et al. suggest that ‘...couples who experience more empathy towards each other, compared with strangers, may provide a more attuned and rewarding touch that may in turn increase the analgesic effect of touch’ (p. 1050). |
| Morelli et al. (2014) | Empirical paper | Experimental study to investigate the effects of experimentally induced felt understanding and not understanding. fMRI and questionnaire. Healthy individuals (n = 19). | fMRI results demonstrated that feeling understood activated neural regions associated with reward and social connection (ventral striatum and middle insula). Questionnaire results paralleled the fMRI results. |
| **Construction Principle 4. Treatment repetition may provide cumulative benefits** | | | |
| **4.1 Stress reduction improves functionality of reward related neural circuitry.** | | | |
| Bogdan & Pizzagalli (2006) | Empirical paper | Experimental study to investigate if acute stress impairs reward responsiveness. Reward responsiveness (an empirical measure of hedonic capacity) was measured for 80 healthy female volunteers in the context of experimentally manipulated stress (threat of electric shock or negative performance feedback) conditions and a no-stress condition. Reward responsiveness was assessed by ability to modulate behaviour as a function of past reward (visual reward feedback “Correct!! You won 5 cents” following the participants’ correct identification of visual images). Negative affect and anxiety were measured using the state form of the Spielberger Trait Anxiety Inventory (STAI) and the Positive and Negative Affect Scale (PANAS). | Findings indicated that acute stress reduced reward responsiveness, particularly for the threat-of-shock condition.  Participants reporting greater anhedonic symptoms in their daily life displayed the greatest stress-induced hedonic deficits. |
| Pizzagalli (2014) | Review/theoretical paper | Review of the role of anhedonia, dopamine, and stress in depression, and the effects of stress on dopaminergic pathways. A model is presented postulating that anhedonia results from dysfunctional interactions between stress and brain reward systems. | Multiple lines of evidence were found to suggest that chronic stressors increase risk of depression by reducing hedonic capacity, incentive motivation, and reinforcement learning. |
| **Name and date** | **Paper type** | **Study design/intervention/theory/review topic** | **Key relevant findings/utility to review** |
| **4.2 Increased familiarity with the intervention may reduce stress by virtue of knowing what to expect.** | | | |
| de Berker et al. (2016) | Empirical paper | An experimental study to investigate the effects of different forms of uncertainty on subjective and physiological stress responses to electric shock. Forty-five healthy volunteers completed a set of 320 trials. For each trial participants were presented with one of two stimuli, and were asked to predict which outcome was likely to follow (snake or not snake). One the decision had been made, the outcome was displayed, and in the case of the snake stimulus, outcome presentation was coincident with an electric shock. Each session of 320 trials was divided into 10 blocks of different stimulus-outcome probabilities, varying from heavily biased (90/10) to unbiased (50/50). Outcome measures included subjective pain ratings, skin conductance, pupil diameter, and irreducible uncertainty. Results for learning and stress responses were analysed using multiple types of models including a Bayesian learning model, regression models, and linear models. | Results revealed that beliefs about uncertainty mediated the strength of participants’ stress responses. Subjective stress, pupil diameter and skin conductance all tracked internal estimates of irreducible uncertainty. Stress responses were greatest when outcome probabilities for electric shock were unbiased (50/50), compared with outcome probabilities that were heavily biased (90/10 and 10/90). Additionally, individual reporting high levels of chronic stress (life stress, measured using the Perceived Stress Scale) were found to behave as if they believed environment was more uncertain. |
| Peters et al. (2017) | Theoretical paper | Peters et al. present an “information-theoretic” account of stress and “allostatic load” employing the recent developments in theoretical neurobiology including the “free energy principle”. | The essence of stress is proposed to be uncertainty, also conceptualised as entropy or “expected surprise”. The free energy principle is based on the assertion that biological agents strive to minimize their uncertainty or the “variational free energy” about future outcomes. If uncertainty cannot be reduced by cognitive efforts, a persistent cerebral energy crisis may develop, creating allostatic load that contributes towards systemic and brain malfunction. The authors discuss the strategies the brain uses to resolve uncertainty. Individuals show two genetically predisposed response patterns to stress: as a consequence of an alleviated allostatic load “habituators” are better able to cope with uncertainty than “non-habituators”. Also, in patients receiving therapeutic drugs that interfere with cerebral mechanisms for mastering uncertainty, while a temporary sense of well-being may be achieved, the updating of his/her internal Bayesian models of the world is suspended. |
| **4.3 Positive neural interactions between reward components “liking”, “wanting” and “learning” including cognitive and unconscious processes.** | | | |
| Berridge & Robinson (2003) | Review | Review of the psychological components of reward, their interactions, and brain substrates. | Major components of reward include learning, affect, and motivation. Each major component is composed of conscious and unconscious psychological component processes. Components interact and may reinforce one another. |
| **Name and date** | **Paper type** | **Study design/intervention/theory/review topic** | **Key relevant findings/utility to review** |
| **4.4 Lower levels of anxiety and psychological stress reduce chronic pain perception via modulation of the neurosignature pattern.** | | | |
| Melzack (2001) | Theoretical paper | The neuromatrix theory of pain. | Pain perception is produced by “neurosignature” patterns generated by a widely distributed neural network, the “neuromatrix”. Output neurosignature patterns are influenced not only by somatic sensory input, but also by cognitive and affective inputs including psychological stressors, thus explaining the frequent co-occurrence of chronic stress and chronic pain. |
| **4.5 Reduced perception of pain reduces pain anxiety and pain catastrophizing.** | | | |
| McCracken et al. (1992) | Methods paper | Describes the development and validation of the Pain Anxiety Symptom Scale (PASS), to measure fear of pain. The test was administered to 104 referrals to a multidisciplinary pain clinic. | Pain experience may result in fearful thoughts about pain, physiological symptoms of fear, and avoidance and escape of activities that are believed to increase pain. These processes may be dysfunctional and may increase the affective component of pain. |
| Sullivan et al. (1995) | Methods paper | Describes the development and validation of the Pain Catastrophizing Scale (PCS). The test was administered to 425 undergraduates. | Results for PCS scores were found to be comprised of three components; rumination, magnification, and helplessness. Participants subjected to a cold pressor test or an aversive electrodiagnostic procedure, catastrophizers reported more negative pain-related thoughts, greater distress, and greater pain intensity than non-catastrophizers. |
| **4.6 Emergent processes: Positive interactions between reduced psychological stress and reduced pain perception.** | | | |
| Sharp & Harvey (2001) | Review/ theoretical paper | A review of chronic pain and posttraumatic stress disorder (PTSD) that covers symptoms, prevalence, comorbidity, explanatory psychological theories, and potential factors that maintain the two disorders.  The mutual maintenance model of chronic pain and PTSD. | Results from a number of studies suggest a correlation between PTSD and chronic pain. Seven specific mechanisms are identified by which the two conditions may be mutually maintaining. These mechanisms are specified as follows:  1. PTSD patients may have an attentional bias towards pain sensations that amplifies pain sensations. 2. Anxiety sensitivity tendency in chronic pain and PTSD patients promotes misinterpretation and catastrophization of somatic sensations associated with pain and arousal. 3. Pain may serve as a reminder of trauma, triggering an arousal response, which in turn may lead to avoidance of sensations associated with trauma producing escalating levels of distress and disability. 4. Avoidant coping styles may lead to physical deconditioning and prevent activation and resolution of the fear network. 5. Depression, lethargy, and reduced activity levels associate with chronic pain and PTSD may lead to increased disability and inhibit processes necessary for |
| **Name and date** | **Paper type** | **Study design/intervention/theory/review topic** | **Key relevant findings/utility to review** |
| **4.7 Emergent processes: Positive interactions between reduced psychological stress and reduced pain perception. (Continued).** | | | |
| Sharp & Harvey (2001) (continued) |  |  | trauma resolution. 5. Pain perception is increased by elevated anxiety, which may lead to reduced activity and increase disability and distress. 6. The high levels of cognitive activity associated with chronic pain and PTSD, limit cognitive capacity to employ adaptive cognitive strategies to reduce perception of pain.  Maladaptive mechanisms described by Sharp and Harvey may be transferable to the context of ICU. Patient may also have pre-existing PTSD. By reducing psychological stress and pain, and promoting attentional capacity, interpersonal touch interventions, employed in conjunction with other therapeutic strategies, may help break the cycle of interactions involved in the maintenance of pain and distress. |
| **Construction Principle 5. Interventions that provide frequent episodes of moderate intensity positive affect (PA) may provide greater long-term stress reduction compared with interventions that provide infrequent episodes of high intensity PA.** | | | |
| **5.1 Psychological and physiological costs associated with high intensity PA affect are avoided.** | | | |
| Diener, Colvin, et al. (1991) | Empirical paper | Five studies were conducted on undergraduate students to explore the relationship between PA and negative affect (NA). Two studies investigated cognitive amplifying and damping processes, studies one and two (*N* = 189 and 109 respectively) investigated how context influenced judgements, study three (*N* = 98) investigated how context influences judgments, study four (*N* = 22) asked participants to recall the contexts that preceded their past happiest times, and study five (*N* = 192) investigated whether people’s desire for a particular outcome influenced their level of affect. | Results from studies one and two supported the hypothesis that an amplifying cognitive strategy would produce more extreme PA and NA rating compared to a damping cognitive strategy. Results from study three suggested that an extremely positive event might lower the value of subsequent moderately positive events. Results from study four suggested that events experienced as extremely happy were more likely to occur following negative events. Results form study four suggested that when future positive or negative outcomes were strongly valenced by participants, the subsequent events were experienced more intensely, although this was statistically significant only for negative events. |
| Human et al. (2015) | Empirical paper | Two studies were conducted to investigate the whether PA variability was associated with HPA (hypothalamic-pituitary-adrenal) axis functioning by measuring daily salivary cortisol profiles. It was hypothesised that too much or too little PA variability may be maladaptive and associated with less favourable cortisol profiles, whereas moderate PA variability may be optimal and associated with more favourable cortisol profiles. | Across the two studies it was found that moderate PA variability was associated with more favourable cortisol profiles, including lower cortisol levels and steeper and more curvilinear slopes. This finding applied for both groups, both within and across days. The nonlinear associations between PA and cortisol measures for both studies suggested that moderate PA variability may be optimal, reflecting a balance between adaptive flexibility and rigidity, but very high or very |
| **Name and date** | **Paper type** | **Study design/intervention/theory/review topic** | **Key relevant findings/utility to review** |
| **5.1 Psychic and physiological costs associated with high intensity positive affect are avoided. (Continued).** | | | |
| Human et al. (2015)  (continued) | Empirical paper | Study one investigated PA variability within and across days for 104 healthy middle-aged adults recruited from Berlin, Germany. PA and salivary cortisol were measured six times per day for six consecutive days. PA was measured using three items from the Multidimensional Affect Scale. PA variability was calculated using intraindividual standard deviations (iSD)  Study two investigated PA variability across six consecutive weeks in 88 community dwelling older adults (65–85 years old) recruited from Vancouver, British Columbia. PA and salivary cortisol were measured one day per week across six weeks, and involved an experimental manipulation in which participants were randomly assigned to spend a small windfall of money on themselves or others for three weeks over the six-week period. Salivary samples were taken one day per week (four times per day) for six consecutive weeks, PA was measured prior to salivary sampling days using the Positive and Negative Affect Schedule (PANAS). PA variability was calculated using iSD. | low variability may be maladaptive.  In addition, differences were reported between studies: In Study one, high PA variability was associated with least favourable cortisol profiles, whereas in Study two, low levels of PA variability were associated with least favourable cortisol profiles. Suggested possible explanations for these differences were: 1. Age-related processes, timescale of PA variability, and cultural differences.  It is uncertain to what extent these findings may apply to experimentally induced PA. |
| Parducci (1968) | Empirical/theoretical paper | A series of experiments were conducted on undergraduate students to investigated the influence of judgments of personal satisfaction depend on the context within which the judgements are made. The experiments were designed to test Parducci’s range-frequency theory (cognitive psychology). For example: one experiment investigated the influence of moral judgment of acts of wrongdoing in the context of “mild” or “nastier” acts; another experiment investigated the influence of average card set value on satisfaction with individual winnings from a card game; and a third experiment investigated the influence of juxtaposing numerals with numeral of smaller or greater value on participants’ judgement of the size of individual numerals. | Results from the series of experiments suggested that individual judgements were influenced by the range and frequency of stimuli within which the individual stimuli were situated. For example, results from one experiment suggested that participants were more lenient in their moral judgments in the context of “mild” acts of wrongdoing. Results from another experiment suggested that satisfaction with individual winnings was higher in the context of a lower value card set. Results from a third experiment suggested that judgements of size of numeral were more extreme when numerals were juxtaposed with numerals of contrasting size. |
| Parducci (1984) | Theoretical paper | Range-frequency theory is applied to the experience of happiness. | Parducci proposes that the experienced value of an event depends on comparison with other events. As summarised by Diener, Colvin et al. (1991), Parducci’s range-frequency theory (1968, 1984) states that two processes are involved in any judgment of happiness: The range principle proposes that if a positive event occurs near the top of a contextual range of positive–negative events (absolute position) it will be judged more positively than if the same event occurs near the bottom of a contextual range. Thus, if an extremely positive event extends the contextual range, other events may be judged more negatively. The frequency principle proposes that an event will be judged positively if it is judged to be |
| **Name and date** | **Paper type** | **Study design/intervention/theory/review topic** | **Key relevant findings/utility to review** |
| **5.1 Psychic and physiological costs associated with high intensity positive affect are avoided. (Continued).** | | | |
| Parducci (1984)  (continued) |  |  | above average (relative position). Judgments are proposed to reflect a compromise between these two principles. |
| Pressman & Cohen (2005) | Theoretical paper | Two alternative models are presented to explain the influence of PA on health: a) the main (direct) effects model and b) the stress-buffering model. | *The main (direct) effects model.* PA may directly influence health via: changes in health practices; autonomic nervous system activation; hypothalamic–pituitary–adrenal axis activation (may be mediated by oxytocin); endogenous opioids; and social factors. Additionally, high PA states can trigger short-term increases in physiological arousal that may be associated with potentially harmful effects on immune, cardiovascular, and pulmonary systems.  *The stress-buffering model of PA influences on health.* The influence of PA on health may occur primarily through buffer against potentially pathogenic influences of stressful events. Positive emotions promote building social, psychological, intellectual, and physical resources, which may be drawn upon when facing potential or actual stressful events. Positive emotions may also promote restorative activities, which may reduce stress appraisals and negative affective responses to stress, and promote stress recovery.  Pressman and Cohen also speculate that “It is possible that there is a curvilinear relationship between PA and health with risk decreasing as one moves from low to moderately high levels of PA, but increasing as one reaches extremely high levels” (p. 961). |
| Solomon (1980) | Theoretical paper | The opponent-process theory of acquired motivation. | Three affective phenomena are often observed when an individual is presented with an affective stimulus: a) Affective contrast, for example, the termination of an extremely positive event may result in an unpleasant hedonic state. b) Hedonic habituation may occur following frequent repetition of the stimulus. c) After frequent repetition of a stimulus, a withdrawal syndrome may emerge directly following stimulus termination. |
| **5.2 Patients experience a higher relative frequency of positive affect versus negative affect.** | | | |
| Blevins et al. (2017) | Empirical paper | An epidemiological study to investigate the influence of PA on levels of inflammation. Publically available data for perceived psychological stress (PPS), frequency of PA, frequency of NA (questionnaire data) and C-reactive protein (an indicator of systemic inflammation) for 4543 participants. Two hierarchical moderated regression models (unadjusted and adjusted) were used to test the hypothesis that PA would moderate the relationship between PPS and CRP. | Results suggested that under conditions of increased PPS, higher frequency of PA buffered against elevated systemic inflammation. |
| **Name and date** | **Paper type** | **Study design/intervention/theory/review topic** | **Key relevant findings/utility to review** |
| **5.2 Patients experience a higher relative frequency of positive affect versus negative affect. (Continued)** | | | |
| Diener, Sandvik, et al. (1991) | Theoretical paper | It is argued that happiness researchers should assess primarily the relative frequency of positive versus negative affect. | Diener, Sandvik et al. propose that affective well-being is equated with the frequency, and not the intensity, of positive versus negative affect. In addition, it is proposed that intense positive experiences can cause subsequent events and situations to be experienced less positively. Interventions that aim at increasing long-term happiness should focus on increasing the frequency and duration of happy experiences. |
| **5.3 Frequent treatment repetition facilitates estimates of predictability, which reduces cerebral energy demands & stress response.** | | | |
| de Berker et al. (2016) | Empirical paper | See 4.2 above. | See 4.2 above.  Patients in intensive care may find it easier to predict the delivery of interventions if they are provided more frequently, particularly if they are experiencing temporal disorientation. |
| Peters et al. (2017) | Theoretical paper | See 4.2 above. | See 4.2 above. |
| **5.4 Promotion of anticipatory pleasure.** | | | |
| Gooding & Pflum (2014) | Methods paper | Describes the validation of a new measure, the Anticipatory and Consummatory Interpersonal Pleasure Scale (ACIPS), designed to assess pleasure for social an interpersonal interactions. Participants comprised 575 undergraduate students. | Preliminary testing on a non-clinical sample demonstrated the ACIPS is a reliable and valid method of assessing hedonic capacity for social and interpersonal pleasure. Anticipatory pleasure items were found to correlate with consummatory items. The authors note that results from other studies suggest that schizophrenia patients may have reduced anticipatory pleasure but intact consummatory pleasure.  Note. Link between anticipatory pleasure and reward processing (Berridge & Robinson, 2003; see section 4.3). |
| Gard et al. (2006) | Methods paper | Describes the development and validation of the Temporal Experience of Pleasure Scale (TEMPS), a scale designed to measure individual trait dispositions for both anticipatory and consummatory experiences of pleasure. Scale construction and validation employed five large samples (100–755) of undergraduate students. | Research from the fields of neuroscience, social psychology, and clinical psychology support the view that distinct processes are responsible for anticipatory and consummatory pleasure. Results indicated that the anticipatory component related to reward responsiveness and imagery, while the consummatory component related to openness to different experiences, and appreciation of positive stimuli.  Gard et al. reported that deficits in anticipatory pleasure mildly linked to depression, whereas consummatory pleasure was found to be unrelated to depression. Other authors have noted the importance of anticipatory pleasure in relation to personality, especially extraversion. |
| **Name and date** | **Paper type** | **Study design/intervention/theory/review topic** | **Key relevant findings/utility to review** |
| **Construction Principle 6. Moderate pressure touch may reduce stress more directly compared with light pressure touch** | | | |
| **6.1 Moderate pressure stimulates dermal and subdermal pressure receptors activating structures within the autonomic nervous system.** | | | |
| Field (2016) | Review | Review of the effects of moderate pressure massage and potential underlying mechanisms. | Field proposes that reported increases in vagal activity after moderate pressure massage may be explained by the stimulation of dermal and subdermal pressure receptors that signal to the limbic system including hypothalamic structures involved in autonomic system regulation and cortisol secretion. |
| Field et al. (2010) | Review | Review of the effects of moderate pressure massage. Field et al. state that ‘Moderate pressure is essential for massage therapy effects.’ | Field et al. propose that the diverse benefits of massage therapy, including psychological outcomes and neuroendocrine function, rely on the use of moderate pressure to activate a mechanism involving increased vagal activity and ‘may be mediated by the stimulation of dermal and /or subdermal pressure receptors that are innervated by vagal afferent fibres, which ultimately project to structures involved in autonomic nervous system regulation.’ (p. 384). |
| **6.2 Mechanical pressure stimulation of mechanoreceptors in skeletal muscle elicits a reflexive autonomic nervous system response.** | | | |
| Watanabe & Hotta (2017) | Empirical paper | Experimental study to investigate the contribution of cardiac autonomic nerves to heart rate responses induced by mechanical pressure stimulation of skeletal muscles. Mechanical pressure was applied either to calf muscles (perpendicular pressure applied to the overlying skin, n = 9; and directly to muscle, n = 3) or to the hind paw (pinch stimulation) in isoflurane-anaesthetized and mechanically ventilated rats. To identify the autonomic nerve pathway involved, the vagus nerve and cardiac sympathetic nerves were surgically severed, or for one rat, vagus efferents were pharmacologically blocked. | Pressure stimulation of overlying skin and direct stimulation of muscle resulted in slightly tachycardic response for relatively low pre-stimulus HRs, and more substantial bradycardic response for relatively high pre-stimulus HRs. Results for the autonomic nerve block conditions suggested that the cardiac sympathetic nerves, and not the vagus nerve were responsible for the observed effect. Decreases in cardiac sympathetic efferent nerve activity were associated with decreases in heart rate. Watanabe and Hotta suggested that in the unanaesthetized condition the vagus nerve may contribute more to HR changes because cardiac vagus nerve is more sensitive to anaesthetics than sympathetic nerve. |
| **6.3 Arterial baroreceptors transmit information about cardiovascular arousal to brain regions implicated in affective & cognitive processing.** | | | |
| Garfinkel & Critchley (2016) | Opinion | Garfinkel and Critchley focus on how cardiovascular arousal levels (how quickly and strongly the heart is beating) can intensify feelings of fear and anxiety. Evidence is presented from human behavioural and neuroimaging studies. | Information about the state of cardiovascular arousal is transmitted to the brain periodically at individual heartbeats. Arterial baroreceptors within the aortic arch and carotid sinus communicate information about the timing and strength of each heartbeat. Afferent information is transmitted from the arterial baroreceptors along cranial nerves, namely the vagus nerve and the glossopharyngeal nerve, to the brainstem, and then relayed on to key brain structures implicated in affective and cognitive processing, including the insular cortex and the amygdala. Thus, changes in cardiovascular arousal may modulate emotional experiences. |
| **Name and date** | **Paper type** | **Study design/intervention/theory/review topic** | **Key relevant findings/utility to review** |
| **6.3 Arterial baroreceptors transmit information about cardiovascular arousal to brain regions implicated in affective & cognitive processing. (Continued).** | | | |
| Garfinkel & Critchley (2016)  (continued) |  |  | Specific effects associated with the firing of arterial baroreceptors identified in heart-timing experiments include: enhanced fear processing, enhanced amygdala activity, general inhibition of sensory processing, attenuated responses to painful stimuli. The degree to which the above effects occur varies across individuals, depending on “interoceptive sensitivity” or the degree to which a person is sensitive to changes in bodily state. Such variations may reflect traits determining an individual’s emotional style. |
| Hassanpour et al. (2016) | Empirical | An experimental study that used boluses of isoproterenol (a rapidly acting peripheral beta-adrenergic agonist similar to adrenaline) and brain imaging to identify the processes underlying the modulation of interoceptive states. During fMRI sessions, 21 healthy participants received four intravenous boluses (three different doses of isoproterenol and a control saline infusion). Drug administration was single blind and pseudorandomized. During each drug infusion scan, participants were requested to “pay attention to your ongoing experience of body sensations and emotions”. Outcome measures included cardiac and respiratory waveforms, self-reported changes in cardiovascular sensations, plus ratings of heartrate, breathing and anxiety intensity. Pharmacological fMRI was used to measure blood-oxygenation-level dependent (BOLD) and arterial spin labelling (ASL) signals. | The investigators reported that isoproternenol elicited dose-dependent increases in heart rate and cardiorespiratory sensations. Participants also retrospectively reported experiencing increased anxiety during the highest administered dose of isoproterenol (2 µg). fMRI data indicated dose-dependent increases in BOLD activation of the right insular cortex. The main finding of right insula activation supports neuroanatomically based theories that suggest a key role for the right insula in representing subjective awareness. |

**References**

Beckes, L., & Coan, J. A. (2011). Social baseline theory: The role of social proximity in emotion and economy of action. *Social and Personality Psychology Compass, 5,* 976–988. doi:10.1111/j.1751-9004.2011.00400.x

de Berker, A. O., Rutledge, R. B., Mathys, C., Marshall, L., Cross, G. F., Dolan, R. J., & Bestmann, S. (2016). Computations of uncertainty mediate acute stress responses in humans. *Nature Communications, 7,* 10996. doi:10.1038/ncomms10996

Berridge, K. C., & Robinson, T. E. (2003). Parsing reward. *Trends in Neurosciences, 26,* 507–513. doi:10.1016/S0166-2236(03)00233-9

Blevins, C. L., Sagui, S. J., & Bennett, J. M. (2017). Inflammation and positive affect: Examining the stress-buffering hypothesis with data from the National Longitudinal Study of Adolescent to Adult Health. *Brain, Behavior, and Immunity Journal, 61,* 21–26. doi:10.1016/j.bbi.2016.07.149

Bogdan, R., & Pizzagalli, D. A. (2006). Acute stress reduces reward responsiveness: Implications for depression. *Biological Psychiatry, 60,* 1147–1154. doi:10.1016/j.biopsych.2006.03.037

Bushnell, M. C., Ceko, M., & Low, L. A. (2013). Cognitive and emotional control of pain and its disruption in chronic pain. *Nature Reviews Neuroscience, 14,* 502–511. doi:10.1038/nrn3516

Cardoso, C., Kingdon, D., & Ellenbogen, M. A. (2014). A meta-analytic review of the impact of intranasal oxytocin administration on cortisol concentrations during laboratory tasks: Moderation by method and mental health. *Psychoneuroendocrinology, 49,* 161–170. doi:10.1016/j.psyneuen.2014.07.014

Case, L. K., Ceko, M., Gracely, J. L., Richards, E. A., Olausson, H., & Catherine Bushnell, M. (2016). Touch perception altered by chronic pain and by opioid blockade. *eNeuro, 3,* e0138-15.2016. doi:10.1523/ENEURO.0138-15.2016

Coan, J. A., & Sbarra, D. A. (2015). Social Baseline Theory: the social regulation of risk and effort. *Current Opinion in Psychology, 1,* 87–91. doi:10.1016/j.copsyc.2014.12.021

Coan, J. A., Schaefer, H. S., & Davidson, R. J. (2006). Lending a hand: Social regulation of the neural response to threat. *Psychological Science, 17,* 1032–1039. doi:10.1111/j.1467-9280.2006.01832.x

Creswell, D. J., Pacilio, L. E., Denton, T. F., & Satyshur, B. S. (2013). The effect of a primary sexual reward manipulation on cortisol responses to psychosocial stress in men. *Psychosomatic Medicine, 75,* 397–403. doi:10.1097/PSY.0b013e31828c4524

De Dreu, C. K. W., Greer, L. L., Van Kleef, G. A., Shalvi, S., & Handgraaf, M. J. J. (2011). Oxytocin promotes human ethnocentrism. *Proceedings of the National Academy of Sciences, 108,* 1262–1266. doi:10.1073/pnas.1015316108

Diener, E., Colvin, C. R., Pavot, W. G., & Allman, A. (1991). The psychic costs of intense positive affect. *Journal of Personality and Social Psychology, 61,* 492–503. doi:10.1037/0022-3514.61.3.492

Diener, E., Sandvik, E., & Pavot, W. (1991). Happiness is the frequency, not the intensity, of positive versus negative affect. In M. Argyle (Series Ed.), *International Series in Experimental Social Psychology: Vol. 21. Subjective well-being: An interdisciplinary perspective* (pp. 119–139). Oxford: Pergamon Press.

Field, T. (2016). Moderate pressure massage therapy. In H. Olausson, J. Wessberg, I. Morrison, & F. McGlone (Eds.), *Affective touch and the neurophysiology of CT afferents* (pp. 385–396). New York: Springer. doi:10.1007/978-1-4939-6418-5_22

Field, T., Diego, M., & Hernandez-Reif, M. (2010). Moderate pressure is essential for massage therapy effects. *International Journal of Neuroscience, 120,* 381–385. doi:10.3109/00207450903579475

Gard, D. E., Gard, M. G., Kring, A. M., & John, O. P. (2006). Anticipatory and consummatory components of the experience of pleasure: A scale development study. *Journal of Research in Personality, 40,* 1086–1102. doi:10.1016/J.JRP.2005.11.001

Garfinkel, S. N., & Critchley, H. D. (2016). Threat and the body: how the heart supports fear processing. *Trends in Cognitive Sciences, 20,* 34–46. doi:10.1016/j.tics.2015.10.005

Gerlach, K. D., Spreng, R. N., Madore, K. P., & Schacter, D. L. (2014). Future planning: default network activity couples with frontoparietal control network and reward-processing regions during process and outcome simulations. *Social Cognitive and Affective Neuroscience, 9*, 1942–1951. doi:10.1093/scan/nsu001

Goldstein, P., Shamay-Tsoory, S. G., Yellinek, S., & Weissman-Fogel, I. (2016). Empathy predicts an experimental pain reduction during touch. *The Journal of Pain, 17,* 1049–1057. doi:10.1016/j.jpain.2016.06.007

Gooding, D. C., & Pflum, M. J. (2014). The assessment of interpersonal pleasure: introduction of the Anticipatory and Consummatory Interpersonal Pleasure Scale (ACIPS) and preliminary findings. *Psychiatry Research, 215,* 237–243. doi:10.1016/j.psychres.2013.10.012

Habig, K., Schänzer, A., Schirner, W., Lautenschläger, G., Dassinger, B., Olausson, H., … Krämer, H. H. (2017). Low threshold unmyelinated mechanoafferents can modulate pain. *BMC Neurology, 17,* 184. doi:10.1186/s12883-017-0963-6

Hassanpour, M. S., Yan, L., Wang, D. J. J., Lapidus, R. C., Arevian, A. C., Simmons, W. K., … Khalsa, S. S. (2016). How the heart speaks to the brain: neural activity during cardiorespiratory interoceptive stimulation. *Philosophical Transactions of the Royal Society B: Biological Sciences, 371,* 20160017. doi:10.1098/rstb.2016.0017

Hertenstein, M. J., Keltner, D., App, B., Bulleit, B., & Jaskolka, A. R. (2006). Touch communicates distinct emotions. *Emotion, 6,* 528–533. doi:10.1037/1528-3542.6.3.528

Hertenstein, M. J., Verkamp, J. M., Kerestes, A. M., & Holmes, R. M. (2006). The communicative functions of touch in humans, nonhuman primates, and rats: A review and synthesis of the empirical research. *Genetic, Social, and General Psychology Monographs, 132,* 5–94. doi:10.3200/MONO.132.1.5-94

Human, L. J., Whillans, A. V., Hoppmann, C. A., Klumb, P., Dickerson, S. S., & Dunn, E. W. (2015). Finding the middle ground: Curvilinear associations between positive affect variability and daily cortisol profiles. *Emotion, 15*, 705–720. doi:10.1037/emo0000071

Kaada, B., & Torsteinbø, O. (1989). Increase of plasma β-endorphins in connective tissue massage. *General Pharmacology, 20,* 487–489. doi:10.1016/0306-3623(89)90200-0

Kut, E., Candia, V., von Overbeck, J., Pok, J., Fink, D., & Folkers, G. (2011). Pleasure-related analgesia activates opioid-insensitive circuits. *The Journal of Neuroscience, 31*, 4148–4153. doi:10.1523/JNEUROSCI.3736-10.2011

Ladak, A., Tubbs, R. S., & Spinner, R. J. (2014). Mapping sensory nerve communications between peripheral nerve territories. *Clinical Anatomy, 27,* 681–690. doi:10.1002/ca.22285

Liljencrantz, J., Strigo, I., Ellingsen, D. M., Krämer, H. H., Lundblad, L. C., Nagi, S. S., … Olausson, H. (2017). Slow brushing reduces heat pain in humans. *European Journal of Pain,* 21, 1173–1185. doi:10.1002/ejp.1018

Lindgren, L., Westling, G., Brulin, C., Lehtipalo, S., Andersson, M., & Nyberg, L. (2012). Pleasant human touch is represented in pregenual anterior cingulate cortex. *NeuroImage, 59,* 3427–3432. doi:10.1016/j.neuroimage.2011.11.013

Loseth, G. E., Ellingsen, D.-M., & Leknes, S. (2014). State-dependent μ-opioid modulation of social motivation. *Frontiers in Behavioral Neuroscience, 8,* 430. doi:10.3389/fnbeh.2014.00430

Macdonald, A. A., Naci, L., Macdonald, P. A., & Owen, A. M. (2015). Anesthesia and neuroimaging: investigating the neural correlates of unconsciousness*. Trends in Cognitive Sciences, 19,* 100–107. doi:10.1016/j.tics.2014.12.005

Mancini, F., Nash, T., Iannetti, G. D., & Haggard, P. (2014). Pain relief by touch: A quantitative approach. *Pain, 155,* 635–642. doi:10.1016/j.pain.2013.12.024

Mancini, F., Beaumonta, A.-L., Huc, L., Haggardb, P., & Iannettia, G. D. D. (2015). Touch inhibits subcortical and cortical nociceptive responses. *Pain, 153,* 1936–1944. doi:10.1097/j.pain.0000000000000253

McCracken, L. M., Zayfert, C., & Gross, R. T. (1992). The pain anxiety symptoms scale: development and validation of a scale to measure fear of pain. *Pain, 50,* 67–73. doi:10.1016/0304-3959(92)90113-P

Melzack, R. (2001). Pain and the neuromatrix in the brain. *Journal of Dental Education, 65,* 1378–1382. Retrieved from http://www.jdentaled.org/content/65/12/1378.full.pdf+html

Melzack, R., & Katz, J. (2013). Pain. *Wiley Interdisciplinary Reviews: Cognitive Science, 4,* 1–15. doi:10.1002/wcs.1201

Melzack, R., & Wall, P. D. (1965). Pain mechanisms: A new theory. *Science, 150,* 971–979. doi:10.1126/science.150.3699.971

von Mohr, M., Kirsch, L. P., & Fotopoulou, A. (2017). The soothing function of touch: affective touch reduces feelings of social exclusion. *Scientific Reports, 7,* 13516. doi:10.1038/s41598-017-13355-7

Morelli, S. A., Torre, J. B., & Eisenberger, N. I. (2014). The neural bases of feeling understood and not understood. *Social Cognitive and Affective Neuroscience, 9*, 1890–1896. doi:10.1093/scan/nst191

Morrison, I. (2016). CT afferent-mediated affective touch: Brain networks and functional hypotheses. In H. Olausson, J. Wessberg, I. Morrison, & F. McGlone (Eds.), *Affective touch and the neurophysiology of CT afferents* (pp. 195–208). New York: Springer. doi:10.1007/978-1-4939-6418-5_12

Nummenmaa, L., Tuominen, L., Dunbar, R., Hirvonen, J., Manninen, S., Arponen, E., … Sams, M. (2016). Social touch modulates endogenous μ-opioid system activity in humans. *NeuroImage*, *138*, 242–247. doi:10.1016/j.neuroimage.2016.05.063

Parducci, A. (1968). The relativism of absolute judgments. *Scientific American, 219*(6), 84–90. doi:10.1038/scientificamerican1268-84

Parducci, A. (1984) Value judgments: Towards a relational theory of happiness. In J.R. Eiser (Ed.), *Attitudinal Judgment* (pp. 3–21). New York: Springer-Verlag. doi: 10.1007/978-1-4613-8251-5_1

Peters, A., McEwen, B. S., & Friston, K. (2017). Uncertainty and stress: Why it causes diseases and how it is mastered by the brain. *Progress in Neurobiology, 156,* 164–188. doi:10.1016/J.PNEUROBIO.2017.05.004

Piva, M., & Chang, S. W. C. (2018). An integrated framework for the role of oxytocin in multistage social decision-making. *American Journal of Primatology*. Advanced online publication. doi: 10.1002/ajp.22735

Pizzagalli, D. A. (2014). Depression, stress, and anhedonia: Toward a synthesis and integrated model. *Annual Review of Clinical Psychology, 10,* 393–423. doi:10.1146/annurev-clinpsy-050212-185606

Pressman, S. D., & Cohen, S. (2005). Does positive affect influence health? *Psychological Bulletin, 131,* 925–971. doi:10.1037/0033-2909.131.6.925

Sharp, T. J., & Harvey, A. G. (2001). Chronic pain and posttraumatic stress disorder: mutual maintenance? *Clinical Psychology Review, 21,* 857–877. doi:10.1016/S0272-7358(00)00071-4

Sippel, L. M., Allington, C. E., Pietrzak, R. H., Harpaz-Rotem, I., Mayes, L. C., & Olff, M. (2017). Oxytocin and stress-related disorders: Neurobiological mechanisms and treatment opportunities. *Chronic Stress, 1.* doi:10.1177/2470547016687996

Solomon, R. L. (1980). The opponent-process theory of acquired motivation: The costs of pleasure and the benefits of pain. *American Psychologist, 35,* 691–712. doi:10.1037/0003-066X.35.8.691

Sullivan, M. J. L., Bishop, S. R., & Pivik, J. (1995). The pain catastrophizing scale: Development and validation*. Psychological Assessment, 7,* 524–532. doi:10.1037/1040-3590.7.4.524

Vallbo, Å., Löken, L., & Wessberg, J. (2016). Sensual touch: A slow touch system revealed with microneurography. In H. Olausson, J. Wessberg, I. Morrison, & F. McGlone (Eds.), *Affective touch and the neurophysiology of CT afferents* (pp. 1–30). New York: Springer. doi:10.1007/978-1-4939-6418-5_1

Walker, S. C., Trotter, P. D., Swaney, W. T., Marshall, A., & Mcglone, F. P. (2017). C-tactile afferents: Cutaneous mediators of oxytocin release during affiliative tactile interactions? *Neuropeptides, 64,* 27–38. doi:10.1016/j.npep.2017.01.001

Watanabe, N., & Hotta, H. (2017). Heart rate changes in response to mechanical pressure stimulation of skeletal muscles are mediated by cardiac sympathetic nerve activity. *Frontiers in Neuroscience, 10,* 614. doi:10.3389/fnins.2016.00614

Watanabe, N., Piché, M., & Hotta, H. (2015). Types of skin afferent fibers and spinal opioid receptors that contribute to touch-induced inhibition of heart rate changes evoked by noxious cutaneous heat stimulation. *Molecular Pain*, *11:*4, doi:10.1186/s12990-015-0001-
