# Supplementary material for: Interpersonal touch interventions for patients in intensive care: A design‐oriented realist review
Source: Nurs Open. 2018 Oct 24;6(2):216–35. doi: 10.1002/nop2.200 (PMC6419112; doi:10.1002/nop2.200)
Supplement: Supplementary file 9 [file NOP2-6-216-s009.docx]

**Appendix S9: Examples of design propositions**

**(context–intervention–mechanism–outcome configurations)**

**Example 1**

In Context *C^1^*: An individual has pain resulting from nociceptive signals transmitted from a specific anatomical region.

Use an intervention with characteristics *I^1^*: Moving touch applied at close proximity to location of nociceptive input.

To activate Mechanisms *M^1^*: Ascending inhibition of pain signals at the neural gate in the spinal cord by the stimulation of large diameter nerve fibres (Melzack & Wall, 1965).

To achieve Outcome *O^1^*: Reduce pain signalling from the spinal cord to the brain.

Rationales: (a) non-nociceptive signals travel faster than nociceptive signals and therefore close the gate in the spinal cord before the nociceptive signal arrives; (b) moving touch results in greater transmission of tactile (non-nociceptive) signals; (c) proximity of nociceptive and non-nociceptive signals is a key factor in activating this mechanism (Mancinia et al., 2015).

**Example 2**

In Context *C^2^*: A sedated patient for whom sedation can be safely be lightened.

Use Intervention *I^2^*: Lighten sedation to promote cortical activity and connectivity (Boveroux et al., 2010).

Activate mechanism *M^2^*: Increased cortical activity and connectivity promotes reward responding.

To achieve Outcome *O^2^*: Increased capacity for reward responding.

Outcome *O^2^* then functions as new Context *C^3^*:

In new Context *C^3^*: A patient for whom sedation has been safely lightened.

Use Intervention with characteristics *I^3^*: Medium-speed, gentle stroking applied to hairy skin, for example, the forearm. The interventionist should warm their hands if necessary before delivering intervention.

To activate mechanism *M^3^*: The stimulation of CT afferent nerve fibres activates brain regions associated with reward responding (Morrison, 2016).

To achieve Outcome *O^3^*: Increased pleasure.

Rationale: (a) CT afferents are present only in hairy skin and respond specifically to warm (skin temperature), medium-speed, gentle stroking touch (Ackerley et al., 2014; Löken et al., 2009; Vallbo et al., 1999).

**Example 3**

In Context *C^4^*: Patient has a close relative who expresses an interest in delivering an interpersonal touch intervention.

Use Intervention I^4^: Provide individualised support and information to the relative on how to deliver the interpersonal touch intervention.

To activate mechanisms *M^4&5^*: Increased family member’s knowledge (*M^4^*) and confidence (*M^5^*) in delivering intervention.

To achieve Outcome *O^4^*: Supported delivery of the touch intervention by a close family member.

Outcome O^4^ then functions as new context *C^5^*.

In Context *C^5^*: Patient perceives a high quality relationship to the touch interventionist.

Use Intervention *I^5^*: Interpersonal touch.

Activate Mechanism *M^6^*: The patient receiving the touch construes psychosocial resources as bioenergetics resources (Coan & Sbarra, 2015).

To achieve outcome *O^5^*: Attenuated physiological threat response and reduced neural and peripheral metabolic demands (Beckes & Coan, 2011).

**Example 4**

In Context *C^6^*: A patient with previous experience of the intervention.

Use Intervention with characteristics *I^6^*: An intervention repeated at frequent intervals.

To activate Mechanism *M^7^*: Positive neural interactions between reward components “liking”, “wanting”, and “learning” (Berridge & Robinson, 2003).

To achieve Outcome *O^6^*: Increased reward responding.

**Example 5**

In Context *C^7^*: A patient with high levels of perceived psychological stress.

Use Intervention with characteristics *I^7^*: An intervention that provides frequent episodes of moderate intensity positive affect.

To activate Mechanism *M^8^*: Lower average frequency of negative affect and higher average frequency of positive affect (Blevins et al. 2017; Diener et al*.* 1991; Pressman & Cohen, 2005).

To achieve Outcome *O^7^*: Reduced stress response.

**Example 6**

In Context *C^8^*: The patient is sedated and has an elevated heart rate resulting from stress and anxiety.

Use an Intervention with characteristics *I^8^*: Moderate pressure massage.

To activate Mechanism *M^9^*: Stimulation of dermal and subdermal pressure receptors activates structures within the autonomic nervous system (Field, 2016; Field et al., 2010).

To achieve Outcome *O^8^*: Increased PNS activity and/or reduced SNS activity.

Rationale: the mechanism is less reliant on cortical activity and connectivity (mechanisms underlying gentle touch; see Example 2), which are relatively suppressed by sedative drugs compared with autonomic activity.

**References**

Ackerley, R., Backlund Wasling, H., Liljencrantz, J., Olausson, H., Johnson, R. D., & Wessberg, J. (2014). Human C-tactile afferents are tuned to the temperature of a skin-stroking caress. *The Journal of Neuroscience*, *34*(8), 2879–83. doi:10.1523/JNEUROSCI.2847-13.2014

Beckes, L., & Coan, J. A. (2011). Social baseline theory: The role of social proximity in emotion and economy of action. *Social and Personality Psychology Compass, 5,* 976–988. doi:10.1111/j.1751-9004.2011.00400.x

Berridge, K. C., & Robinson, T. E. (2003). Parsing reward. *Trends in Neurosciences*, *26*(9), 507–513. doi:10.1016/S0166-2236(03)00233-9

Blevins, C. L., Sagui, S. J., & Bennett, J. M. (2017). Inflammation and positive affect: Examining the stress-buffering hypothesis with data from the National Longitudinal Study of Adolescent to Adult Health. *Brain, Behavior, and Immunity Journal*, *61*, 21–26. doi:10.1016/j.bbi.2016.07.149

Boveroux, P., Vanhaudenhuyse, A., Bruno, M.-A., Noirhomme, Q., Lauwick, S., Luxen, A., … Boly, M. (2010). Breakdown of within- and between-network resting state functional magnetic resonance imaging connectivity during propofol-induced loss of consciousness. *Anesthesiology*, *113*(5), 1038–1053. doi:10.1097/ALN.0b013e3181f697f5

Coan, J. A., & Sbarra, D. A. (2015). Social Baseline Theory: the social regulation of risk and effort. *Current Opinion in Psychology, 1,* 87–91. doi:10.1016/j.copsyc.2014.12.021

Diener, E., Sandvik, E., & Pavot, W. (1991). Happiness is the frequency, not the intensity, of positive versus negative affect. In F. Strack, M. Argyle, & N. Schwarz (Eds.), *Subjective Well-Being: An Interdisciplinary Perspective (International Series in Social Psychology)* (pp. 119–139). Oxford7: Pergamon Press.

Field, T. (2016). Moderate Pressure Massage Therapy. In H. Olausson, J. Wessberg, I. Morrison, & F. McGlone (Eds.), *Affective Touch and the Neurophysiology of CT Afferents* (pp. 385–396). New York, NY: Springer New York. doi:10.1007/978-1-4939-6418-5_22

Field, T., Diego, M., & Hernandez-Reif, M. (2010). Moderate pressure is essential for massage therapy effects. *International Journal of Neuroscience*, *120*(5), 381–385. doi:10.3109/00207450903579475

Löken, L. S., Wessberg, J., Morrison, I., McGlone, F., & Olausson, H. (2009). Coding of pleasant touch by unmyelinated afferents in humans. *Nature Neuroscience*, *12*(5), 547–548. doi:10.1038/nn.2312

Mancinia, F., Beaumonta, A.-L., Huc, L., Haggardb, P., & Iannettia, G. D. D. (2015). Touch inhibits subcortical and cortical nociceptive responses. *Pain*, *153*, 1936–1944. doi:10.1097/j.pain.0000000000000253

Melzack, R., & Wall, P. D. (1965). Pain mechanisms: A new theory. *Science*, *150*(3699), 971–979. doi:10.1126/science.150.3699.971

Morrison, I. (2016). CT Afferent-mediated affective touch: Brain networks and functional hypotheses. In H. Olausson, J. Wessberg, I. Morrison, & F. McGlone (Eds.), *Affective touch and the neurophysiology of CT afferents* (pp. 195–208).

Pressman, S. D., & Cohen, S. (2005). Does positive affect influence health? *Psychological Bulletin*, *13*(6), 925–971. doi:10.1037/0033-2909.131.6.925

Vallbo, A. B., Olausson, H., & Wessberg, J. (1999). Unmyelinated afferents constitute a second system coding tactile stimuli of the human hairy skin. *Journal Neurophysiology*, *81*(6), 2753–2763. doi:10.1007/978-3-0348-9016-8_24
